# Structural Analysis of Anti-Hapten Antibodies to Identify Long-Range Structural Movements Induced by Hapten Binding

**DOI:** 10.3389/fmolb.2021.633526

**Published:** 2021-03-24

**Authors:** Mohammed M. Al Qaraghuli, Karina Kubiak-Ossowska, Valerie A. Ferro, Paul A. Mulheran

**Affiliations:** 1 Department of Chemical and Process Engineering, University of Strathclyde, Glasgow, United Kingdom; 2 SiMologics Ltd., The Enterprise Hub, Glasgow, United Kingdom; 3 Department of Physics, University of Strathclyde, Glasgow, United Kingdom; 4 Strathclyde Institute of Pharmacy and Biomedical Sciences, University of Strathclyde, Glasgow, United Kingdom

**Keywords:** antibody, antigen binding fragments (Fab), hapten, RMSD, RMSF, allosteric movement

## Abstract

Antibodies are well known for their high specificity that has enabled them to be of significant use in both therapeutic and diagnostic applications. Antibodies can recognize different antigens, including proteins, carbohydrates, peptides, nucleic acids, lipids, and small molecular weight haptens that are abundantly available as hormones, pharmaceuticals, and pesticides. Here we focus on a structural analysis of hapten-antibody couples and identify potential structural movements originating from the hapten binding by comparison with unbound antibody, utilizing 40 crystal structures from the Protein Data Bank. Our analysis reveals three binding surface trends; S1 where a pocket forms to accommodate the hapten, S2 where a pocket is removed when the hapten binds, and S3 where no pockets changes are found. S1 and S2 are expected for induced-fit binding, whereas S3 indicates that a pre-existing population of optimal binding antibody conformation exists. The structural analysis reveals four classifications of structural reorganization, some of which correlate to S2 but not to the other binding surface changes. These observations demonstrate the complexity of the antibody-antigen interaction, where structural changes can be restricted to the binding sites, or extend through the constant domains to propagate structural changes. This highlights the importance of structural analysis to ensure successful and compatible transformation of small antibody fragments at the early discovery stage into full antibodies during the subsequent development stages, where long-range structural changes are required for an Fc effector response.

## Introduction

The immune system represents a major defensive mechanism that protects vertebrates against pathogen invasion. Within this system, lymphocytes synthesize cell surface receptors or secrete glycoproteins, known as antibodies, which specifically bind to foreign molecules (Alberts et al., 2002). Antibodies are immunoglobulins that can recognize various types of antigen, including proteins, carbohydrates, peptides, nucleic acids, lipids, and small molecular weight (<1,000 Da) haptens. The focus of this article will be on haptens since they are ubiquitously found as hormones, pharmaceuticals, and pesticides (Al Qaraghuli et al., 2015).

An antibody of class IgG is normally composed of two light and two heavy chains linked together by disulphide bonds. These heavy and light chains create two identical antigen-binding fragments (Fabs), each contain the first two domains of the heavy (V_H_ and C_H1_) and light (V_L_ and C_L_) chains, and one crystallizable region fragment (Fc), each comprising C_H2_ and C_H3_ domains (Porter, 1959; Ryle and Porter, 1959). The Fc region can propagate a series of immunological responses through the heavy chains, whilst the Fab fragments are responsible for antigen recognition through variable sites known as complementarity determining regions (CDRs) (Kubota et al., 2009). Each variable (V_H_ and V_L_) domain contains three CDRs, and the resulting six CDRs collectively represent the antigen binding sites.

The antibody-antigen interaction process is dictated by a multitude of non-covalent forces (Van Oss, 1995), and supported by complementarity in the binding interface charge and shape (Al Qaraghuli et al., 2015). The surface topography of these binding sites normally differs according to the antigen type (Webster et al., 1994). Somatic gene-recombination and mutations, heavy and light chain dimerization, and antibody class-switching, are mechanisms that can craft the malleable binding sites to accommodate a wide range of antigens (Jacob et al., 1991; MacLennan, 1991; Muramatsu et al., 2000; Abhinandan and Martin, 2010). These shapes include flat surfaces to grasp large protein surfaces, pockets to accommodate small haptens, and grooves to enclose peptides (MacCallum et al., 1996).

Even with the well-known specificity of antibodies, some antigen-specific antibodies can still simultaneously interact with different structurally unrelated antigens (Bentley et al., 1990; Arevalo et al., 1993; Pokkuluri et al., 1994; Keitel et al., 1997; Krishnan et al., 2008; Tapryal et al., 2013). This antibody poly-specificity could be a method implemented by the body to enlarge the immune-system conformational repertoire and enhance foreign pathogen recognition. Different variability-upon-binding phenomena are frequently described, like ligand induced-fit: the binding of an antibody to an antigen causes a change in the shape of the antibody binding sites to enhance the binding efficiency (Rini et al., 1992; Bosshard, 2001; Rosen et al., 2005; Keskin, 2007; Wang et al., 2013); antibody isomerism: preferential ligand binding to a pre-existing subpopulation of antibody isomers that exist in equilibrium (Foote and Milstein, 1994; Hansson et al., 1997; Foote, 2003; Debler et al., 2008); promiscuity: the ability of an antibody to bind to structurally different antigens through different binding sites (Backes et al., 2003; James et al., 2003; James and Tawfik, 2003; Dimitrov et al., 2007, 2012; Kang and Warren, 2007; Bryson et al., 2009); and moonlighting: an antibody having different functions results from binding to different antigens (Martin, 2014; Irani, 2016). These phenomena can be facilitated through allosteric structural movements of the antibody loops (Piekarska et al., 2006; Sela-Culang et al., 2012; Bowen and Casadevall, 2016; Yang et al., 2017), which can propagate signals from variable to constant regions to induce Fc-mediated effector functions. The generated effector functions can then induce antibody-dependent cell-mediated cytotoxicity (ADCC) and complement-dependent cytotoxicity (CDC).

Despite the significant interest by the biopharmaceutical industry to generate and craft antibodies’ binding sites with the highest affinity, it is equally crucial to understand the conformational changes beyond these binding sites. This will ensure successful and compatible transformation of small antibody fragments (single-chain variable fragment (scFv) or Fab fragments) at early developmental stages to full antibodies. Accordingly, understanding the allosteric model might assist us to comprehend the structural signaling that occurs beyond the pocket binding sites of anti-hapten antibodies.

In a recent study, we examined the structural differences in anti-protein Fab crystal structures caused by binding to the antigens (Al Qaraghuli et al., 2020). This revealed three classifications for the observed changes that we called class B1, B2 and B3. In B1, the binding of the protein antigen to the Fab causes a distortion to the Fab’s diamond-like structure along with a movement in the linker region that connects the Fab to the Fc in the full antibody. This is the expected behavior when a large protein interacts across the Fab binding surface, resulting in significant changes that propagate through the heavy chains to create a potential allosteric signal. However, in class B2 we found no pronounced distortion of the Fab but still recognized the potential allosteric signal. Class B3 was even more surprising, with no distortion and no apparent structural movements, although we note that this was found for only one protein antigen that was derived from synthetic phage display library without any requirement for an effector response in its development.

In this paper, we extend our analysis to anti-hapten Fab crystal structures of IgG antibodies retrieved from the Protein Data Bank (PDB). These Fabs were crystallized in free and antigen-complexed formats. It is well known that anti-hapten antibodies display pocket binding-sites to accommodate small molecular weight haptens. However, it is not completely clear how these pockets are generated in response to these haptens. Therefore, it was necessary to analyze the binding surfaces of these antibodies, and classify them into different surface (S) classes accordingly. Moreover, haptens are expected to cause smaller changes in the antibody binding sites when compared to large molecular weight proteins. So another essential point to investigate is related to whether our previous binding (B) classification, conducted on anti-protein antibodies (Al Qaraghuli et al., 2020), can be extended to haptens. Our analyses have also included comparison of the B and S classes to identify any favorable correlation among these classes. Together, these provide a comprehensive insight to the different effects hapten binding has on antibody structure. In particular, the recognition of potential conformational changes can provide crucial information for the successful development of novel antibodies, since the absence of long-range structural changes beyond the binding site indicate no concomitant changes to functionality and thus no allostery.

## Methods

### Antibody Selection and Sequence Analysis

Forty crystal structures of Fab fragments were retrieved from the PDB and organized into couples, so that free antibody could be compared to the hapten antigen-bound form. We included all structures with acceptable resolution (≲3°Å) in the analysis to allow a confident classification of the surface topography and structural changes (McDonald and Thornton, 1994; Almagro, 2004; Raghunathan et al., 2012)^.^ Antibody structures were considered similar if their chains (heavy and light) shared 100% sequence identity, and had similar sequence to the antigen bound form. The effect of crystal contacts in the PDB structures used in this analysis were considered negligible, because the structural changes were analyzed in terms of backbone movements rather than the side-chains that are most likely affected by crystal formation. Therefore, the assumption is made that the crystal contacts in the PDB structures do not strongly affect the backbone structure; this assumption can be explored further in future work.

Amino acid positions were sequentially numbered to avoid conflicts at residue positions, and to ensure accurate structural measurements. A few sequences were omitted from the study at different stages (such as 1JNH, 1JN6, and 1JGL) due to the fact that these polypeptide chains were not continuous. In total, 40 crystal structures (32 mouse, four fully human, and four chimeric with human C_H1_ domain) were identified and selected; all the potential sequences in the PDB were considered, so that the analysis is comprehensive within the stated selection criteria. A few of the selected antibodies were crystalized against more than one antigen, and full details of these crystal structures are summarized in Supplementary Tables S1.1.

The Fab sequences were retrieved from the PDB, and analyzed using BioEdit Sequence Alignment Editor, version 7.2.5 (Hall, 1999). ClustalW Multiple alignment was used to align sequences of the same type (heavy or light chains) and origin (human or mouse). All the gathered and analyzed sequences are listed in Supplementary Tables S3. The three loops in the variable domains were denoted CDRs 1–3, and the three loops in the constant domains (CH and CL) were named C_Loop 1–3, according to their location throughout the sequence from the N-terminal to the C-terminal of the entire chain.

### Root-Mean-Square Distance (RMSD) and Fluctuations (RMSF)

The structural variability of similar proteins or different conformations of the same protein can be quantified using RMSD (Carugo and Pongor, 2001).

Here the RMSD is defined as

$$RMSD=\sqrt{\frac{\sum_{i=1}^{N}{\left|{\vec{r}}_{i,A}-{\vec{r}}_{i,F}\right|}^{2}}{N}}$$ (1)

where N is the number of (backbone) atoms in the protein structure and ${\vec{r}}_{i,A}$ , ${\vec{r}}_{i,F}$ is the position of the *i*th atom in the antigen-bound (A) and free (F) Fab structure. In order to calculate the RMSD, the two protein structures to be examined are firstly considered as rigid bodies, then overlapped (aligned) using only translations and rotations. The RMSDs of the backbone atoms were only calculated for particular regions, such as variable (residues 1–105), linker (106–113) and constant (114–218) domains, as well as each couple for the entire antibody chain (residues 1–218). The side chains of residues were excluded because insignificant differences in their orientations could introduce additional noise.

RMSF is another useful tool to track structural changes in proteins, where the RMSD is calculated and reported for each protein residue in the compared structure. It is regularly denoted as “fluctuations” as it calculates a time-average amplitude of residue movement (fluctuation) from each compared position in the aligned structures. Despite the wide adaptation of RMSF in dynamic structures, it can be used in static structures as it provides RMSDs calculated per particular residue. With this form of RMSF, it is easy to track the residues that are most responsible for the total conformational alterations, the creation of pockets and, for potential allosteric signaling. Therefore, following Eq. 1 we define RMSF in Eq. 2, and where symbols have the same meaning as before.

$$RMSF_{i}=\left|{\vec{r}}_{i,A}-{\vec{r}}_{i,F}\right|$$ (2)

### Variable and Constant Domain Orientation

**FIGURE 1:**
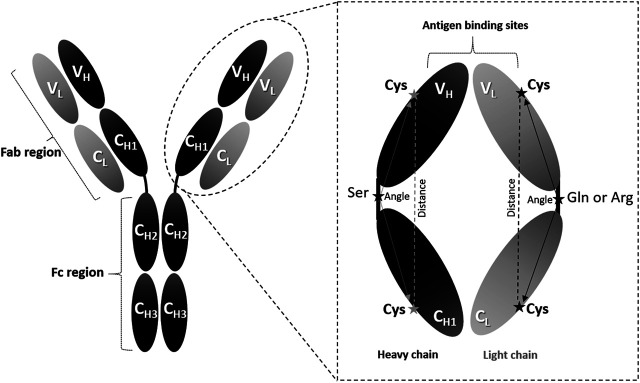
Angle measurements. The IgG antibody can be divided into three fragments: two Fab regions and one Fc region. The enlarged Fab region illustrates the amino acid positions used to calculate the domain orientational changes. The selected amino acids are denoted as Cys: cysteine, Ser: serine, Gln: glutamine (in *λ* light chains), and Arg: arginine (in K light chains).

The orientation of the V_H_/V_L_ domains vs. C_H1_/C_L_ domains was examined to complement the RMSD/RMSF measurements. One conserved cysteine residue (Cys) was chosen in each of the V_L_, C_L_, V_H_, and C_H1_ domains (See *Domain Orientation*). Moreover, one conserved amino acid in each of the V_L_/C_L_ and V_H_/C_H1_ linkers was also selected. These amino acids were serine (Ser), arginine (Arg), and glutamine (Gln) in the heavy, light (K), and light (*λ*) chains, respectively (Figure 1). The six amino acids selected in each Fab are highlighted in turquoise in the full sequences file (Supplementary S3). The angles of the heavy (Cys-Ser-Cys) and light (Cys-Gln-Cys or Cys-Arg-Cys) chains were measure by PyMOL (The PyMOL Molecular Graphics System, Version 1.7.4 Schrödinger, LLC.). The distances between the Cys residues in the heavy and light chains were also measured by PyMOL.

## Results and Discussion

### Antibody Binding Surfaces

Analysis of anti-hapten Fabs can provide information about the changes in the binding surfaces upon antigen recognition. Anti-hapten antibodies have shown preference to recognize their targeted antigens through the formation of pockets in their binding sites. Comparison of the free vs. antigen-bound crystal structures Fabs (23 couples) have shown three binding surface (S1–3) trends:

• **S1:** the presence of a pocket binding site on the antibody-antigen complex but not on the antibody-free counterparts, as can be observed in three mouse Fab couples (1Q0Y_1Q0X, 3CFB_3CFC, and 1I9J_1I9I) and two human Fab couples (1D6V_1D5B and 1AJ7_2RCS). Illustration of these figures can be observed in Supplementary Figures S2.1, S2.10, S2.18, S2.22, and S2.23.
• **S2:** the presence of a pocket binding site on the antibody-free structure, but not on the antibody-antigen complex, as observed in three couples of mouse Fabs (1QYG_1RFD, 1Q72_1RFD, and 2AJV_2AJU). Supplementary Figures S2.4A, S2.5, and S2.12 show these three couples.
• **S3:** there is no (or slight) apparent change in the binding sites as can be noticed in the remaining 15 couples of anti-hapten Fabs.

**FIGURE 2:**
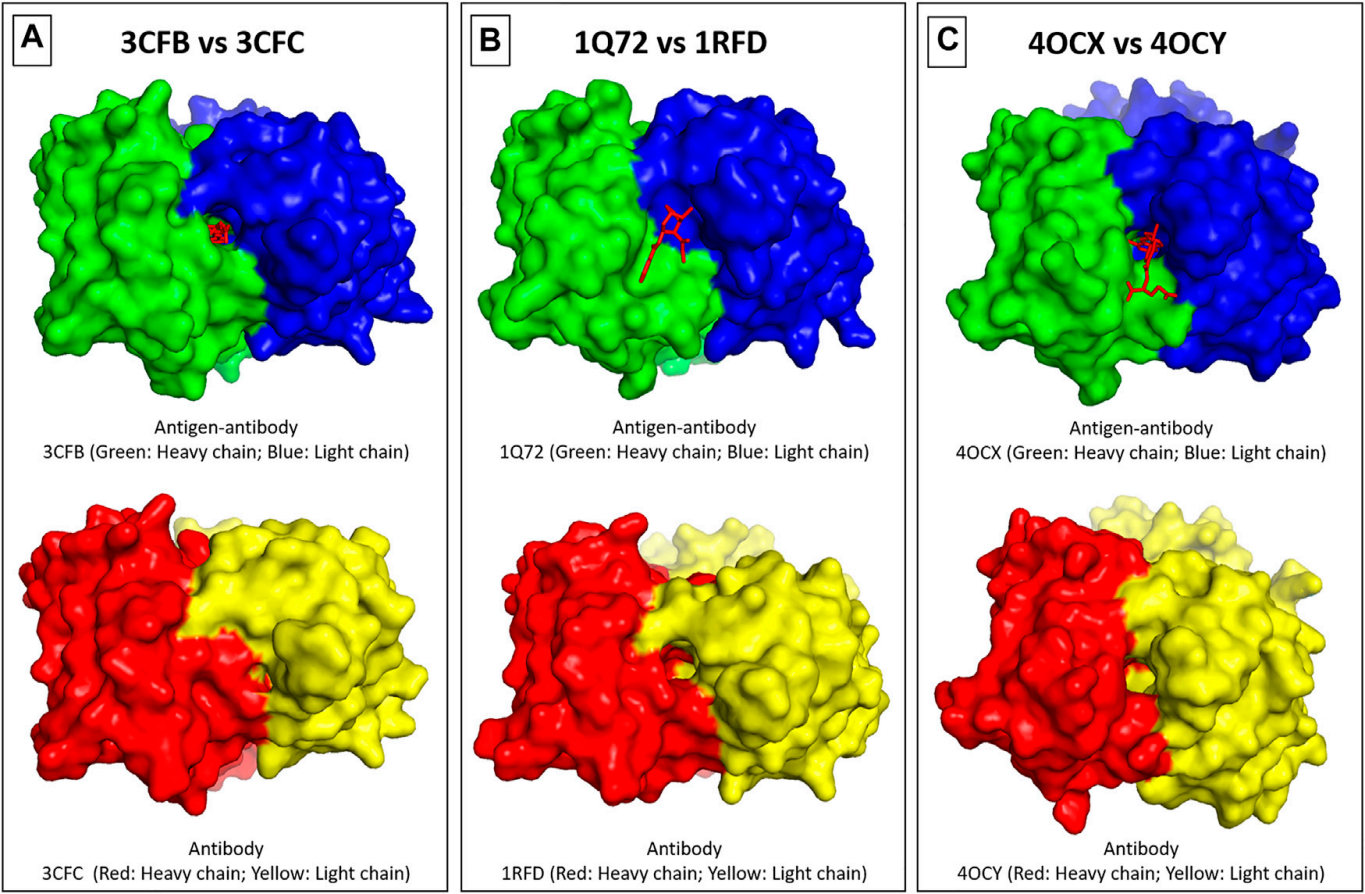
Binding sites surfaces. The binding surfaces can be grouped into three categories. In S1, there is a pocket binding site on the antibody-antigen complex and not on the antibody-free counterparts, as shown in **(A)**. In S2, there is a pocket binding site on the antibody-free structure and not on the antibody-antigen complex, as can be observed in **(B)**. In S3, there is no (or only slight) change in the binding sites as shown in **(C)**. The heavy and light chains were colored as denoted in the figure, and the hapten antigen is shown as a red line drawing.

An example from each of the three groups is demonstrated in Figure 2, and detailed observations are shown in Supplementary Figure S2. The antibody (3CFB; Figure 2A) can bind the antigen [4-(4-styryl-phenylcarbamoyl)-butyric acid] through side chain of amino acid Q96, and CDR H3 of the bound form has slightly moved inwards to support the pocket formation, as illustrated in Supplementary Figure S.2.1F. In contrast, the antigen (cocaine) can bind the antibody (1Q72; Figure 2B) through residue Y32 of the light chain, and CDR H3 of the bound form has moved away from the binding site center to cause an opening of the binding pocket (Supplementary Figure S.2.4F). Whilst, the third example was demonstrated by the antibody (4COX; Figure 2C) that can bind the antigen (methotrexate) through two amino acids at the light chain (E39 and Y41) and one amino acid at the heavy chain (N36) (Supplementary Figure S.2.17F). The three residues are located at the bottom of the pocket, and no major changes were noticed by comparing the antigen-bound verses the free form of the antibody.

Antibodies can generally bind antigens through multiple conformational states rather than a rigid lock and key method. Although structural changes between free and antigen-bound antibodies were usually correlated with an induced-fit mechanism, structural evidence for a pre-existing equilibrium was also identified (James et al., 2003). This was evident in analyzing anti-IL1β and anti-IL6 Fabs and scFvs that have shown the core of the antigen binding sites, in particular the CDR3 loops, to exist in multiple conformational states (Addis et al., 2014). This behavior seems to be a conserved feature of many high affinity protein-protein interaction sites, suggesting a key role in the formation of tight protein complexes (Waters et al., 2007; Veverka et al., 2009; Holdsworth et al., 2012). Consequently, slight structural changes between the free and bound structures would be expected if the free conformation that was crystallized is the same one bound to the antigen. These slight changes were observed in group S3 (15 couples), supporting this picture.

On the other hand, significant changes are expected if a different pre-existing antigen-unbound conformation was crystallized. These structural changes could reflect the anti-hapten Fabs of groups S1 (5 couples) and S2 (3 couples). A distinction between these two groups can be achieved through analysis of their RMSD/RMSF differences, and heavy and light chain packing (see below).

### Conformational Changes Upon Binding (RMSD Analysis)

The conformational changes were determined by calculating the total RMSD difference between the antibody-antigen complex and the antibody-free counterparts. Out of the analyzed 23 couples, seven have shown more deviations in the light chains when compared to heavy chains, and the opposite was observed in the remaining 16 couples (Table 1). In addition, the constant domains (C_H1_ and C_L_) of these Fabs have demonstrated more movements than the variable domains (V_H_ and V_L_), as reported in Table 1; and the quantitative information is summarized in Supplementary Table S1.2.

**TABLE 1:** RMSD comparison.

Heavy/light chains	Anti-hapten (23 couples)
Light chain > heavy chain	7 (3CFD_3CFE, 1JNN_1JNL, 1KEL_1KEM, 2AJY_2AJU, 2AK1_2AJU, 2AJV_2AJU, and 4OCX_4OCY)
Light chain < heavy chain	Remaining 16 couples

**Light chain**	
CL < VL	9 (3CFB_3CFC, 3CFD_3CDE, 1KEL_1KEM, 1Q0Y_1Q0X, 2AJX_2AJU, 2AJY_2AJU, 2AK1_2AJU, and 2AJV_2AJU, 2AJZ_2AJU)
CL = VL	1 (2AJS_2AJU)
CL > VL	Remaining 13 couples

**Heavy chain**	
CH < VH	7 (1UB5_1UB6, 2AJS_2AJU, 2AJX_2AJU, 2AJY_2AJU, 2AK1_2AJU, and 2AJV_2AJU, and 2AJZ_2AJU)
CH > VH	Remaining 16 couples

The high movements of the constant domains relative to the variable domains and to each other can increase the likelihood of a suitable V_H_-V_L_ relative orientation and signal propagation from the variable to constant domains (Padlan, 1994; Wilson and Stanfield, 1994; Braden and Poljak, 1995). For instance, these movements were reflected in the complement activation of two antibodies that differed only in their V_H_ domain (Horgan et al., 1992). In other work, two human monoclonal antibodies, having similar variable domains but different constant domains, were shown to bind their target with significantly different affinities (Pritsch et al., 1996). Furthermore, a comparison of the binding affinity of Fabs to smaller variable fragments (scFv) by Adachi et al. also revealed a large difference in affinity (Adachi et al., 2003). We have noticed these movements through analysis of an entire antibody in molecular dynamics simulations (Al Qaraghuli et al., 2018), and in a comprehensive analysis of anti-protein antibodies (Al Qaraghuli et al., 2020). So our findings suggest that the high movement of the constant domains could be a reflection of essential structural movements that can help trigger the immune response. These movements are expected to be greater in the heavy chain when compared to light chains, since the Fab-to-Fc movements occur in these heavy chains and through the main Fab-Fc linker.

### Conformational Changes Upon Binding (RMSF Analysis)

The RMSF of each amino acid position in the 40 crystal structures, which represent 23 free antibody and antigen-complexed couples, were also analyzed. In general, four different classifications (B1–4) for structural change upon binding were identified, following the classification scheme identified with anti-protein Fabs (Al Qaraghuli et al., 2020):

• **B1:** Random movements throughout the entire Fab illustrated by five anti-hapten couples (1NGP_1NGQ, 2CGR_1CGS, 1D6V_1D5B, 1AJ7_2RCS, and 1FL6_1FL5).
• **B2:** Specific movement at the C_Loop_1_ of six anti-hapten couples (3CFB/3CFC, 4OCX_4OCY, 1I9J_1I9I, 3LS4_3LS5, 1KEL_1KEM, and 1C5C_1C5B).
• **B3:** Specific movements at the CDRs of eight anti-hapten couples (2AJV_2AJU, 2AJS_2AJU, 2AJX_2AJU, 2AJY_2AJU, 2AJZ_2AJU, 2AK1_2AJU, 1Q72_1RFD, and 1QYG_1RFD).
• **B4:** Random, but very low movements as shown in four anti-hapten couples 3CFD_3CFE, 1UB5_1UB6, 1JNN_1JNL, and 1Q0Y_1Q0X.

**FIGURE 3:**
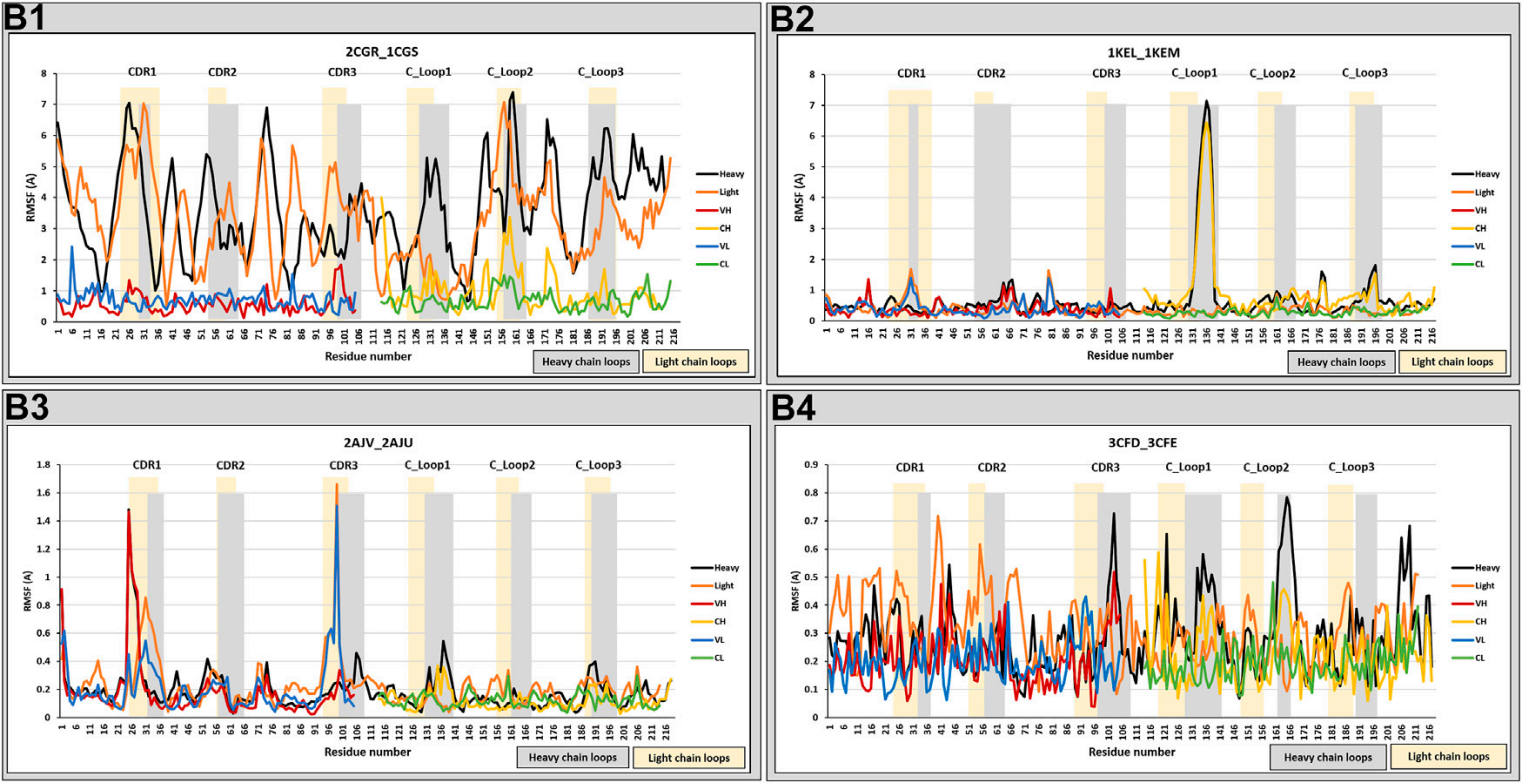
RMSF analysis. RMSF measurements were analyzed for anti-hapten Fabs. Classes B1, B2, B3, and B4 are represented by 2CGR_1CGS, 3CFD_3CFE, 2AJV_2AJU, and 1KEL_1KEM, respectively. The RMSF comparison was performed on the total chain (heavy and light) and independently on their specific domains (V_H_, C_H1_, V_L_, and C_L_). Heavy chain loops were highlighted in grey and light chain loops were highlighted in yellow. The *x*-axis denotes the amino acid positions of either the heavy or light chain, permitting them to be examined in the same figure.

Examples from each trend were selected from the anti-hapten Fabs, as illustrated in Figure 3, and Supplementary Figure S2. The RMSF was calculated for the entire heavy and light chains, and also separately for their specific domains (VH, CH, VL, and CL) to separate out contributions from the domain orientation changes reported below.

These results can reflect changes in the constant domains as well as the variable domains. These changes, however, were either random, with big (B1) or small (B4) movements, or at specific positions (CDRs and/or C_Loop1 in classes B3/B2). We note that this range of structural change agrees with our previous analysis of protein-binding Fabs, with the addition of B4 which is unique to certain anti-hapten Fabs.

Movements at the CDRs are expected to reflect the antigen binding process. Furthermore, Sela-Culang et al. (2012) have shown that the C_Loop_1_ was the highest moving loop, which in B2 has moved even more than CDRH3 in B3. Various studies have correlated the C_Loop1, which is located at C_H1_ domain, to detected changes in binding, because it was recognized as the only region with sequence diversity among the examined antibodies (Pritsch et al., 1996; Pritsch et al., 2000; Torres et al., 2007). Furthermore, the C_Loop_1_ was suggested to be intrinsically disordered, associated in the interaction between the heavy and light chains, and linked to complement binding (Sela-Culang et al., 2012). We note that this loop is close to the main hinge region, and a conformational change in this loop may affect the relative orientation of the C_H1_ domain vs. the entire Fc, thus influencing the overall effector function of the Fc region (Patil et al., 2010; Sela-Culang et al., 2012). Therefore, structural changes in the C_Loop_1_ (in the Fab region) could stimulate changes in the Fc region to facilitate triggering of the immune response. The random movements of B1 and B4 can be better understood through an analysis of the various domain orientations, as will be discussed in the following section.

### Domain Orientation

The heavy and light chain angles (Figure 1) of the anti-hapten Fabs have shown large changes in class B1, as observed in 1NGP/1NGQ, 2CGR/1CGS, 1FL6/1FL5, 1D6V/1D5B and 1AJ7/2RCS (Table 2 and Supplementary Table S4). These large changes originate from a reduction in the angles between the heavy chains and an increase between the light chains, and are in line with the distance changes between the selected Cys residues on each domain. The linker-linker distance of this class has also increased in the bound form when compared to their free counterparts. The small angle changes of class B4 were also reflected in the relatively low changes in the Cys-Cys and linker-linker distances. Likewise, both class B3 (with large CDR movement) and class B2 (large C_Loop1 movement) have shown relatively low angle changes, and this can be observed in the remaining crystal structure pairs (Supplementary Table 4).

**TABLE 2:** Binding conformational change classes.

	PDB ID	Crystal form	Magnitude of average angle change (^o^)	Binding surface class	Structural change class
1	3CFB	Antigen-antibody	9.25	S1	B2
2	3CFC	Antibody			
3	3CFD	Antigen-antibody	0.55	S3	B4
4	3CFE	Antibody			
5	1UB5	Antigen-antibody	0.95	S3	B4
6	1UB6	Antibody			
7	1Q72	Antigen-antibody	0.75	S2	B3
8	1QYG	Antigen-antibody	0.45	S2	B3
9	1RFD	Antibody			
10	1JNN	Antigen-antibody	0.75	S3	B4
11	1JNL	Antibody			
12	1KEL	Antigen-antibody	1	S3	B2
13	1KEM	Antibody			
14	1NGP	Antigen-antibody	8.55	S3	B1
15	1NGQ	Antibody			
16	2CGR	Antigen-antibody	19.75	S3	B1
17	1CGS	Antibody			
18	1Q0Y	Antigen-antibody	0.65	S1	B4
19	1Q0X	Antibody			
20	2AJS	Antigen-antibody	2.35	S3	B3
21	2AJV	Antigen-antibody	0.95	S2	B3
22	2AJX	Antigen-antibody	4.7	S3	B3
23	2AJY	Antigen-antibody	4.2	S3	B3
24	2AJZ	Antigen-antibody	1.85	S3	B3
25	2AK1	Antigen-antibody	4.15	S3	B3
26	2AJU	Antibody			
27	4OCX	Antigen-antibody	1.25	S3	B2
28	4OCY	Antibody			
29	1I9J	Antigen-antibody	0.5	S1	B2
30	1I9I	Antibody			
31	3LS4	Antigen-antibody	1.25	S3	B2
32	3LS5	Antibody			
33	1FL6	Antigen-antibody	5.9	S3	B1
34	1FL5	Antibody			
35	1C5C	Antigen-antibody	0.5	S3	B2
36	1C5B	Antibody			
37	1D6V	Antigen-antibody	22.4	S1	B1
38	1D5B	Antibody			
39	1AJ7	Antigen-antibody	30.65	S1	B1
40	2RCS	Antibody			

Binding Surface class S2 (with a pocket binding site on the antibody-free structure, but not on the antibody-antigen complex) is associated with B3 (with CDR changes only). In addition, none of class B3 have shown binding surface class S1 (pockets on the antibody-antigen complex, but no pocket on the free antibody form). Therefore, these results could suggest that large CDR movements are associated with the closure of the pockets in the antibody-antigen complexes, and not involved in the opening of these pockets if the antibody-free structure displays a non-pocket binding site.

The presence of pockets in the antibody-antigen complexes, but not in the free form (S1) or the presence of pockets on both the free and bound forms (S3) were associated with either classes B1 or B4 (random movements) or class B2 (C_Loop1 specific movements). Consequently, the presence of a pocket in the binding sites of the antibody-bound form could be correlated to movements in C_Loop1 and/or with high movements in the entire antibody. However, the high movement of C_Loop1, and the generation of these pockets in the antibody-bound form could also be a result of an induced-fit mechanism or by binding to one of the pre-existing free conformations.

Taken together, these data illustrate the complexity of the antibody-antigen interaction process that could be restricted to the binding sites, or extended through the constant domains to propagate structural changes beyond these sites. In order to further illustrate these concepts, we now examine and discuss one specific example in detail.

### Specific Analysis of Anti-Cocaine Antibody

We analyzed the antibody 7A1 and compared it to its set of antigen-bound forms. This antibody hydrolyses cocaine to produce the non-psychoactive metabolites ecgonine methyl ester and benzoic acid. Crystal structures of 7A1 Fab (2AJU) and six complexes with substrate cocaine (2AJV), the transition state analogue (2AJX), the products ecgonine methyl ester and benzoic acid together (2AJY) and individually (2AJZ and 2AK1), as well as with heptaethylene glycol (2AJS), have all been resolved by Zhu et al. (2006) and are all available from the PDB. These authors identified significant structural rearrangements that occur along the reaction pathway, which they proposed to be limited to the binding site (Zhu et al., 2006). This suggestion was based on identifying modest CDR loop movements (up to 2.3 Å), along with substantial side chain rearrangements (up to 9 Å) that have altered the shape and size (∼320–500 Å^3^) of the antibody active site from open (for the substrate) to closed (transition state) to open (product states) (Zhu et al., 2006).

**FIGURE 4:**
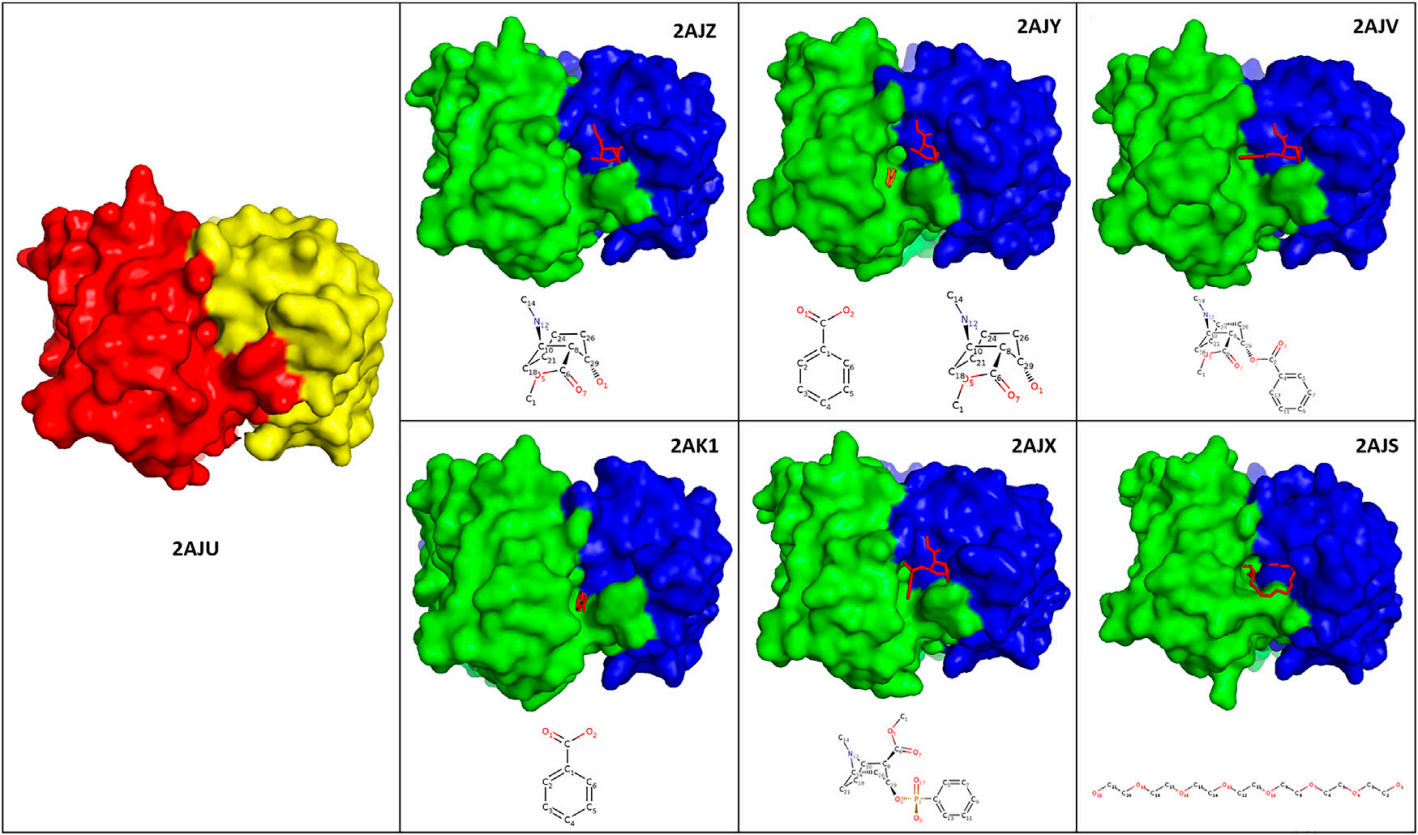
Binding sites of antibody 7A1. Illustration of the crystal structures of antibody 7A1 as free form (2AJU) and six antigen bound states against Ecgonine methyl ester (2AJZ), Ecgonine methyl ester and benzoic acid (2AJY), Cocaine (2AJV), Benzoic acid (2AK1), 3-(hydroxy-phenyl-phosphinoyloxy)-8-methyl-8-aza-bicyclo[3.2.1]octane-2-carboxylic acid methyl ester (2AJX), and PEG330 (2AJS). The heavy and light chains of the antibody-free form of 2AJU were colored as red and yellow, respectively. Whilst green (heavy chains) and blue (light chains) was used to color the antigen bound structures. Chemical structure of each of the antigens was depicted at the bottom of each crystal structure.

Antibody 7A1 is IgG2a-Kappa, and the binding sites transition from open (2AJV) to closed (2AJX) to open (2AJZ, 2AJY, and 2AK1) can be observed in Figure 4. This closure can be attributed to the twisting of the benzene ring of the transitional state antigen of 2AJX when compared to cocaine in 2AJV. The cocaine bound crystal structure (2AJU) also shows closure of a very small binding pocket in the center of the binding sites of the remaining free and bound crystal structures (Figure 4). Zhu et al. have crystalized antibody 7A1 in different antigen-bound forms, and comprehensively reported these atomistic interactions (Zhu et al., 2006).

RMSD comparison of the bound vs. free forms has confirmed suggestions by Zhu et al. that the VH and VL domains have moved more than the C_H1_ and C_L_ counterparts. In addition, the light chains of 2AJY, 2AK1, and 2AJV have moved more than the heavy chains (Supplementary Table S1.2). Therefore, this movement could be attributed to the binding of ecgonine methyl ester to the light chains, and the benzene ring to the heavy chain, which cause further light chains movements.

**FIGURE 5:**
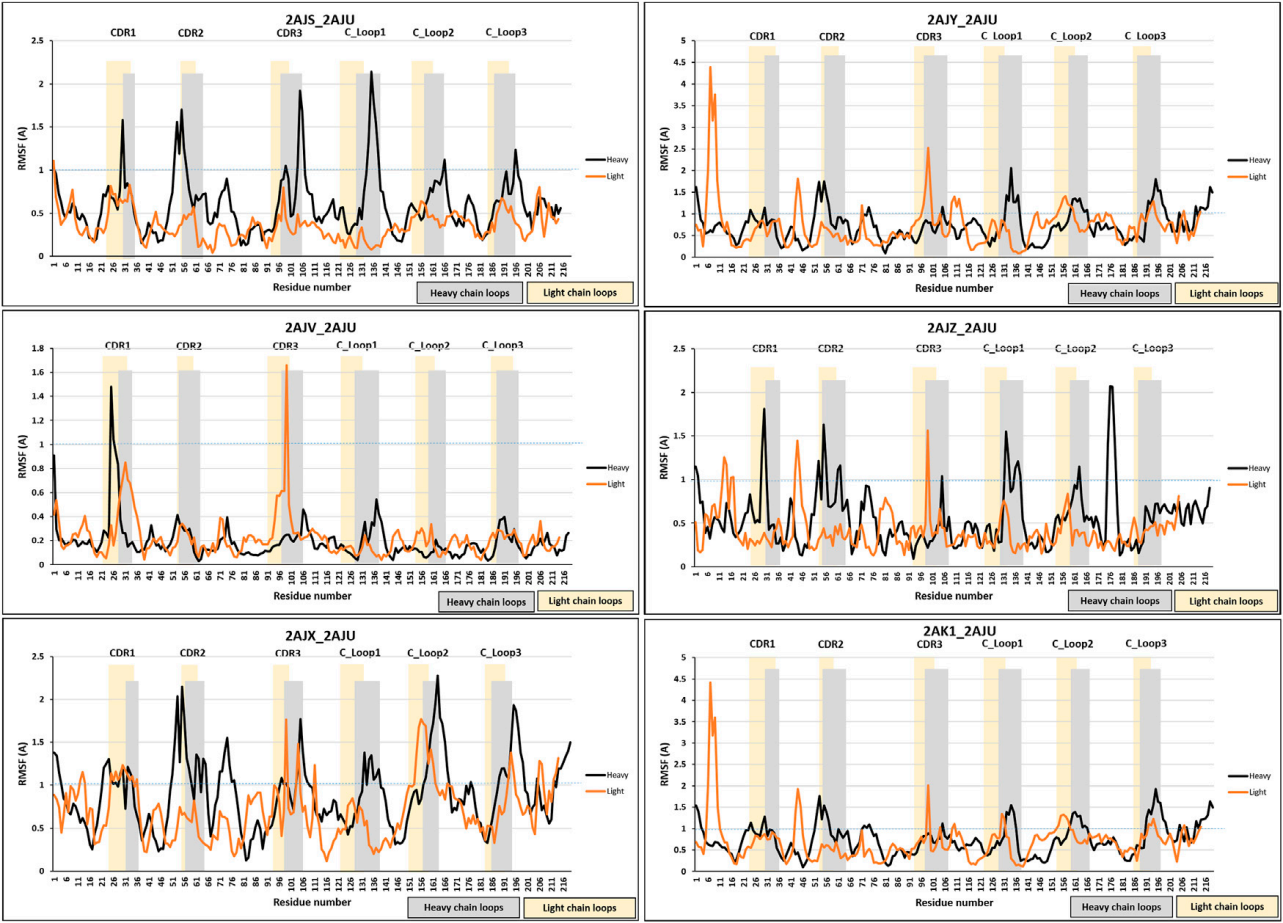
RMSF analysis of antibody 7A1. RMSF measurements were analyzed for the six bound states of antibody 7A1. Blue dotted line was placed at RMSF 1 Å to allow easier comparison. The bound states were 2AJS, 2AJY, 2AJV, D 2AJZ, 2AJX, and 2AK1.

Despite the high movement of the variable domains (V_H_ and V_L_), the RMSF analyses have also shown additional movement of the constant domains (C_H1_ and C_L_) (Figure 5). 2AJV has shown specific movement of CDRL1, CDRL3 and an area next to CDRH1 to accommodate cocaine through an open binding state (Figure 5). Whilst in the transition state of 2AJX, the active site demonstrates a closed form, in which the CDRH2 progress toward the active site (Figure 5 and Supplementary Figure S.2.1.13). The remaining five antigen-bound states have demonstrated general fluctuation with greater focus on the loops (Figure 5). An important region for structural analysis is C_Loop1 of the heavy chain. The movement of this loop was between 1 and 2 Å in all bound states except the main anti-cocaine antibody 2AJV (∼0.55 Å). This reduced movement of C_Loop1 in 2AJV could be correlated with the disappearance of the small pocket at the center of the groove binding site (Figure 4). We note that the scale of the C_Loop1 changes is much lower than found in classes B1 and B2 where clear evidence of signal propagation is found. Indeed, all these crystal structures have shown class B3 structural changes, and S3 surfaces apart from 2AJV (class S2), which is the crystal structure of the cocaine bound antibody. These structural changes match the function of this antibody in hydrolyzing cocaine rather than propagation of an immune response.

## Conclusion

In this paper we have extended our analysis of Fab structural changes induced by antigen binding from proteins (Al Qaraghuli et al., 2020) to haptens. Crystal structures of anti-hapten antibodies were retrieved from the PDB, and comprehensive analyses were conducted to examine their binding sites and the structural changes caused by antigen binding.

We have identified three classifications of binding surface conformational changes induced by hapten interactions:

S1: The hapten binding causes the creation of a pocket on the Fab binding surface, enabled by the movement of the entire chains.
S2: The hapten binding removes a pocket from the Fab binding surface, through changing the CDR conformation.
S3: The binding surface remains pocket-free before and after hapten binding.

Classifications S1 and S2 are to be expected with an induced-fit picture of how antibodies bind to hapten antigens. The prevalence of S3 indicates that this picture is not complete, and that some Fabs can exist in high-affinity conformations to bind their hapten targets. The latter picture is reminiscent of the affinity shown by antibodies to much larger proteins with larger binding interfaces.

We also analyzed structural changes across the whole Fab caused by the hapten antigen binding, and four different classifications of behavior were identified:

B1: The hapten binding causes deformation of the diamond-like Fab structure as well as prominent changes in the C_Loop_1_ region indicating a potential allosteric signal propagation.
B2: The hapten binding does not deform the entire Fab but does still induce changes at the C_Loop_1_.
B3: The effects of the hapten binding are restricted to local changes to the CDRs.
B4: Only minor structural changes throughout the Fab are caused by the hapten binding.

These classifications align well with those we found for protein-binding Fabs (Al Qaraghuli et al., 2020) lending weight to our interpretation that potential structural changes must be specifically addressed in the development of novel therapeutics. Our focus on the cocaine-hydrolyzing antibody revealed it to be class B3, which can be understood in that the functionality does not require structural changes to affect further immunological responses. Finally, class B4 is new to the hapten antigens, but perhaps is not unexpected if the binding is relatively weak. We have found that there is some weak correlation between the binding surface classification S2 (where a pocket at the Fab binding surface is removed upon hapten binding) and the absence of potential structural changes in B3.

From this study, we recommend a careful analysis of the entire antibody structure, and not just the binding surfaces in isolation, and correlate them to structural changes throughout the antibody. This will undoubtedly enable the identification of long-range structural changes that have the potential to induce an Fc effector response. This study could be extended in the future by utilizing molecular dynamics simulation to analyze the movements of a sample antibody to identify such long-range movements.

## Data Availability

The original contributions presented in the study are included in the article/Supplementary Material, further inquiries can be directed to the corresponding author.
