# Supplementary material for: Structural Analysis of Anti-Hapten Antibodies to Identify Long-Range Structural Movements Induced by Hapten Binding
Source: Front Mol Biosci. 2021 Mar 24;8:633526. doi: 10.3389/fmolb.2021.633526 (PMC8044860; doi:10.3389/fmolb.2021.633526)
Supplement: Supplementary file 1 [file datasheet1.pdf]

Structural analysis of anti-hapten antibodies to identify long-range structural movements induced by hapten binding

Mohammed M. Al Qaraghuli[1,2,*], Karina Kubiak-Ossowska[1,3], Valerie A. Ferro[4], and Paul A. Mulheran[1]

1 Department of Chemical and Process Engineering, University of Strathclyde, Glasgow, UK, G1 1XJ.

2 SiMologics Ltd. The Enterprise Hub, Level 6 Graham Hills Building, 50 Richmond Street, Glasgow, UK, G1 1XP.

3 Department of Physics, University of Strathclyde, Glasgow, UK, G4 0NG

4 Strathclyde Institute of Pharmacy and Biomedical Sciences, University of Strathclyde, 161 Cathedral Street, Glasgow, UK, G4 0RE.

* Correspondence: Email: mohammed.al-qaraghuli@strath.ac.uk; Tel.: +44 (0)141 548 2176

Supplementary Data

Supplementary S1: Structures selection and classification 4
Table S1.1: Details of selected crystal structures 4
Table S1.2: RMSD measurements 10
Supplementary S2: Antibodies structural analyses 14
S.2.1 (3CFB vs 3CFC), mouse: 15
S.2.2 (3CFD vs 3CFE), mouse: 17
S.2.3 (1UB5 vs 1UB6), mouse: 20
S.2.4 (1Q72 vs 1RFD), mouse: 22
S.2.5 (1QYG vs 1RFD), mouse: 25
S.2.6 (1JNN vs 1JNL), mouse: 27
S.2.7 (1KEL vs 1KEM), mouse: 29
S.2.8 (1NGP vs 1NGQ), mouse: 31
S.2.9 (2CGR vs 1CGS), mouse: 33
S.2.10 (1Q0Y vs 1Q0X), mouse: 35
S.2.11 (2AJS vs 2AJU), mouse: 37
S.2.12 (2AJV vs 2AJU), mouse: 39
S.2.13 (2AJX vs 2AJU), mouse: 41
S.2.14 (2AJY vs 2AJU), mouse: 43
S.2.15 (2AJZ vs 2AJU), mouse: 45
S.2.16 (2AK1 vs 2AJU), mouse: 47

S.2.17 (4OCX vs 4OCY), mouse: ... 49
S.2.18 (1I9J vs 1I9I), mouse: ... 52
S.2.19 (3LS4 vs 3LS5), mouse: ... 54
S.2.20 (1FL6 vs 1FL5), human: ... 56
S.2.21 (1C5C vs 1C5B), human: ... 58
S.2.22 (1D6V vs 1D5B), human: ... 60
S.2.23 (1AJ7 vs 2RCS), human: ... 62
Supplementary S3: Sequences Alignment ... 64
Table S3.1 Hapten_Human_Heavy chain_8 sequences ... 65
Table S3.2 Hapten_Human_Light chain_Kappa_8 sequences ... 66
Table S3.3 Hapten_Mouse_Heavy chain_32 sequences ... 67
Table S3.4 Hapten_Mouse_Light chain_Kappa_28 sequences ... 69
Table S3.5 Hapten_Mouse_Light chain_Lambda_4 sequences ... 71
Supplementary S4: Angles and distances ... 72

Supplementary S1: Structures selection and classification

Table S1.1: Details of selected crystal structures

	PDB ID	Source (format)	Crystal structure description	Target (Molecular weight-Da)	Crystal form	Clone name (heavy/light)	Genetic class	References
1	3CFB	Mus musculus (Fab)	High-resolution structure of blue fluorescent antibody EP2-19G2 in complex with stilbene hapten at 100K	4-(4-styryl-phenylcarbamoyl)-butyric acid (309.36)	Antigen-antibody	EP2-19G2	(IgG2b-Kappa)	(Debler et al. 2008)
2	3CFC	Mus musculus (Fab)	High-resolution structure of blue fluorescent antibody EP2-19G2	--	Antibody	EP2-19G2	(IgG2b-Kappa)	(Debler et al. 2008)
3	3CFD	Mus musculus (Fab)	Purple-fluorescent antibody EP2-25C10 in complex with its stilbene hapten	4-(4-styryl-phenylcarbamoyl)-butyric acid (309.36)	Antigen-antibody	EP2-25C10	(IgG2b-Kappa)	(Debler et al. 2008)
4	3CFE	Mus musculus (Fab)	Crystal structure of purple-fluorescent antibody EP2-25C10	--	Antibody	EP2-25C10	(IgG2b-Kappa)	(Debler et al. 2008)
5	1UB5	Mus musculus (Fab)	Crystal structure of Antibody 19G2 with hapten at 100K	4-(4-styryl-phenylcarbamoyl)-butyric acid (309.36)	Antigen-antibody	19G2	(IgG2b-Kappa)	--
6	1UB6	Mus musculus (Fab)	Crystal structure of Antibody 19G2 with sera ligand	--	Antibody	19G2	(IgG2b-Kappa)	--

7	1Q72	Mus musculus (Fab)	Anti-Cocaine Antibody M82G2 Complexed with Cocaine	Cocaine (303.35)	Antigen-antibody	M82G2	(IgG1-Kappa)	(Pozharski et al. 2005)
8	1QYG	Mus musculus (Fab)	Anti-cocaine antibody M82G2 complexed with benzoylecgonine	Benzoylecgonine (289.33)	Antigen-antibody	M82G2	(IgG1-Kappa)	(Pozharski et al. 2005)
9	1RFD	Mus musculus (Fab)	Anti-cocaine antibody M82G2	--	Antibody	M82G2	(IgG1-Kappa)	(Pozharski et al. 2005)
10	1JNN	Mus musculus (Fab)	Crystal Structure of Fab-Estradiol Complexes	Estradiol (272.38)	Antigen-antibody	17E12E5	(IgG1-Kappa)	(Monnet et al. 2002)
11	1JNL	Mus musculus (Fab)	Crystal Structure of Fab-Estradiol Complexes	--	Antibody	17E12E5	(IgG1-Kappa)	(Monnet et al. 2002)
12	1KEL	Mus musculus (Fab)	Catalytic antibody 28b4 fab fragment complexed with hapten (1-[n-4'-nitrobenzyl-n-4'-carboxybutylamino] methylphosphonic acid)	1-[N-4'-Nitrobenzyl-n-4'-carboxybutylamino] methylphosphonic acid (346.27)	Antigen-antibody	28B4	(IgG1-Kappa)	(Hsieh-Wilson, Schultz, and Stevens 1996)
13	1KEM	Mus musculus (Fab)	Catalytic antibody 28b4 fab fragment	--	Antibody	28B4	(IgG1-Kappa)	(Hsieh-Wilson, Schultz, and Stevens 1996)
14	1NGP	Mus musculus (Fab)	NLG9 (IgG1-lambda) Fab fragment complexed with (4-hydroxy-3-nitrophenyl) acetate	(4-Hydroxy-3-nitrophenyl) acetate (197.14)	Antigen-antibody	N1G9	(IgG1-Lambda)	(Mizutani et al. 1995)
15	1NGQ	Mus musculus (Fab)	NLG9 (IgG1-lambda) Fab fragment	--	Antibody	N1G9	(IgG1-Lambda)	(Mizutani et al. 1995)

16	2CGR	Mus musculus (Fab)	Local and transmitted conformational changes on complexation of an anti-sweetener fab	N-(p-cyanophenyl)-n'-diphenylmethyl-guanidine- acetic acid (384.43)	Antigen-antibody	NC6.8	(IgG2b-Kappa)	(Guddat et al. 1994)
17	1CGS	Mus musculus (Fab)	Local and transmitted conformational changes on complexation of an anti-sweetener fab	--	Antibody	NC6.8	(IgG2b-Kappa)	(Guddat et al. 1994)
18	1Q0Y	Mus musculus (Fab)	Anti-Morphine Antibody 9B1 Complexed with Morphine	morphine (285.34)	Antigen-antibody	9B1	(IgG-Lambda)	(Pozharski et al. 2004)
19	1Q0X	Mus musculus (Fab)	Anti-morphine Antibody 9B1 Unliganded Form	--	Antibody	9B1	(IgG-Lambda)	(Pozharski et al. 2004)
20	2AJS	Mus musculus (Fab)	Crystal structure of cocaine catalytic antibody 7A1 Fab' in complex with heptaethylene glycol	PEG330 (326.38)	Antigen-antibody	7A1	(IgG2a-Kappa)	(Zhu et al. 2006)
21	2AJV	Mus musculus (Fab)	Crystal Structure of Cocaine catalytic Antibody 7A1 Fab' in Complex with Cocaine	Cocaine (303.35)	Antigen-antibody	7A1	(IgG2a-Kappa)	(Zhu et al. 2006)
22	2AJX	Mus musculus (Fab)	Crystal Structure of Cocaine catalytic Antibody 7A1 Fab' in Complex with Transition State Analog	3-(hydroxy-phenyl-phosphinoyloxy)-8-methyl- 8-aza-bicyclo[3.2.1]octane-2-carboxylic acid methyl ester (339.32)	Antigen-antibody	7A1	(IgG2a-Kappa)	(Zhu et al. 2006)

23	2AJY	Mus musculus (Fab)	Crystal Structure of Cocaine catalytic Antibody 7A1 Fab' in Complex with ecgonine methyl ester and benzoic acid	Ecgonine methyl ester (199.25) and benzoic acid (122.12)	Antigen-antibody	7A1	(IgG2a-Kappa)	(Zhu et al. 2006)
24	2AJZ	Mus musculus (Fab)	Crystal Structure of Cocaine catalytic Antibody 7A1 Fab' in Complex with ecgonine methyl ester	Ecgonine methyl ester (199.25)	Antigen-antibody	7A1	(IgG2a-Kappa)	(Zhu et al. 2006)
25	2AK1	Mus musculus (Fab)	Crystal Structure of Cocaine catalytic Antibody 7A1 Fab' in Complex with benzoic acid	Benzoic acid (122.12)	Antigen-antibody	7A1	(IgG2a-Kappa)	(Zhu et al. 2006)
26	2AJU	Mus musculus (Fab)	Cyrstal structure of cocaine catalytic antibody 7A1 Fab'	--	Antibody	7A1	(IgG2a-Kappa)	(Zhu et al. 2006)
27	4OCX	Mus musculus (Fab)	Fab complex with methotrexate	Methotrexate protonated at N1 (455.45)	Antigen-antibody	ADD056	(IgG2a – Kappa)	(Gayda et al. 2014)
28	4OCY	Mus musculus (Fab)	Fab for methotrexate (unbound apo)	--	Antibody	ADD056	(IgG2a – Kappa)	(Gayda et al. 2014)
29	1I9J	Mus musculus (Fab)	Testosterone complex structure of the recombinant monoclonal wild type anti-testosterone fab fragment	Testosterone (288.42)	Antigen-antibody		(IgG2b-Kappa)	(Valjakka et al. 2002)
30	1I9I	Mus musculus (Fab)	Native crystal structure of the recombinant monoclonal wild type anti-testosterone fab fragment	--	Antibody		(IgG2b-Kappa)	(Valjakka et al. 2002)

31	3LS4	Mus musculus (Fab)	Crystal Structure of Anti-tetrahydrocannabinol Fab Fragment in Complex with THC	Tetrahydrocannabinol (314.46)	Antigen-antibody	T3	(IgG1-Kappa)	(Niemi et al. 2010)
32	3LS5	Mus musculus (Fab)	Anti-tetrahydrocannabinol Fab Fragment, Free Form	--	Antibody	T3	(IgG1-Kappa)	(Niemi et al. 2010)
33	1FL6	Chimeric (Mus musculus/Homo sapiens) (Fab)	The hapten complexed germline precursor to sulfide oxidase catalytic antibody 28b4	1-[n-4'-nitrobenzyl-n-4'-carboxybutylamino]methylphosphonic acid (346.27)	Antigen-antibody	28B4	(IgG1-Kappa)	(Yin et al. 2001)
34	1FL5	Chimeric (Mus musculus/Homo sapiens) (Fab)	The unliganded germline precursor to the sulfide oxidase catalytic antibody 28b4.	--	Antibody	28B4	(IgG1-Kappa)	(Yin et al. 2001)
35	1C5C	Chimeric (Mus musculus/Homo sapiens) (Fab)	Decarboxylase catalytic antibody 21d8-hapten complex	2-acetylamino-napthalene-1,5-disulfonic acid (345.35)	Antigen-antibody	21D8	(IgG1-Kappa)	(Hotta et al. 2000)
36	1C5B	Chimeric (Mus musculus/Homo sapiens) (Fab)	Decarboxylase catalytic antibody 21d8 unliganded form	--	Antibody	21D8	(IgG1-Kappa)	(Hotta et al. 2000)
37	1D6V	Homo sapiens (Fab)	Conformation effects in biological catalysis introduced by oxy-cope antibody maturation	Dansyl (381.46)	Antigen-antibody	AZ-28	(IgG1-Kappa)	(Mundorff et al. 2000)
38	1D5B	Homo sapiens (Fab)	Unliganded mature oxy-cope catalytic antibody	--	Antibody	AZ-28	(IgG1-Kappa)	(Mundorff et al. 2000)

39	1AJ7	Homo sapiens (Fab)	Immunoglobulin 48g7 germline fab antibody complexed with hapten 5-(para-nitrophenyl phosphonate)-pentanoic acid. affinity maturation of an esterolytic antibody	5-(Para-nitrophenyl phosphonate)-pentanoic acid (302.2)	Antigen-antibody	48G7	(IgG1-Kappa)	(Wedemayer et al. 1997)
40	2RCS	Homo sapiens (Fab)	Immunoglobulin 48g7 germline fab-affinity maturation of an esterolytic antibody	--	Antibody	48G7	(IgG1-Kappa)	(Wedemayer et al. 1997)

Table S1.2: RMSD measurements

PDB number	PDB ID	Resolution (Å)	PDB couples	Constant (resid 114-218)	Variable (resid 1-105)	Linker (resid 106-113)	Total RMSD (VMD)	RMSA trend (domain)	RMSD trend (chain)
1	3CFB	1.6	3cfbL vs 3cfcL	0.34	0.56	0.39	1.55	VL more	Light less
	3CFC	1.7	3cfbH vs 3cfcH	1.11	0.48	0.43	2.04	CH more	
2	3CFD	2.5	3cfdL vs 3cfeL	0.2	0.21	0.13	0.33	VL more	Light more
	3CFE	2.99	3cfdH vs 3cfeH	0.24	0.22	0.14	0.31	CH more	
3	1UB5	2	1ub5L vs 1ub6L	3.72	3.67	1.85	3.69	CL more	Light less
	1UB6	2.12	1ub5H vs 1ubbH	3.66	3.72	2.27	3.7	VH more	
4	1Q72	1.7	1q72L vs 1rfdL	0.21	0.14	0.07	0.27	CL more	Light less
	1QYG	1.81	1q72H vs 1rfdH	0.6	0.48	0.1	0.54	CH more	
5	1RFD	2.09	1qygL vs 1rfdL	0.19	0.14	0.09	0.23	CL more	Light less

			1qygH ve 1rfdH	0.65	0.56	0.15	0.64	CH more	
6	1JNN	3.2	1jnnL vs 1jnlL	0.52	0.35	0.2	0.51	CL more	Light more
	1JNL	3	1jnnH vs 1jnlH	0.48	0.42	0.33	0.47	CH more	
7	1KEL	1.9	1kelL vs 1kemL	0.29	0.46	0.23	0.44	VL more	Light more
	1KEM	2.2	1kelH vs 1kemH	1.44	0.44	0.21	1.14	CH more	
8	1NGP	2.4	1ngpL vd 1ngqL	0.33	0.21	0.14	0.31	CL more	Light less
	1NGQ	2.4	1ngpH vs 1ngqH	0.38	0.33	0.16	0.41	CH more	
9	2CGR	2.2	2cgrL vs 1cgsL	0.9	0.77	1.4	3.64	CL more	Light less
	1CGS	2.6	2cgrH vs 1cgsH	1.27	0.66	0.37	3.88	CH more	
10	1Q0Y	2	1q0yL vs 1q0xL	0.13	0.22	0.09	0.28	VL more	Light less
	1Q0X	1.6	1q0yH vs 1q0xH	0.23	0.21	0.07	0.34	CH more	
11	2AJS	1.7	2ajsL vs 2ajuL	0.26	0.26	0.09	0.41	SAME	Light less

	2AJV	1.5	2ajsH vs 2ajuH	0.41	0.46	0.37	0.69	VH more	
12	2AJX	1.85	2ajxL vs 2ajuL	0.3	0.42	0.35	0.79	VL more	Light less
	2AJY	2.1	2ajxH vs 2ajuH	0.37	0.52	0.24	0.98	VH more	
13	2AJZ	2.3	2ajyL vs 2ajuL	0.35	0.82	0.28	0.9	VL more	Light more
	2AK1	1.85	2ajyH vs 2ajuH	0.44	0.48	0.25	0.82	VH more	
14	2AJU	1.5	2ak1L vs 2ajuL	0.34	0.77	0.21	0.86	VL more	Light more
			2ak1H vs 2ajuH	0.36	0.45	0.22	0.83	VH more	
15			2ajvL vs 2ajuL	0.2	0.28	0.05	0.29	VL more	Light more
			2ajvH vs 2ajuH	0.13	0.28	0.15	0.25	VH more	
16			2ajzL vs 2ajuL	0.29	0.48	0.28	0.46	VL more	Light less
			2ajzH vs 2ajuH	0.51	0.53	0.21	0.65	VH more	
17	4OCX	2.39	4ocxL vs 4ocyL	1	0.28	0.23	0.79	CL more	Light more

	4OCY	2.79	4ocxH vs 4ocyH	0.41	0.23	0.29	0.47	CH more	
18	1I9J	2.6	1i9jL vs 1i9iL	0.58	0.57	0.25	0.59	CL more	Light less
	1I9I	2.72	1i9jH vs 1i9iH	0.73	0.42	0.11	0.65	CH more	
19	3LS4	2	3ls4L vs 3ls5L	0.32	0.26	0.11	0.35	CL more	Light less
	3LS5	1.9	3ls4H vs 3ls5H	0.43	0.22	0.09	0.44	CH more	
20	1FL6	2.8	1fl6L vs 1fl5L	1.06	0.71	0.37	1.2	CL more	Light less
	1FL5	2.1	1fl6H vs 1fl5H	1.77	0.68	0.22	2.04	CH more	
21	1C5C	1.61	1c5cL vs 1c5bL	0.71	0.36	0.16	0.57	CL more	Light less
	1C5B	2.1	1c5cH vs 1c5bH	0.93	0.32	0.21	0.75	CH more	
22	1D6V	2	1d6vL vs 1d5bL	0.77	0.62	0.27	3.25	CL more	Light less
	1D5B	2.8	1d6vH vs 1d5bH	2.41	0.92	0.44	5.02	CH more	
23	1AJ7	2.1	1aj7L vs 2rcsL	0.88	0.48	0.24	6.02	CL more	Light less
	2RCS	2.1	1aj7H vs 2rcsH	1.7	0.81	0.16	7.21	CH more	

Supplementary S2: Antibodies structural analyses

For each couple, two crystal structures were compared: one as free antibody and the second as antibody/antigen complex. The selected couples were of human (4) and mouse (19) origin. The analyses included A) Comparison of the binding sites surfaces between a free antibody and antibody/antigen crystal structures. B) Orientation of the six CDRs of the two crystal structures. C) Alignment of the two crystal structures to compare deviation in the loops. D) Alignment of the CDRs along with their amino acid positions, which were numbered sequentially. E) RMSF comparison of each amino acid position in the two compared crystal structures. This comparison was performed on the total chain (heavy and light) and independently on their specific domains (VH, CH, VL, and CL). Heavy chain loops were highlighted in grey and light chain loops were highlighted in yellow. The three loops in the variable domains were named CDR1-3, and the three loops in the constant domains (CH and CL) were named C_Loop1-3, according to their location throughout the sequence from the N-terminal to the C-terminal of the entire chain.

S.2.1 (3CFB vs 3CFC), mouse:

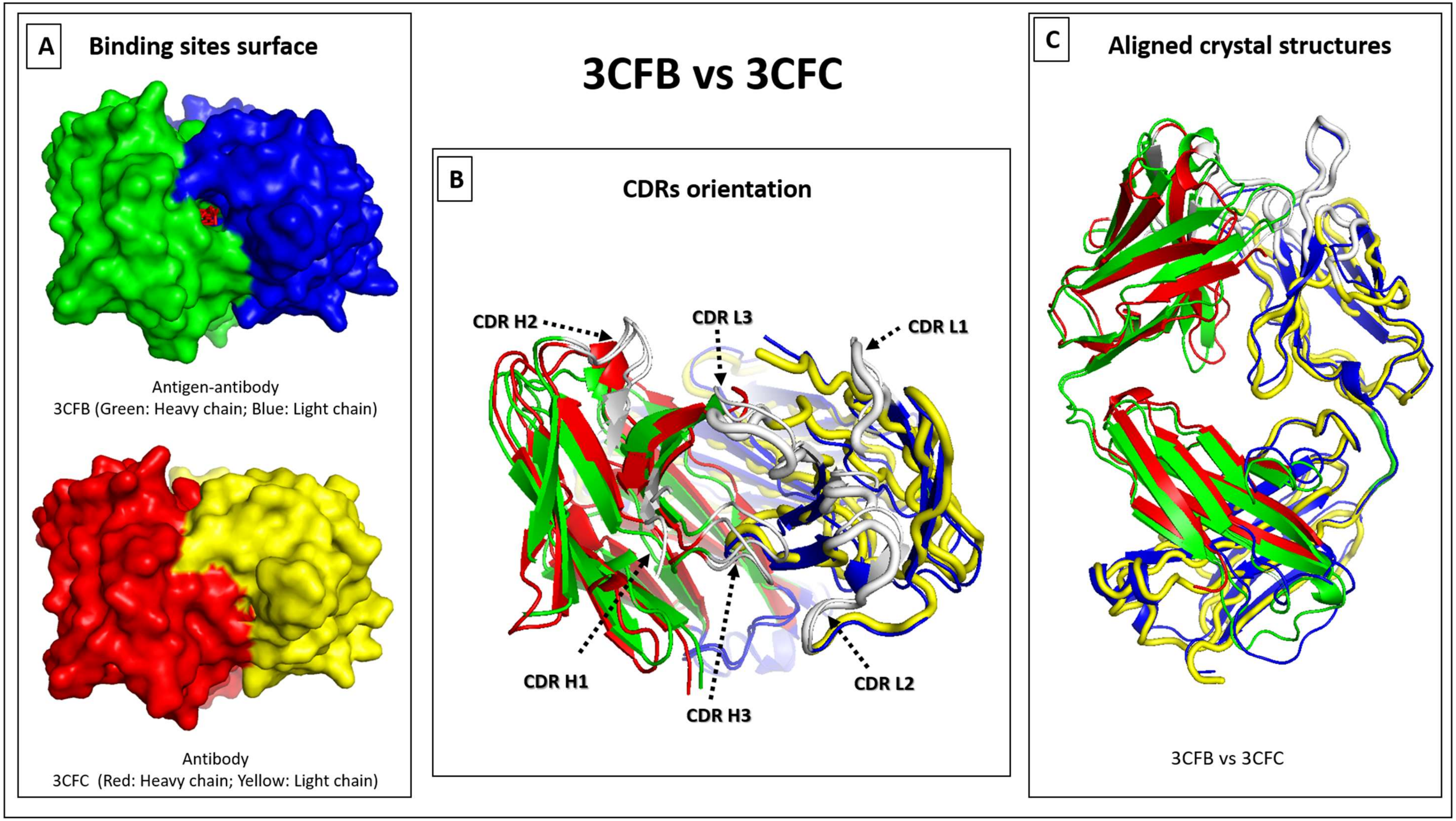

3CFB vs 3CFC

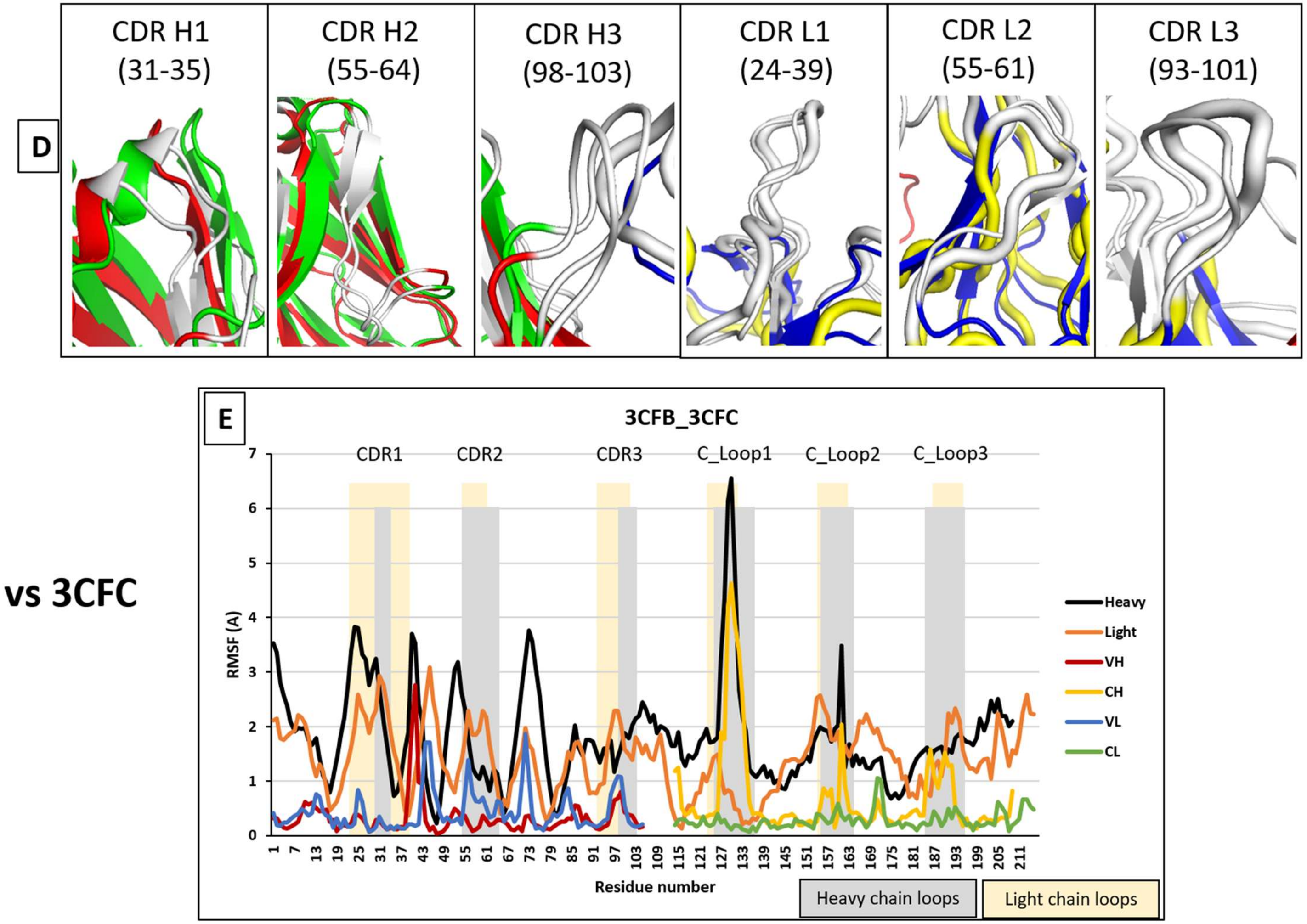
D
CDR H1 (31-35)
CDR H2 (55-64)
CDR H3 (98-103)
CDR L1 (24-39)
CDR L2 (55-61)
CDR L3 (93-101)
E
3CFB_3CFC
CDR1
CDR2
CDR3
C_Loop1
C_Loop2
C_Loop3
RMSF (A)
Residue number
Heavy
Light
VH
CH
VL
CL
Heavy chain loops
Light chain loops

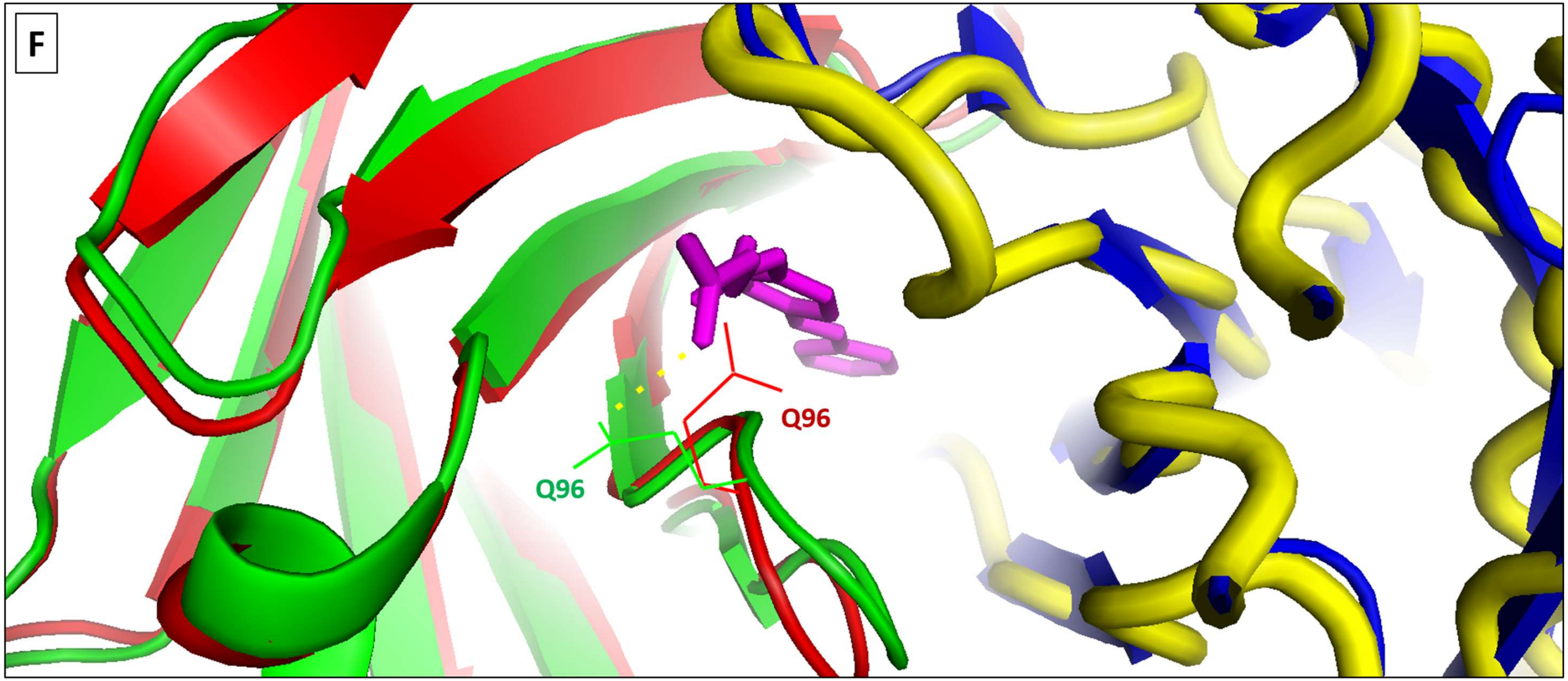
F
Q96
Q96

S.2.2 (3CFD vs 3CFE), mouse:

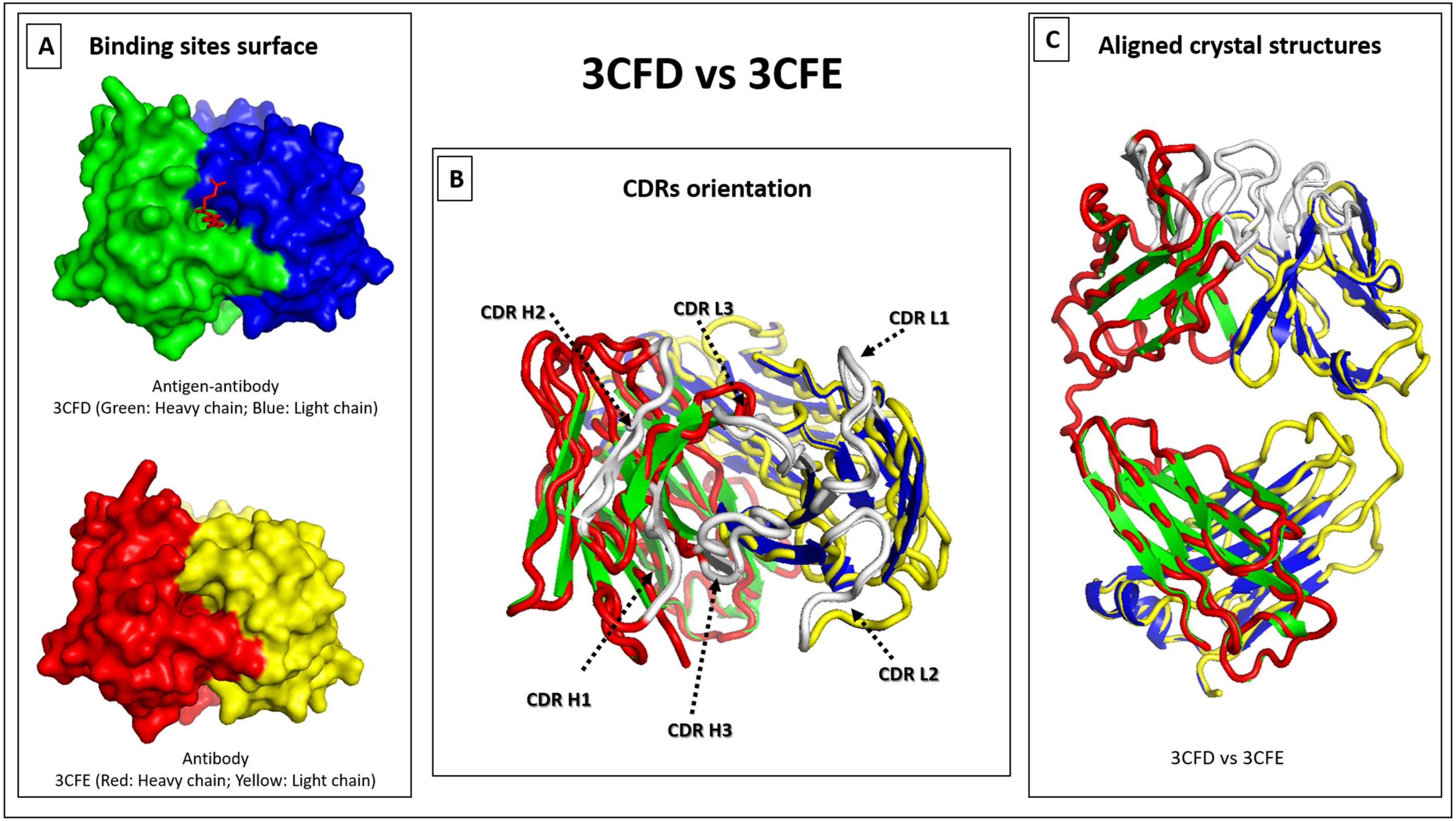

3CFD vs 3CFE

D

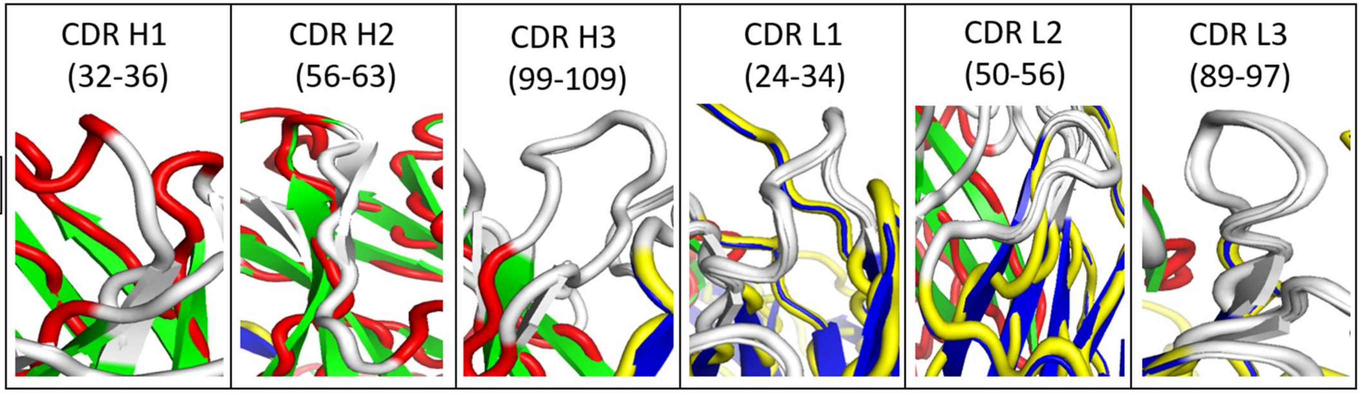

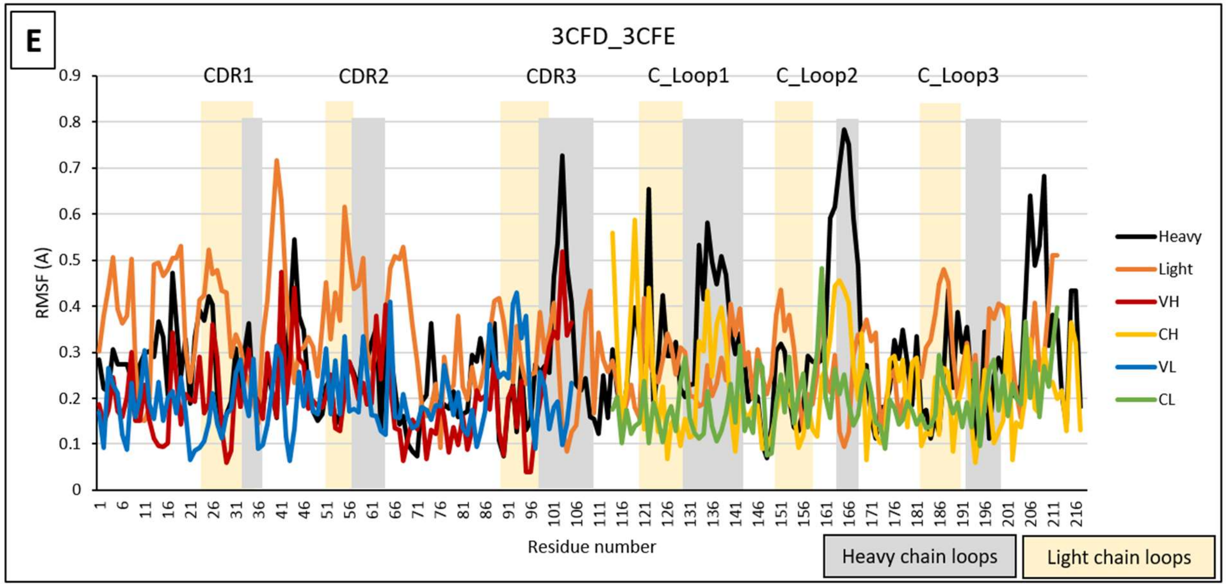

S.2.3 (1UB5 vs 1UB6), mouse:

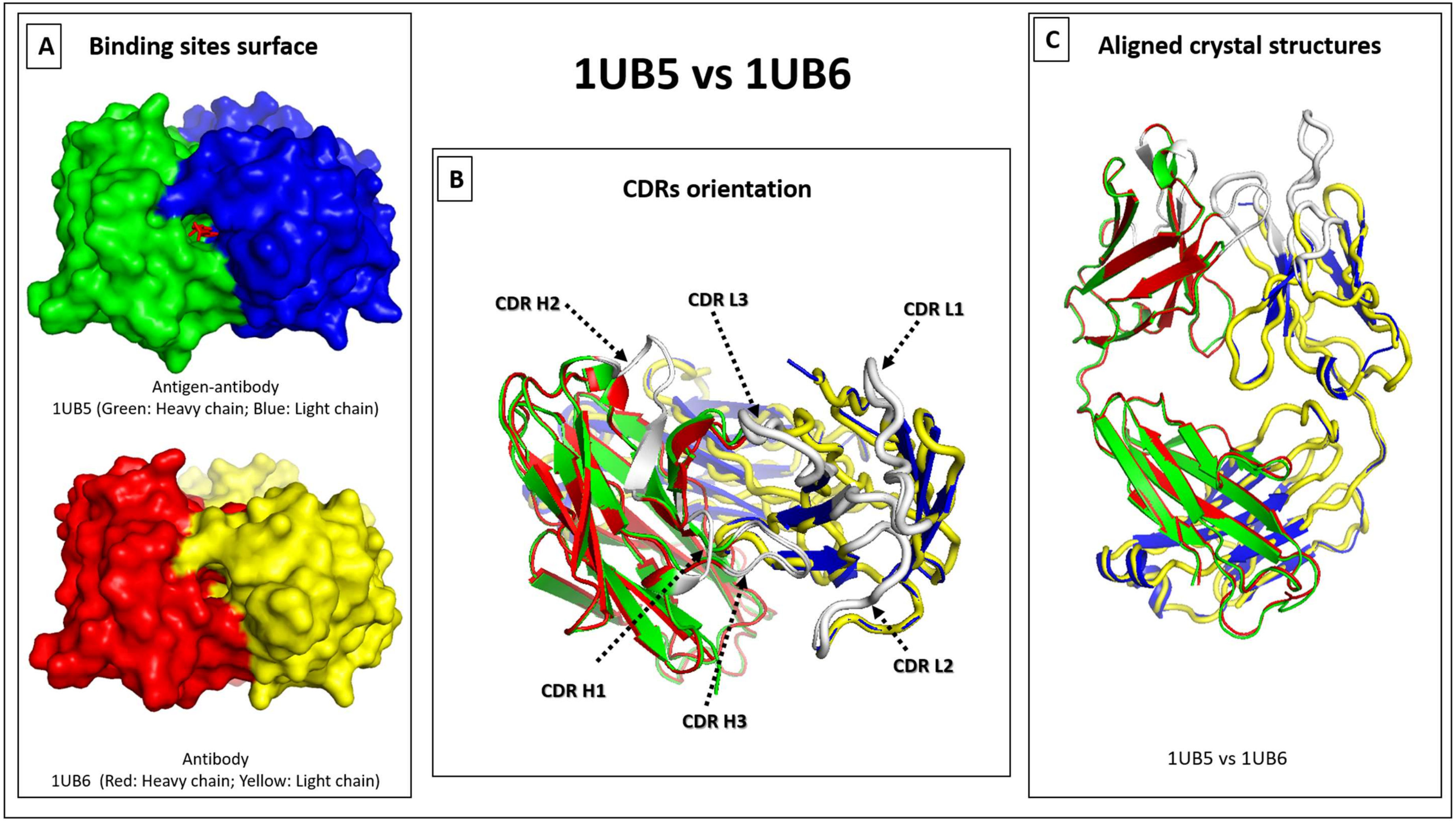

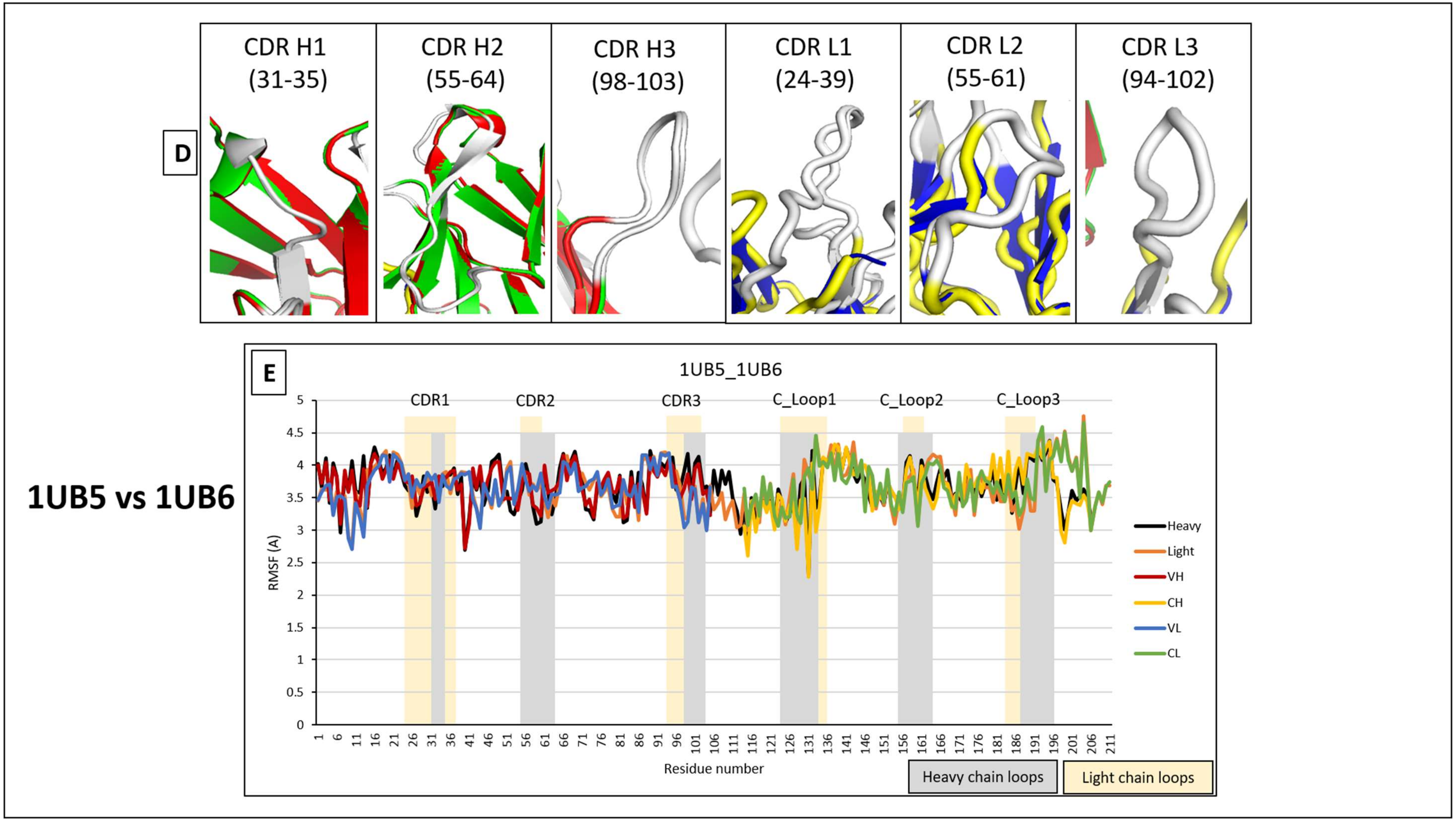

D
CDR H1
(31-35)
CDR H2
(55-64)
CDR H3
(98-103)
CDR L1
(24-39)
CDR L2
(55-61)
CDR L3
(94-102)
1UB5 vs 1UB6
E
1UB5_1UB6
CDR1
CDR2
CDR3
C_Loop1
C_Loop2
C_Loop3
RMSF (A)
Residue number
Heavy
Light
VH
CH
VL
CL
Heavy chain loops
Light chain loops

S.2.4 (1Q72 vs 1RFD), mouse:

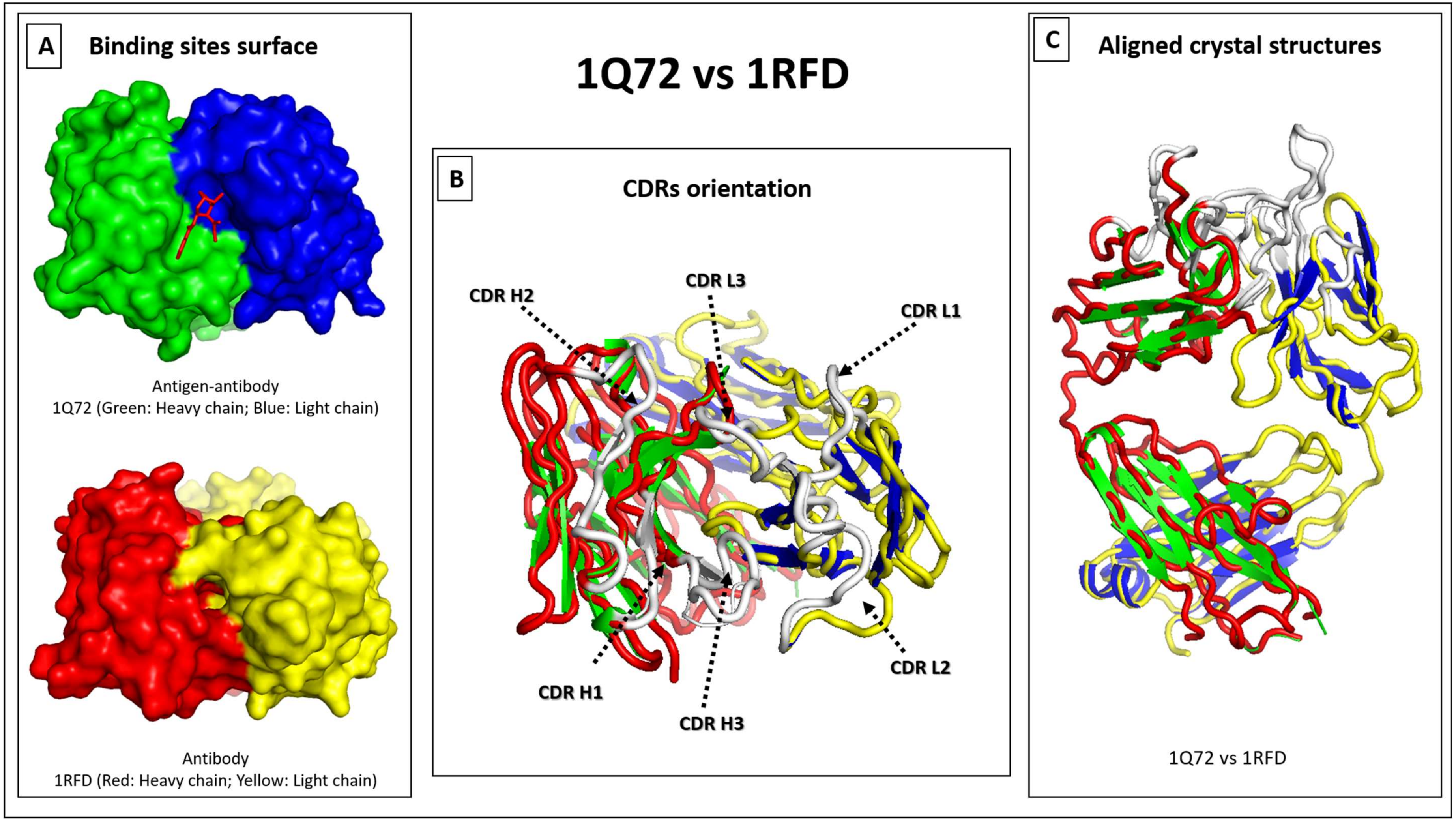

1Q72 vs 1RFD

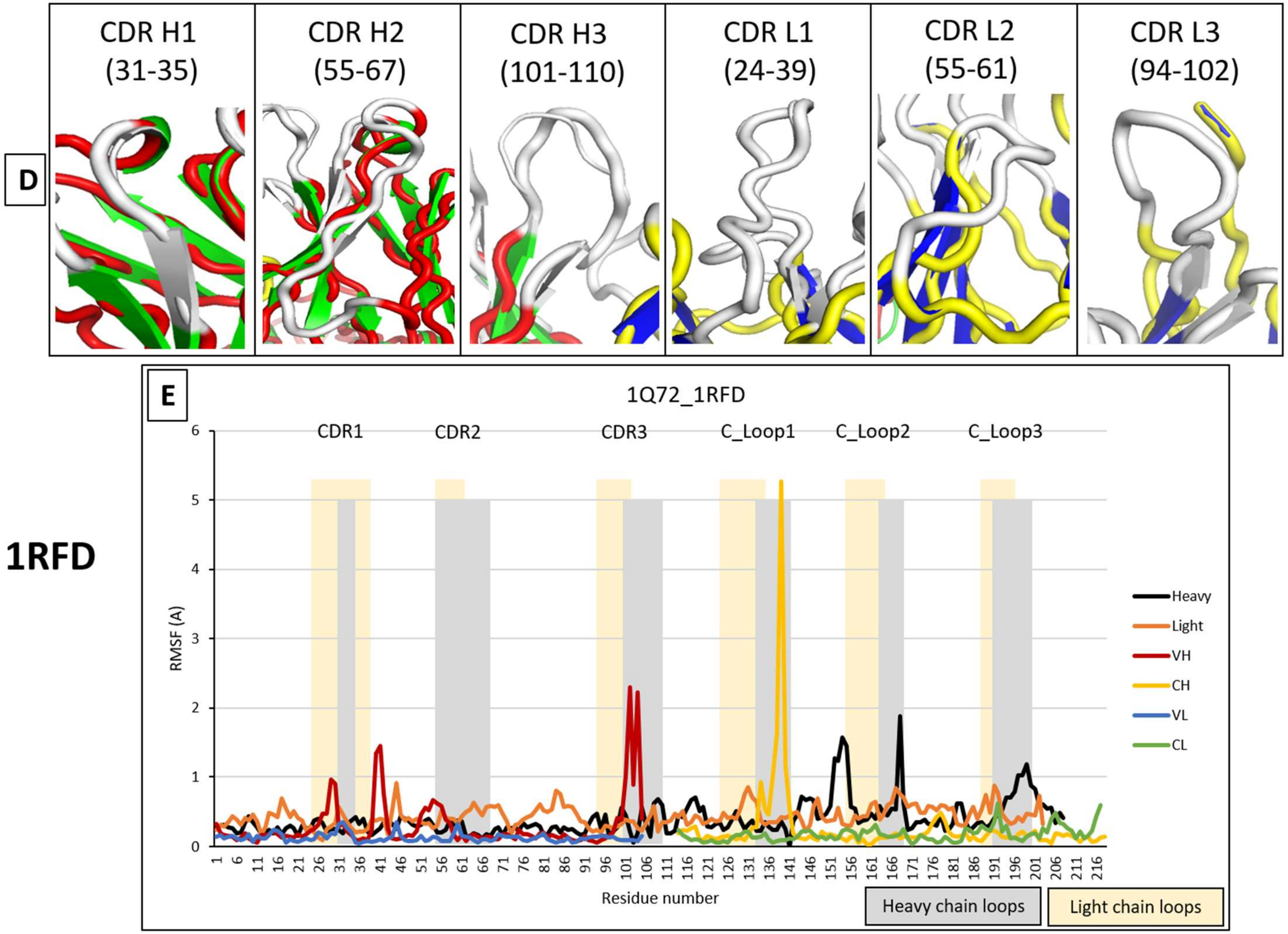
D
CDR H1 (31-35)
CDR H2 (55-67)
CDR H3 (101-110)
CDR L1 (24-39)
CDR L2 (55-61)
CDR L3 (94-102)
E
1Q72_1RFD
CDR1
CDR2
CDR3
C_Loop1
C_Loop2
C_Loop3
RMSF (A)
Residue number
Heavy
Light
VH
CH
VL
CL
Heavy chain loops
Light chain loops

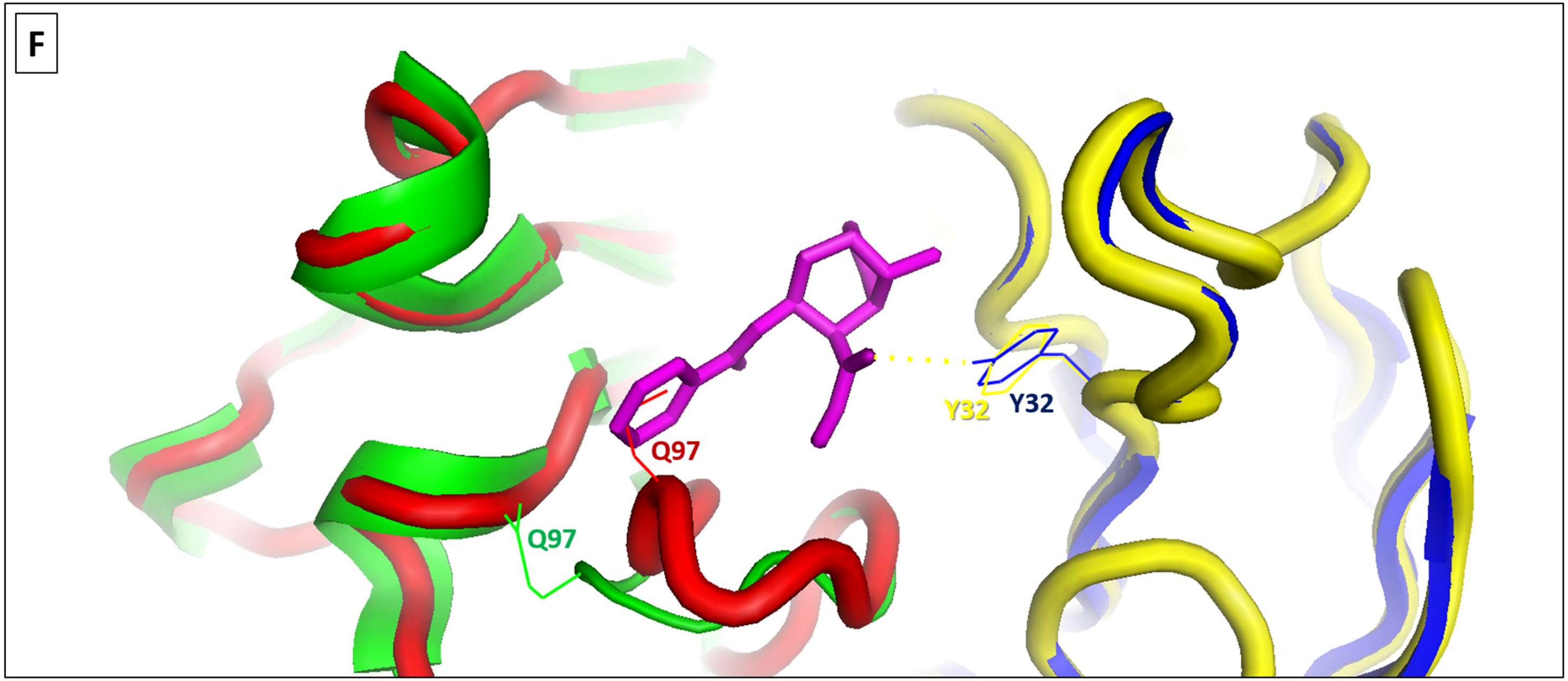
F
Q97
Q97
Y32
Y32

S.2.5 (1QYG vs 1RFD), mouse:

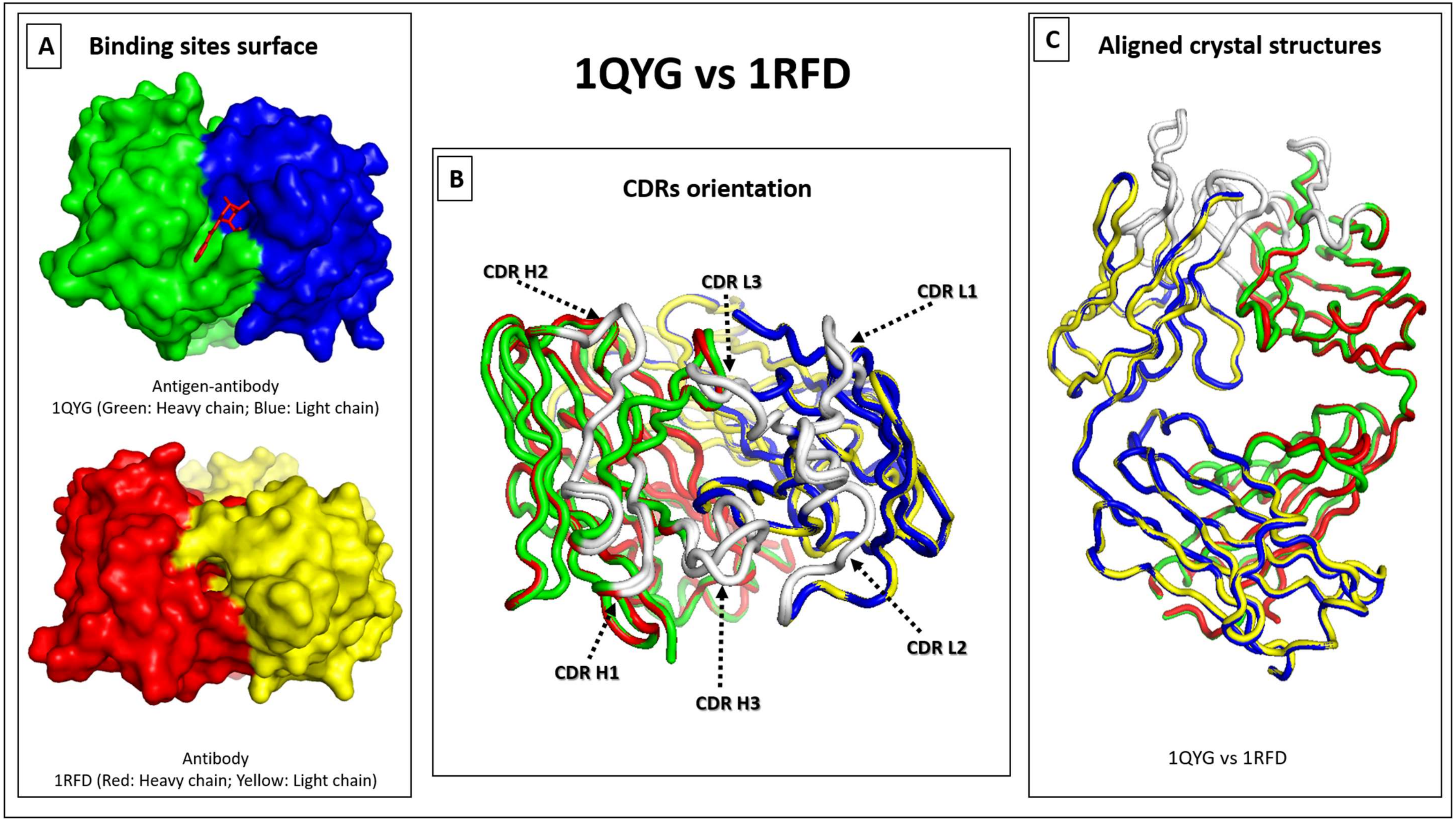

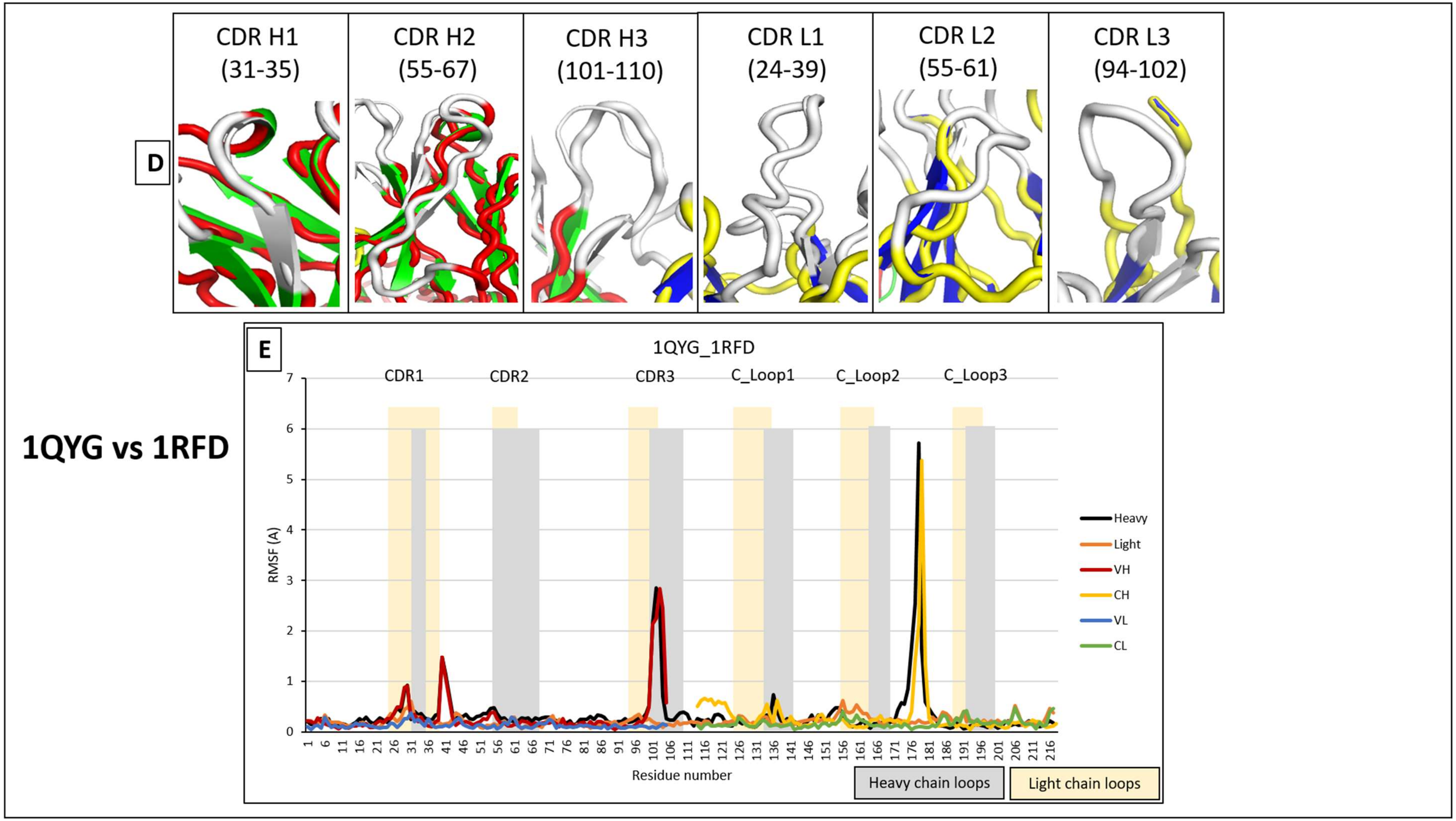
1QYG vs 1RFD
D
CDR H1 (31-35)
CDR H2 (55-67)
CDR H3 (101-110)
CDR L1 (24-39)
CDR L2 (55-61)
CDR L3 (94-102)
E
1QYG_1RFD
CDR1
CDR2
CDR3
C_Loop1
C_Loop2
C_Loop3
RMSF (A)
Residue number
Heavy
Light
VH
CH
VL
CL
Heavy chain loops
Light chain loops

S.2.6 (1JNN vs 1JNL), mouse:

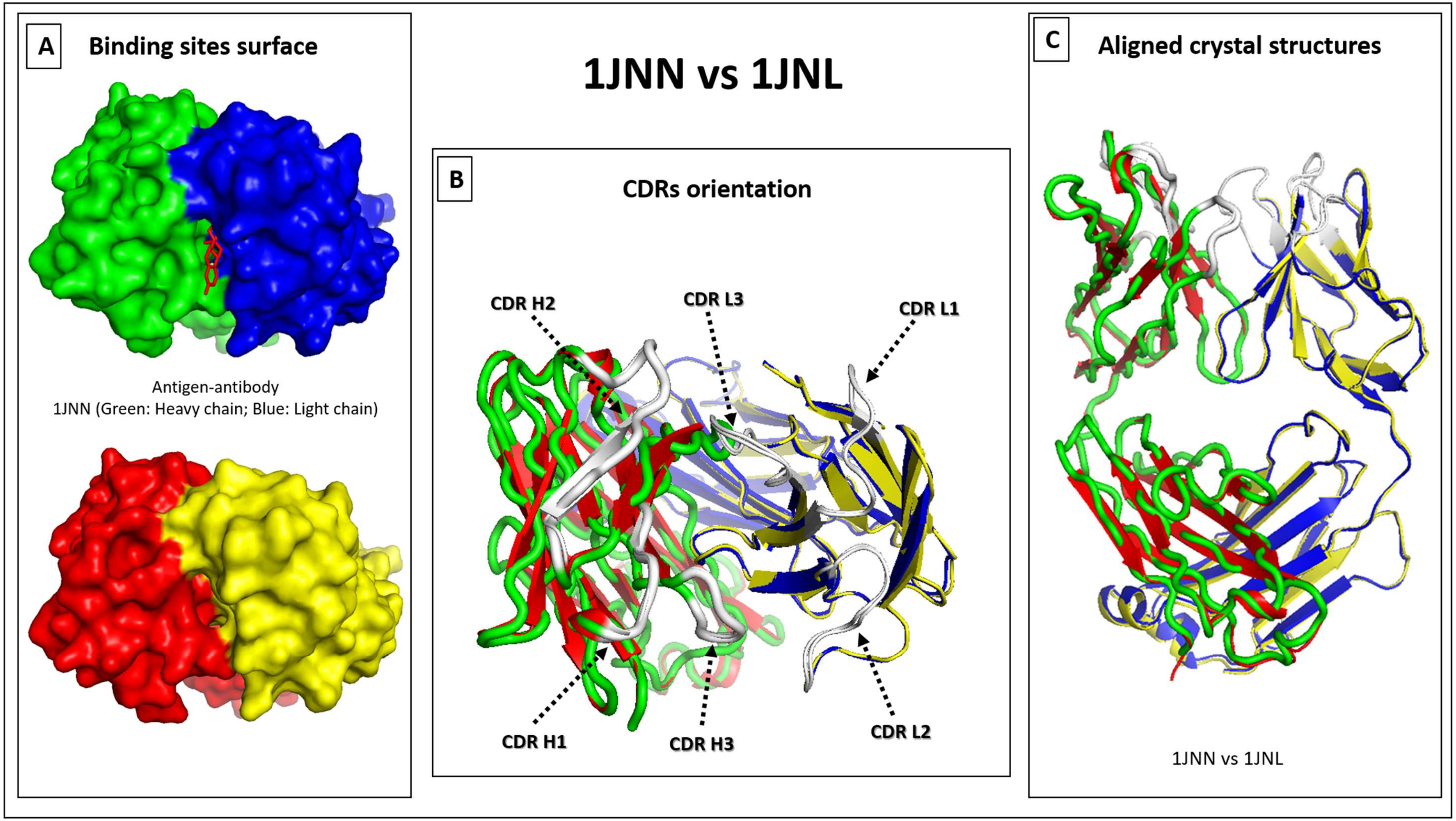

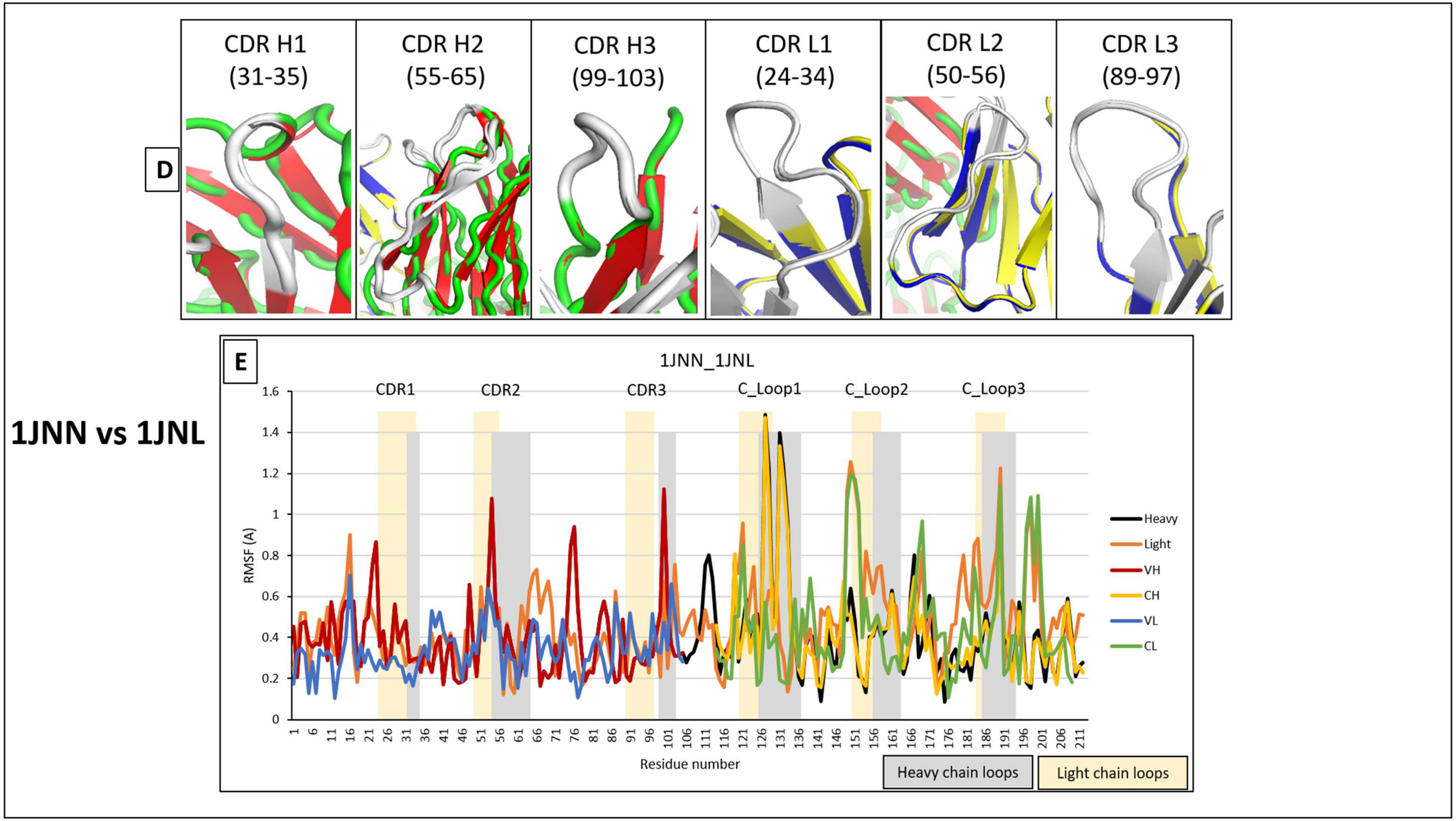
D
CDR H1 (31-35)
CDR H2 (55-65)
CDR H3 (99-103)
CDR L1 (24-34)
CDR L2 (50-56)
CDR L3 (89-97)
1JNN vs 1JNL
E
1JNN_1JNL
CDR1
CDR2
CDR3
C_Loop1
C_Loop2
C_Loop3
RMSF (A)
Residue number
Heavy
Light
VH
CH
VL
CL
Heavy chain loops
Light chain loops

S.2.7 (1KEL vs 1KEM), mouse:

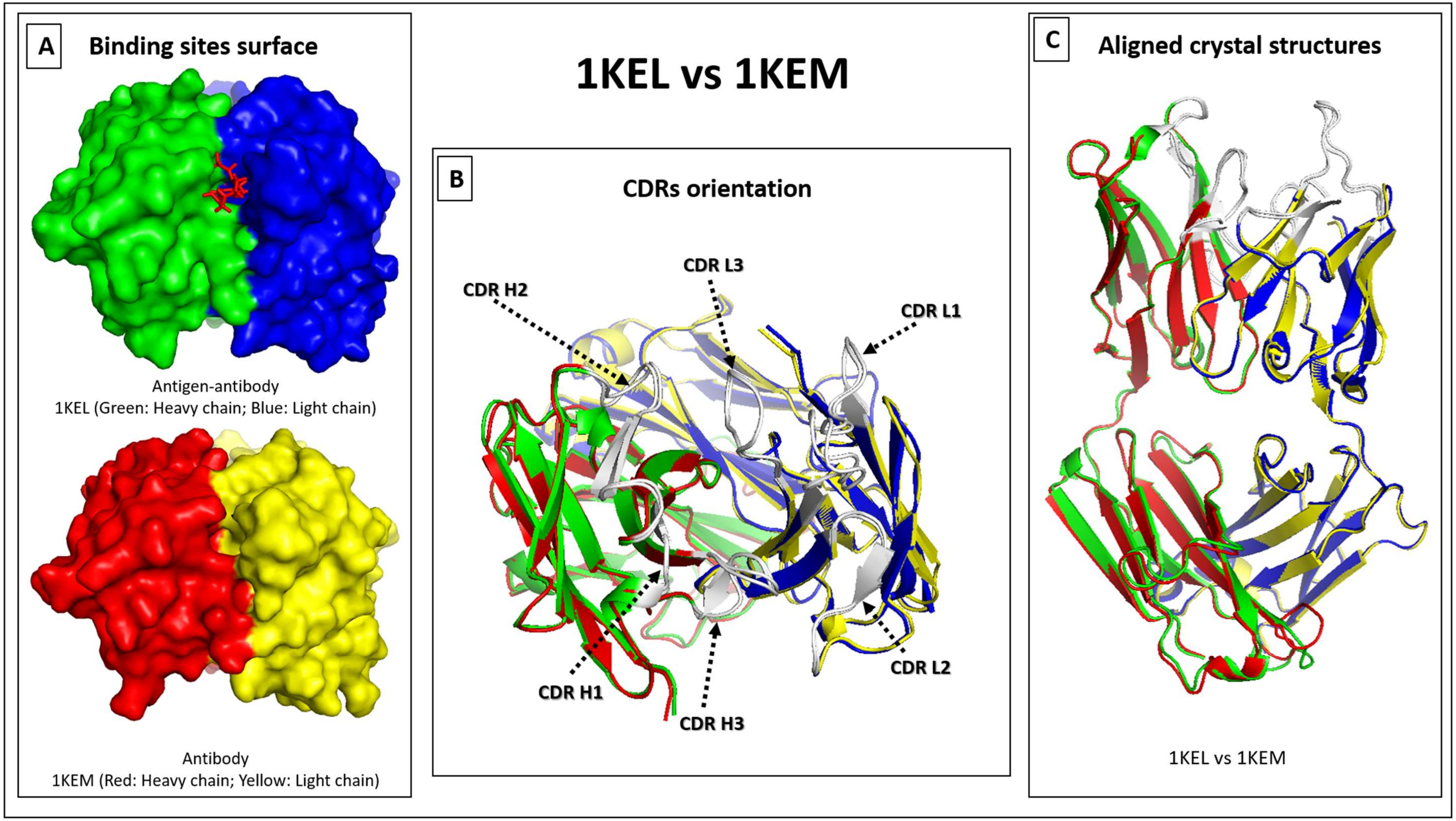

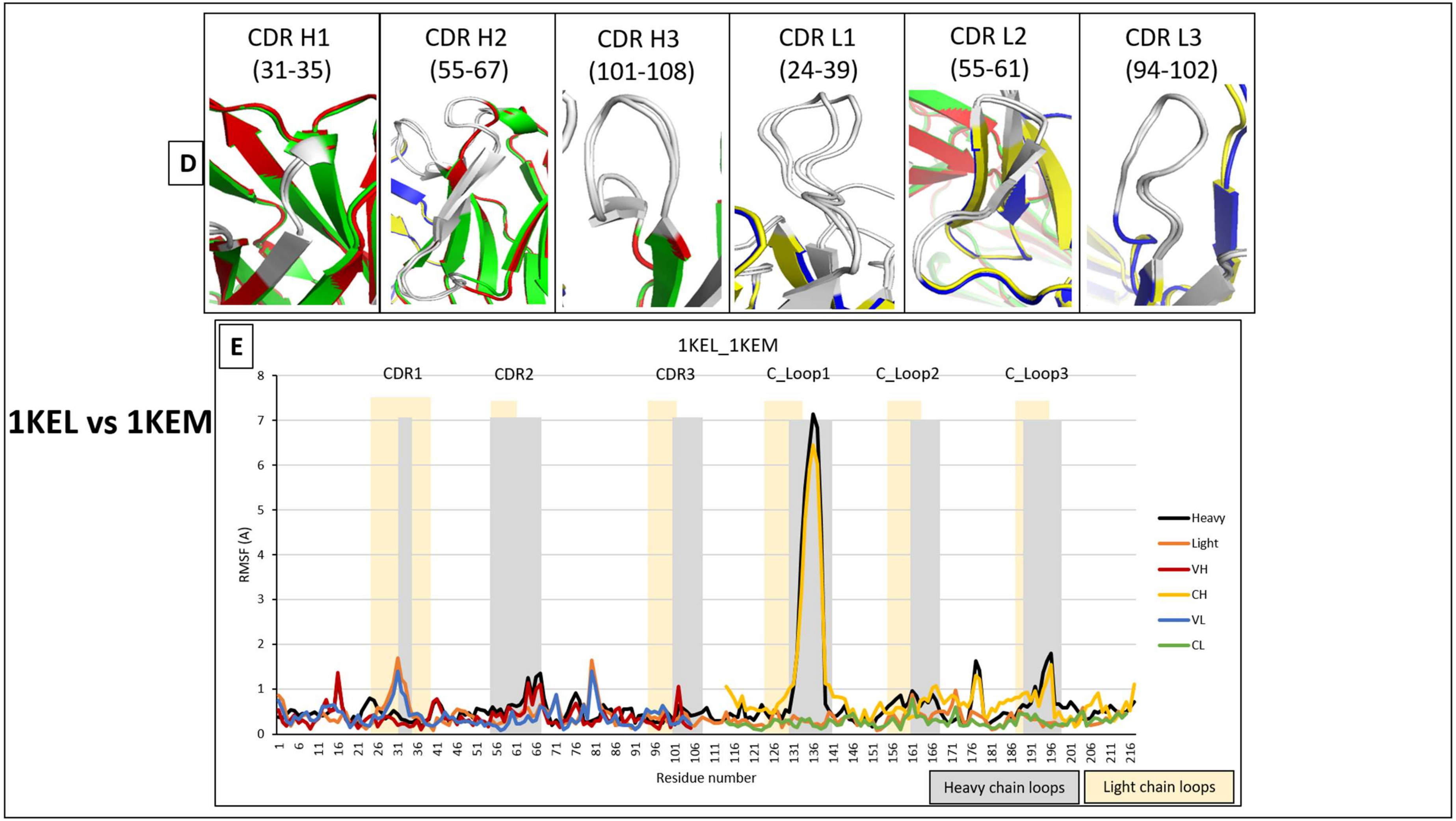
1KEL vs 1KEM
D
CDR H1 (31-35)
CDR H2 (55-67)
CDR H3 (101-108)
CDR L1 (24-39)
CDR L2 (55-61)
CDR L3 (94-102)
E
1KEL_1KEM
CDR1
CDR2
CDR3
C_Loop1
C_Loop2
C_Loop3
RMSF (A)
Residue number
Heavy
Light
VH
CH
VL
CL
Heavy chain loops
Light chain loops

S.2.8 (1NGP vs 1NGQ), mouse:

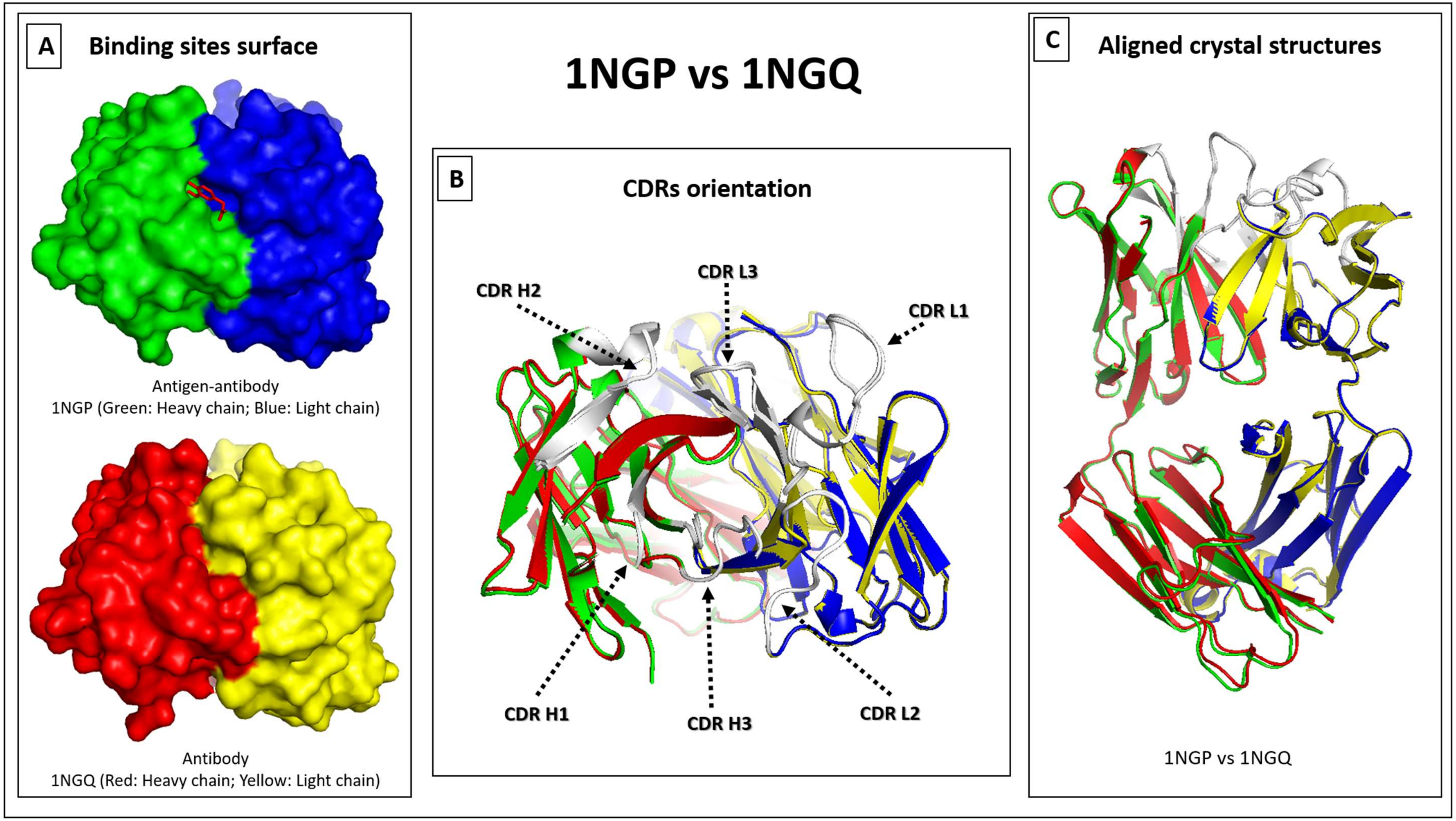

1NGP vs 1NGQ

D

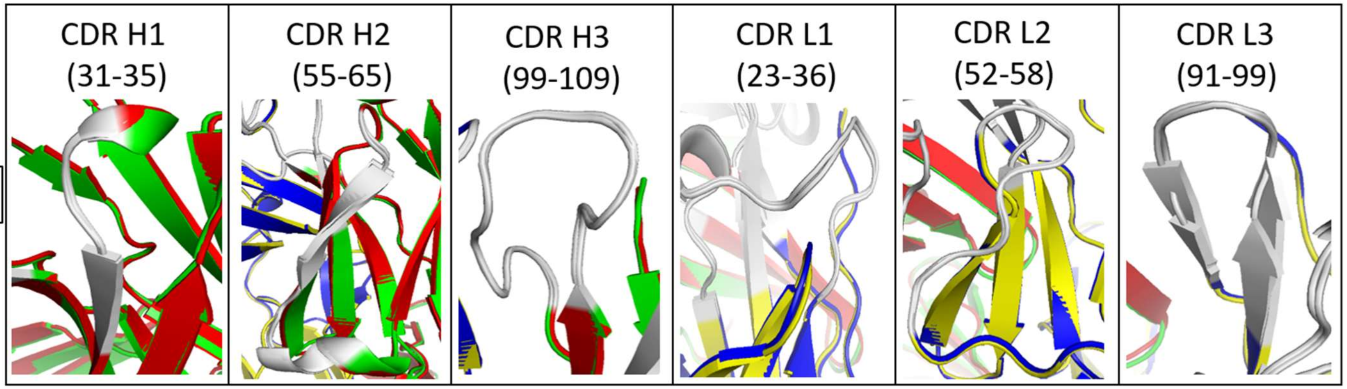

E

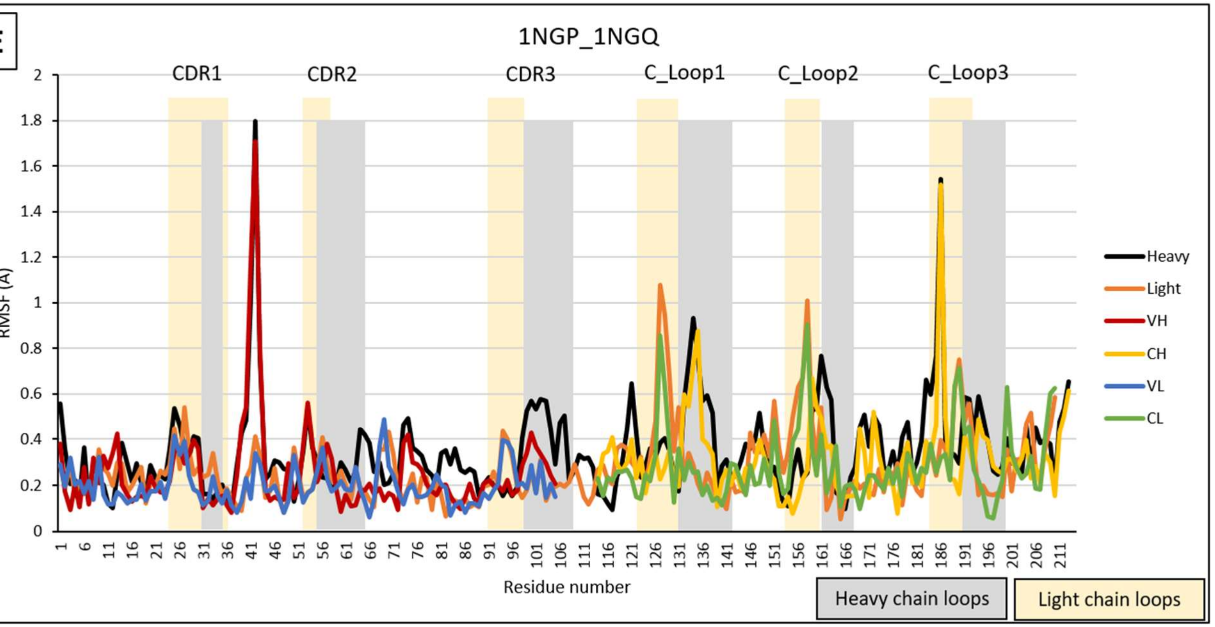

S.2.9 (2CGR vs 1CGS), mouse:

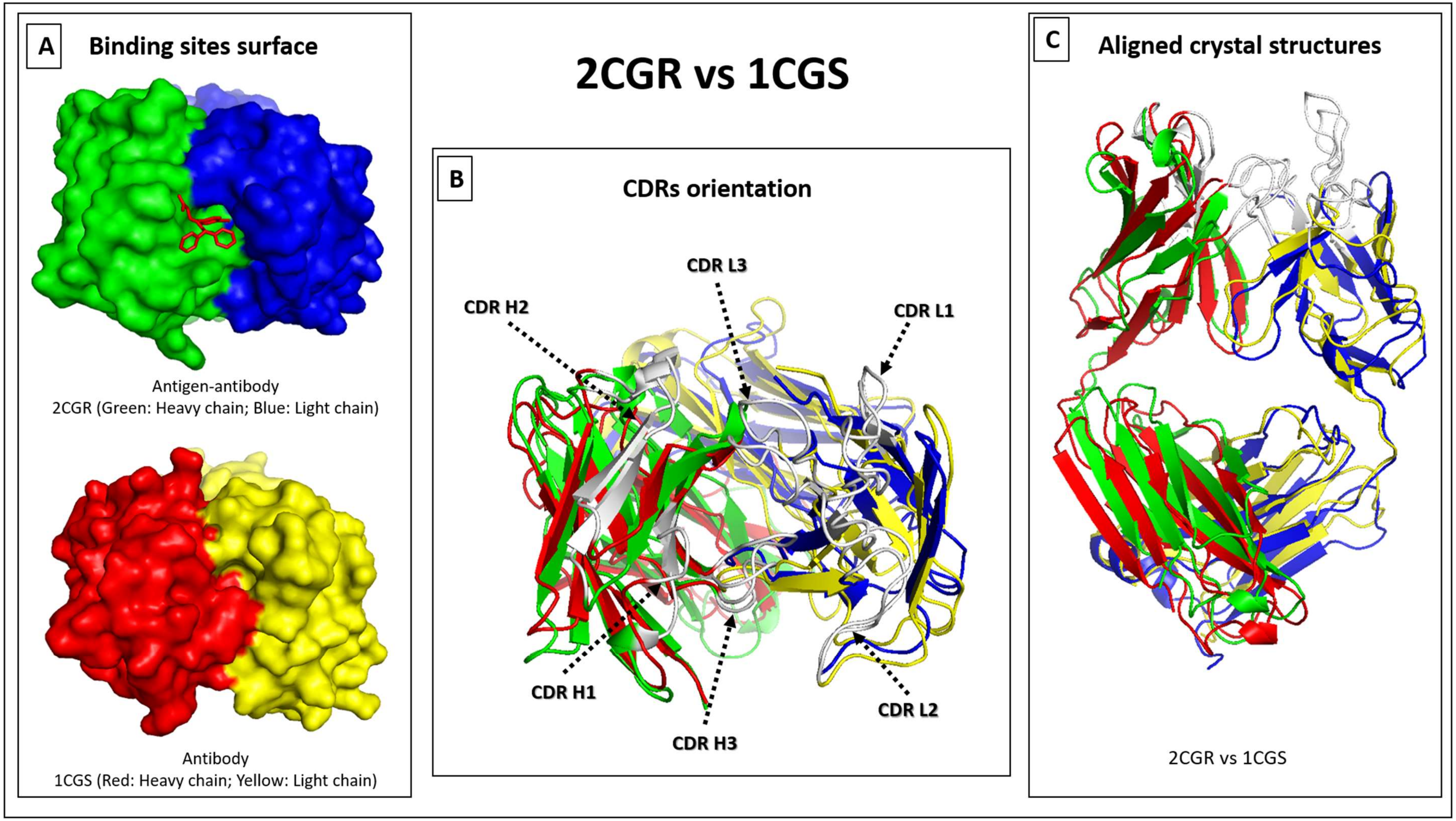

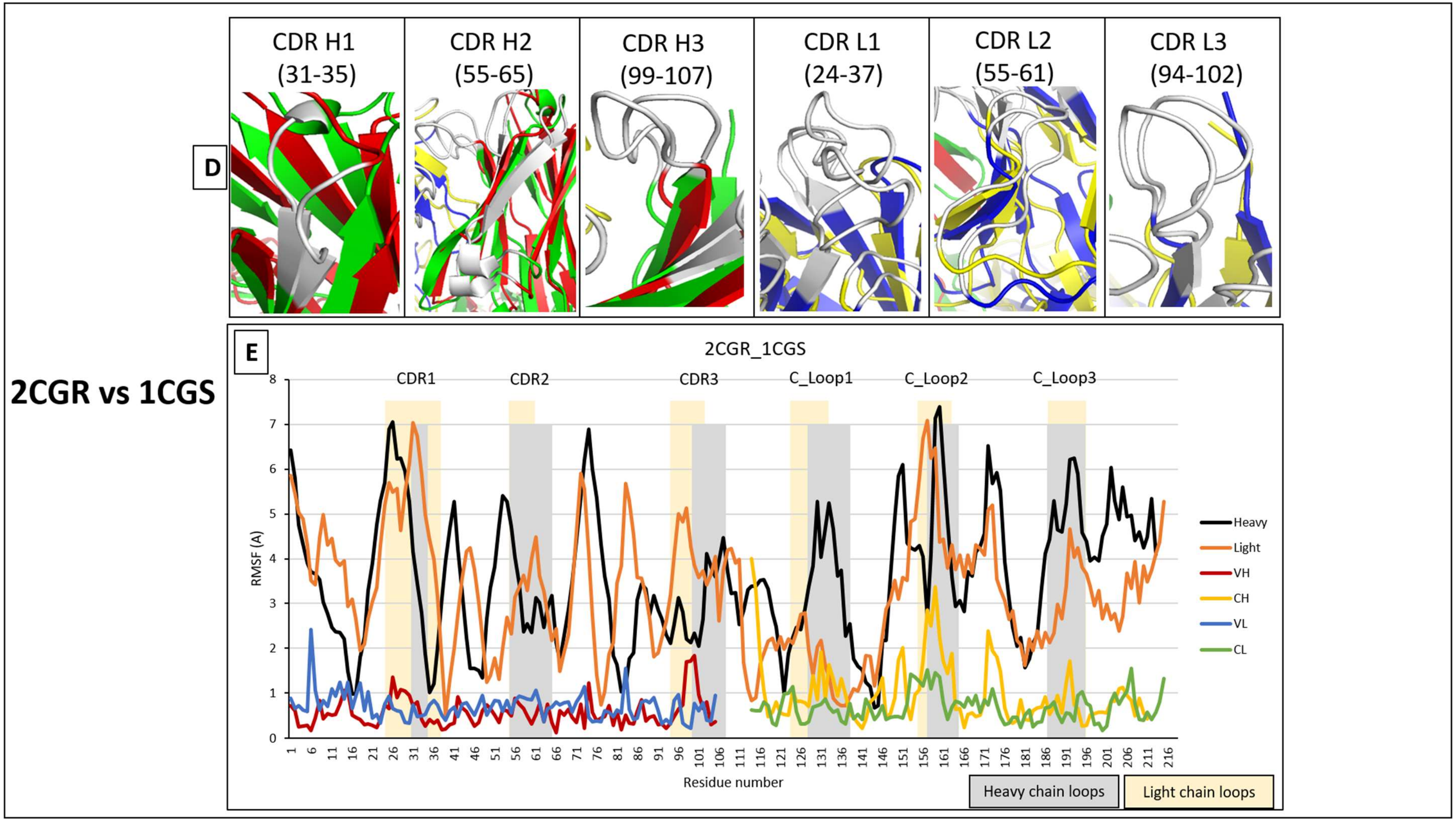

2CGR vs 1CGS
D
CDR H1 (31-35)
CDR H2 (55-65)
CDR H3 (99-107)
CDR L1 (24-37)
CDR L2 (55-61)
CDR L3 (94-102)
E
2CGR_1CGS
CDR1
CDR2
CDR3
C_Loop1
C_Loop2
C_Loop3
RMSF (A)
Residue number
Heavy
Light
VH
CH
VL
CL
Heavy chain loops
Light chain loops

S.2.10 (1Q0Y vs 1Q0X), mouse:

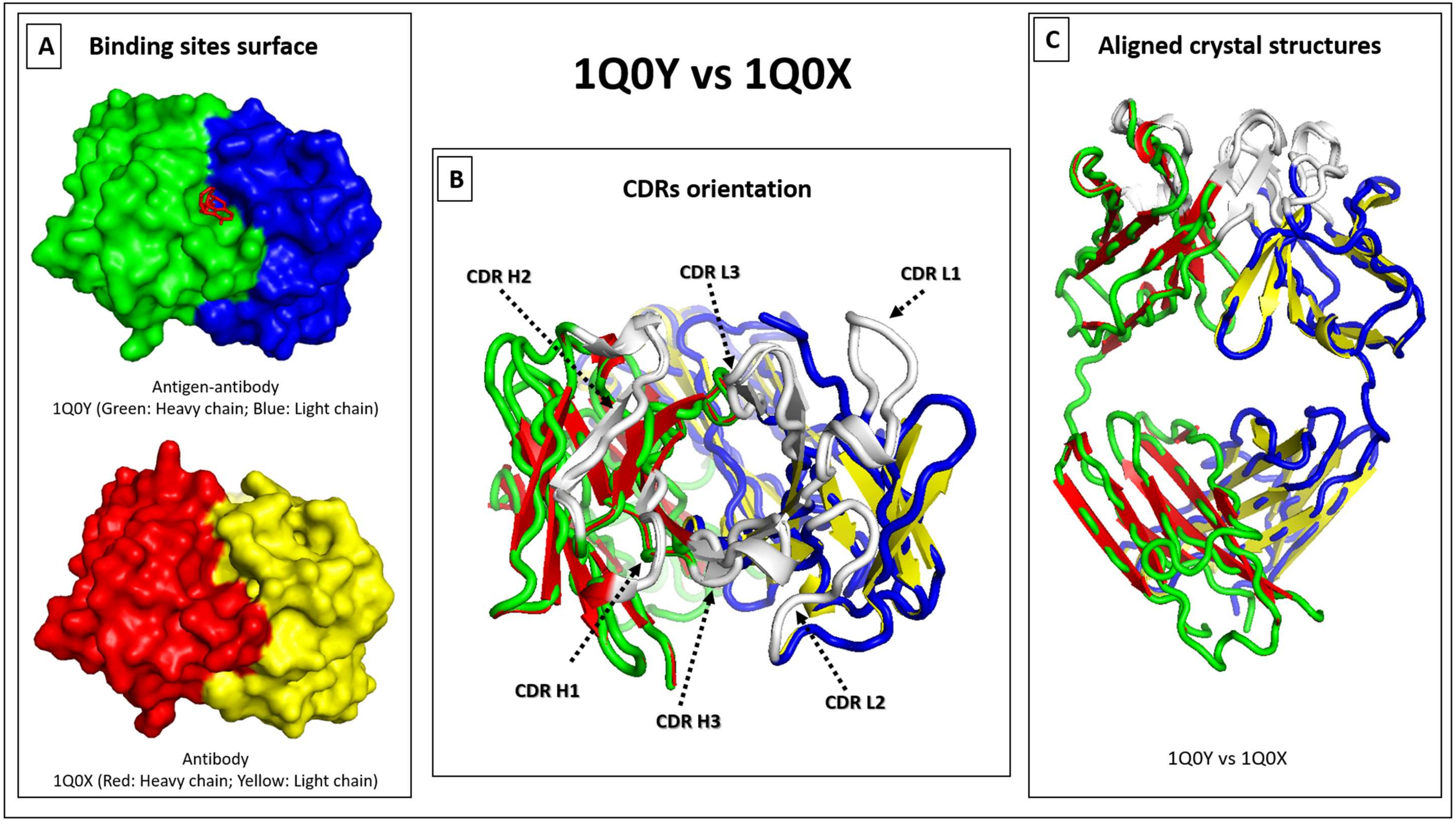

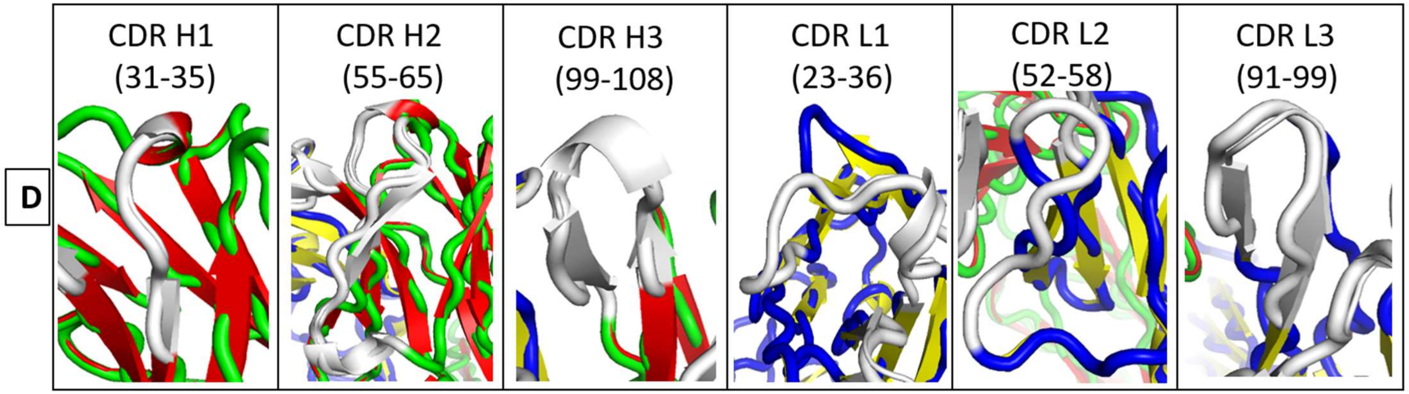
D
CDR H1 (31-35)
CDR H2 (55-65)
CDR H3 (99-108)
CDR L1 (23-36)
CDR L2 (52-58)
CDR L3 (91-99)

1Q0Y vs 1Q0X

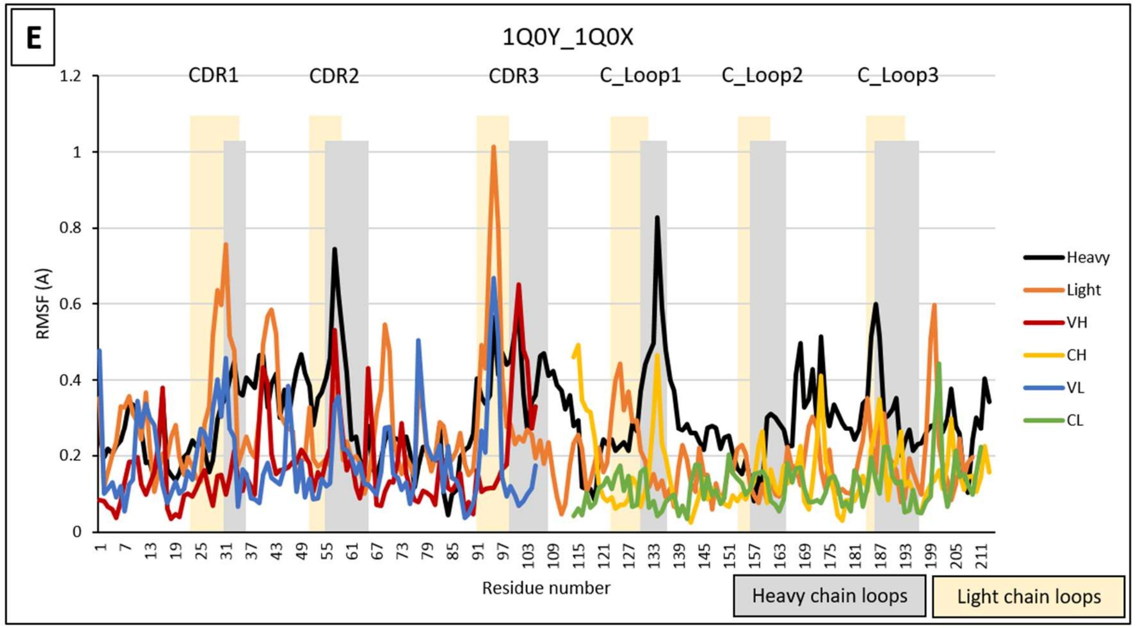
E
1Q0Y_1Q0X
CDR1
CDR2
CDR3
C_Loop1
C_Loop2
C_Loop3
RMSF (A)
0
0.2
0.4
0.6
0.8
1
1.2
Residue number
1 7 13 19 25 31 37 43 49 55 61 67 73 79 85 91 97 103 109 115 121 127 133 139 145 151 157 163 169 175 181 187 193 199 205 211
Heavy
Light
VH
CH
VL
CL
Heavy chain loops
Light chain loops

S.2.11 (2AJS vs 2AJU), mouse:

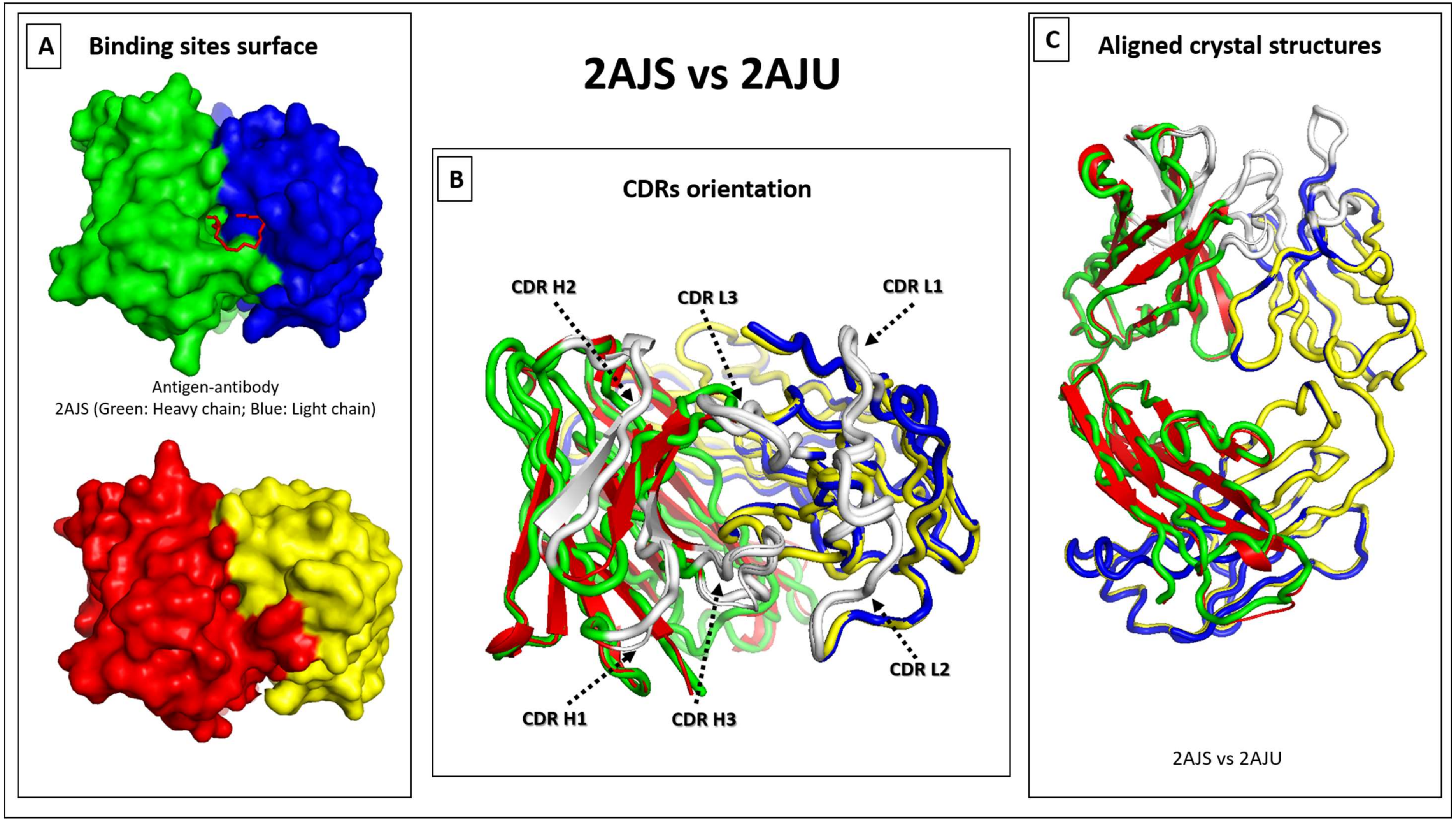

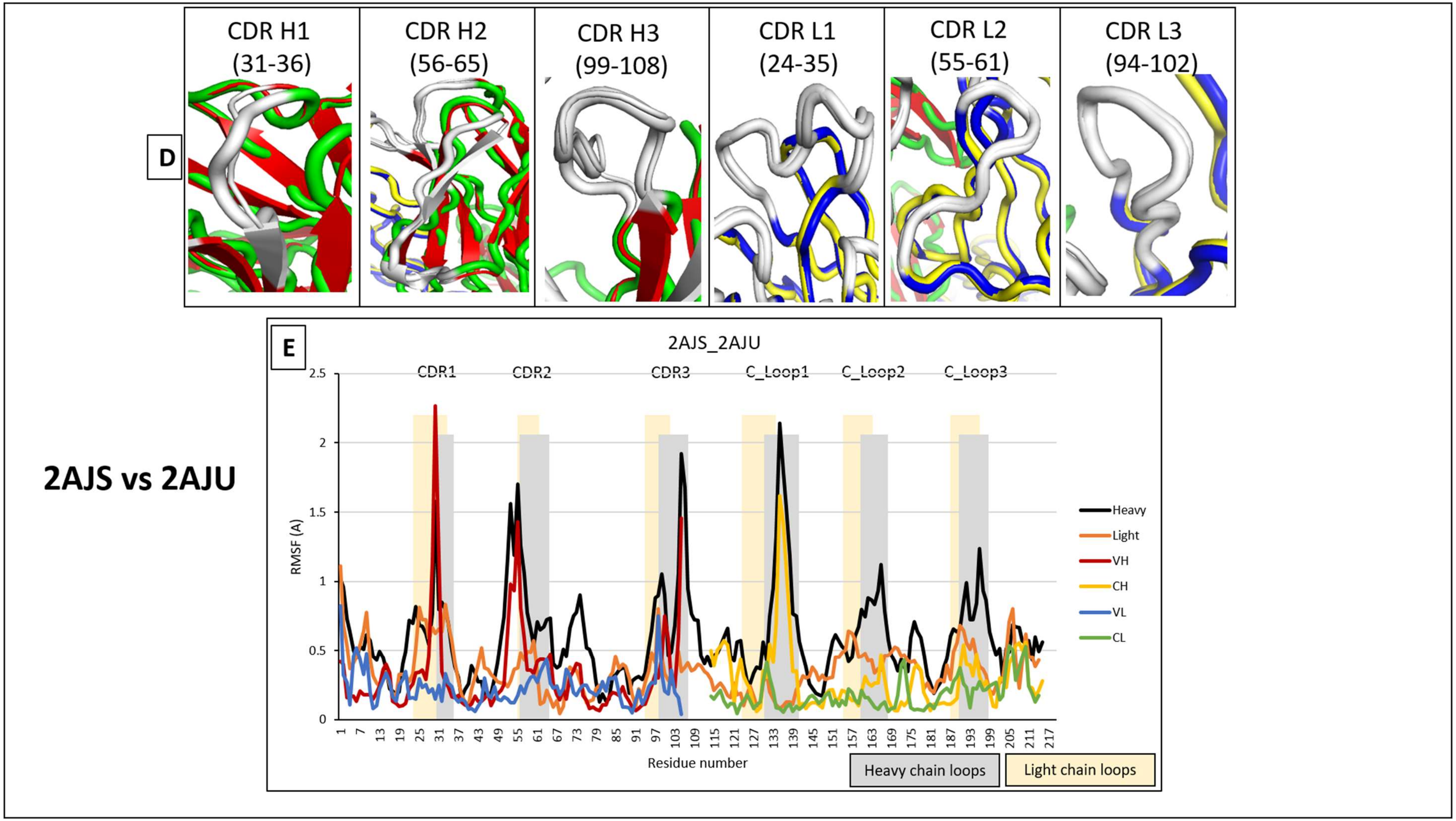

D
CDR H1
(31-36)
CDR H2
(56-65)
CDR H3
(99-108)
CDR L1
(24-35)
CDR L2
(55-61)
CDR L3
(94-102)
2AJS vs 2AJU
E
2AJS_2AJU
CDR1
CDR2
CDR3
C_Loop1
C_Loop2
C_Loop3
RMSF (A)
Residue number
Heavy
Light
VH
CH
VL
CL
Heavy chain loops
Light chain loops

S.2.12 (2AJV vs 2AJU), mouse:

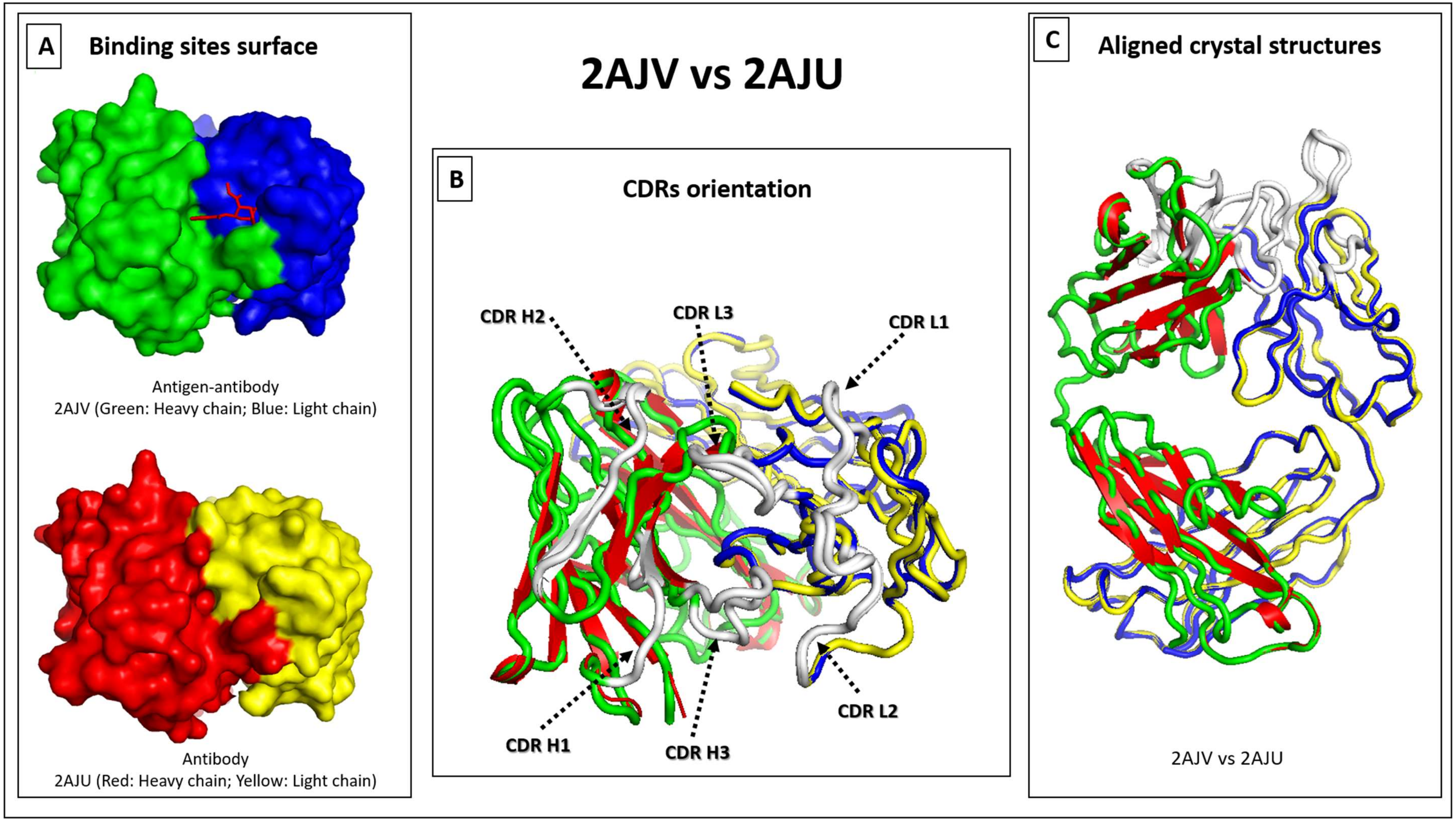

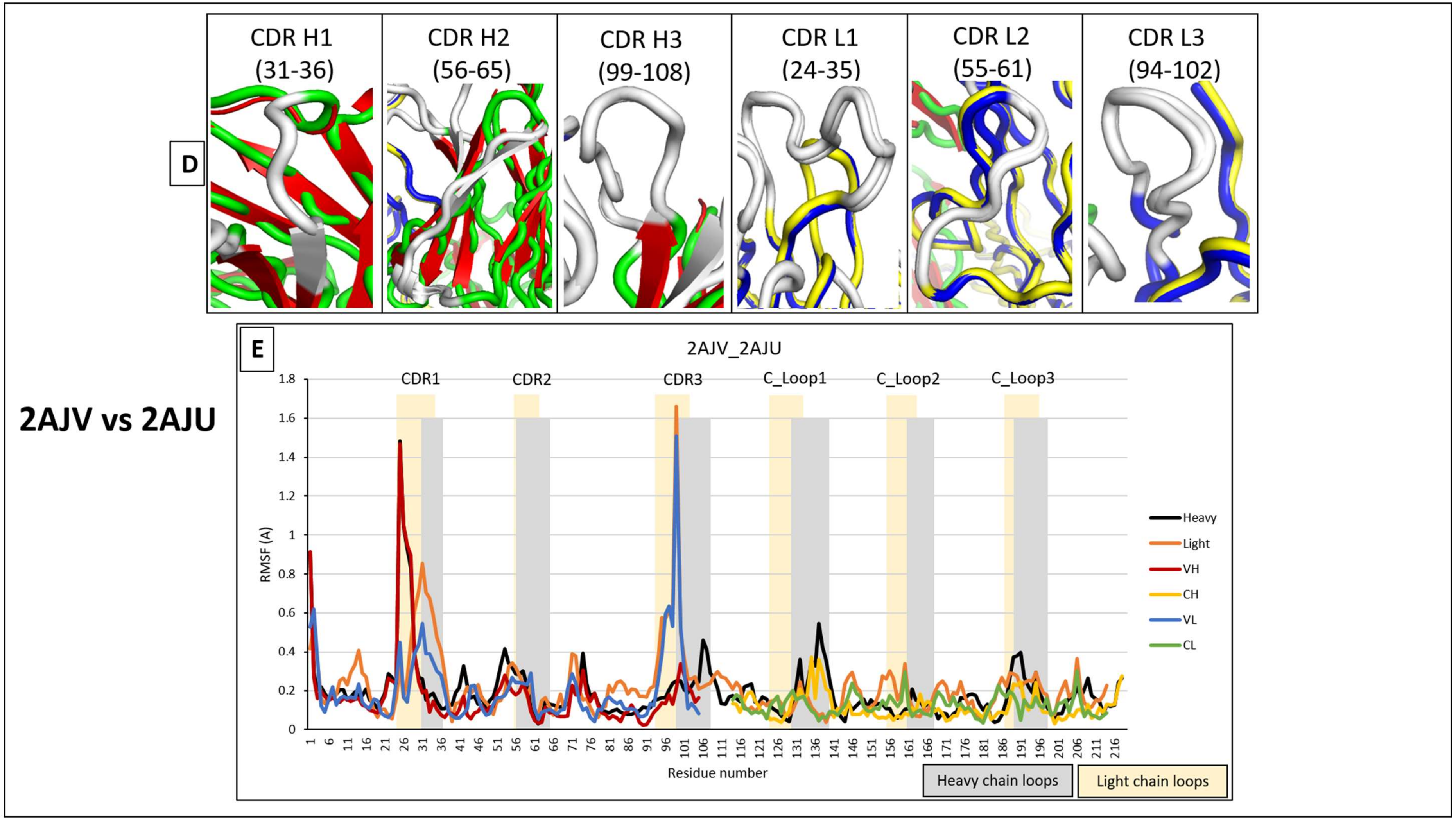
2AJV vs 2AJU
D
CDR H1 (31-36)
CDR H2 (56-65)
CDR H3 (99-108)
CDR L1 (24-35)
CDR L2 (55-61)
CDR L3 (94-102)
E
2AJV_2AJU
CDR1
CDR2
CDR3
C_Loop1
C_Loop2
C_Loop3
RMSF (A)
Residue number
Heavy
Light
VH
CH
VL
CL
Heavy chain loops
Light chain loops

S.2.13 (2AJX vs 2AJU), mouse:

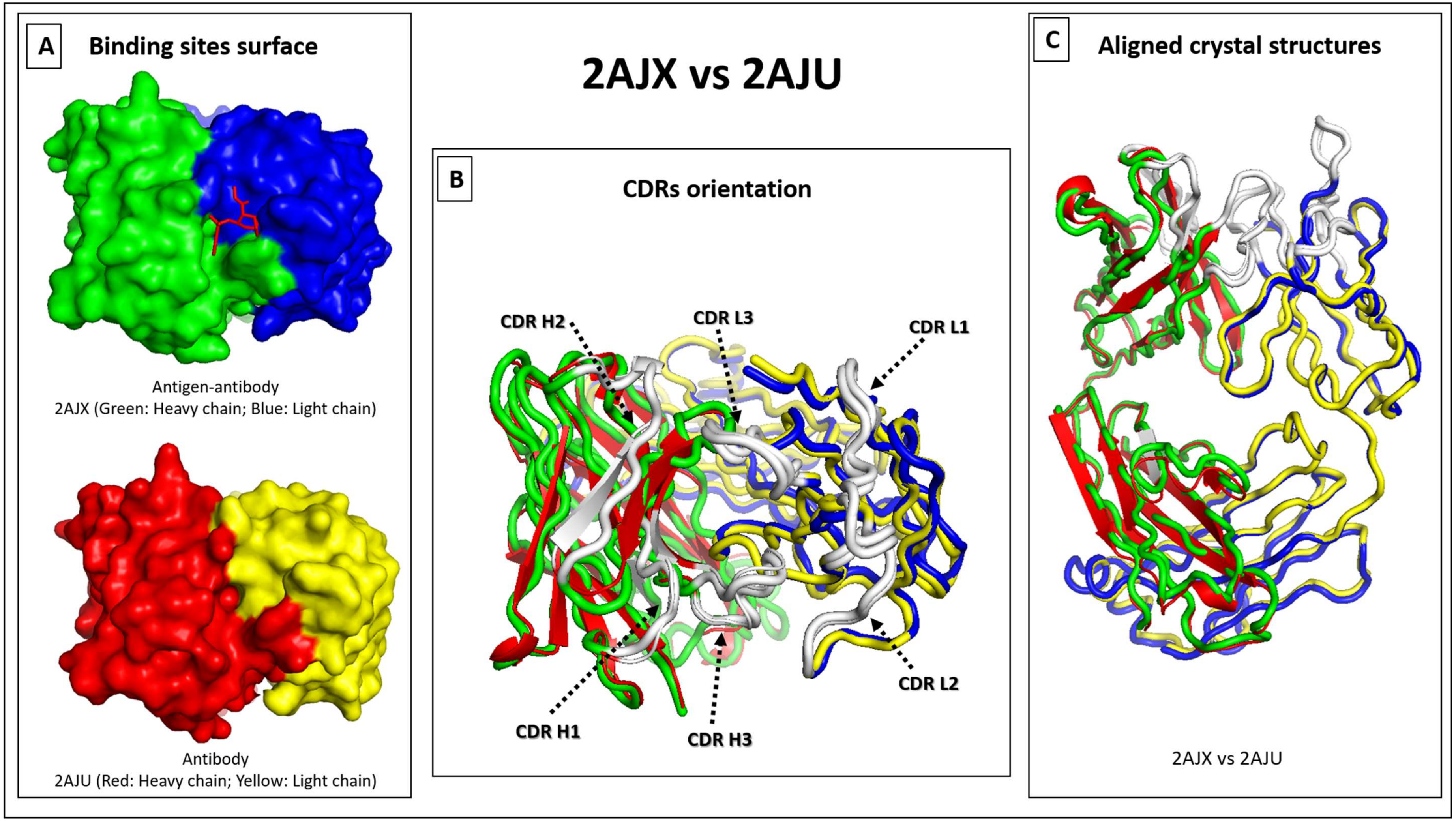

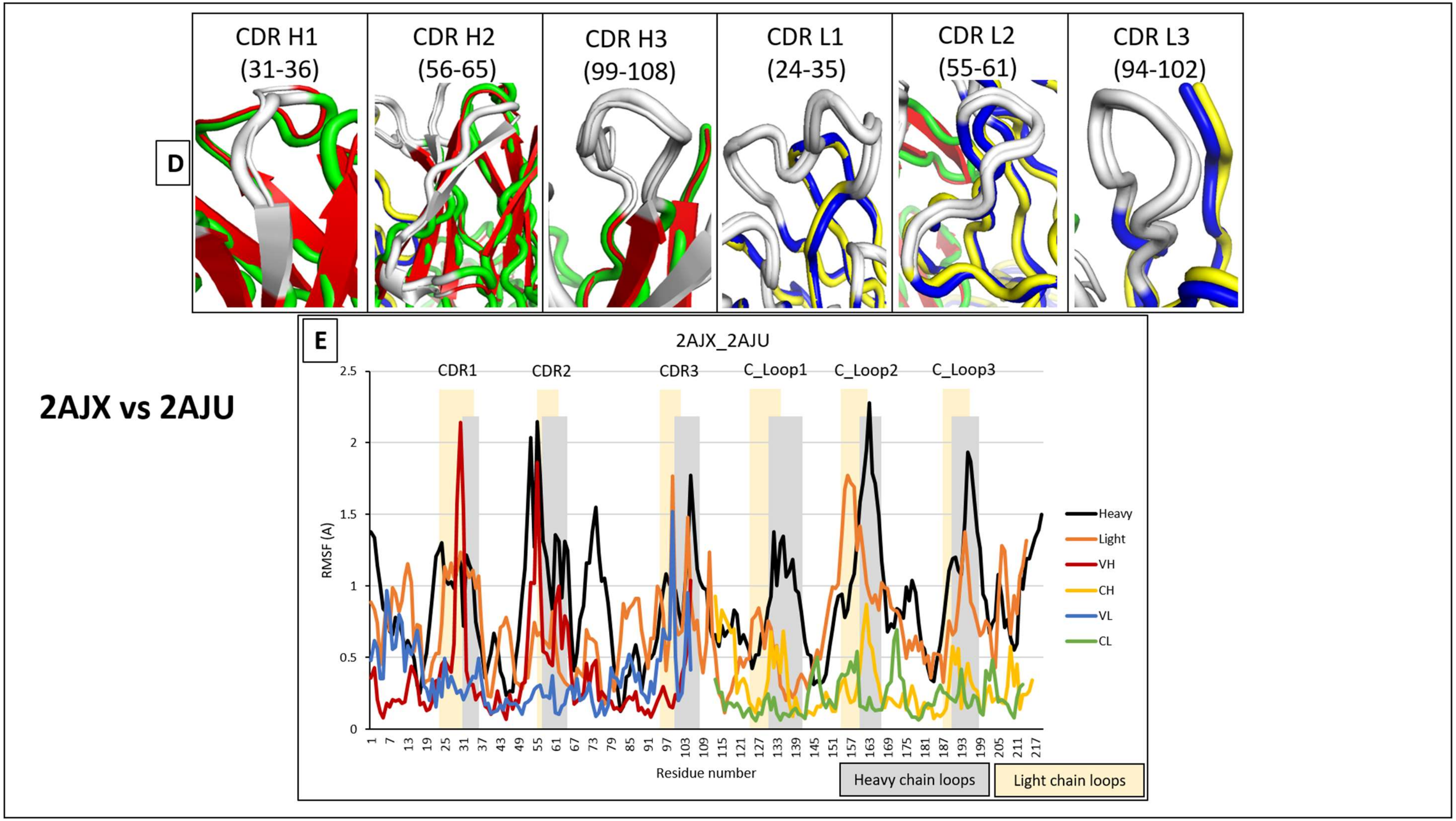
D
CDR H1
(31-36)
CDR H2
(56-65)
CDR H3
(99-108)
CDR L1
(24-35)
CDR L2
(55-61)
CDR L3
(94-102)
2AJX vs 2AJU
E
2AJX_2AJU
CDR1
CDR2
CDR3
C_Loop1
C_Loop2
C_Loop3
RMSF (A)
Residue number
Heavy
Light
VH
CH
VL
CL
Heavy chain loops
Light chain loops

S.2.14 (2AJY vs 2AJU), mouse:

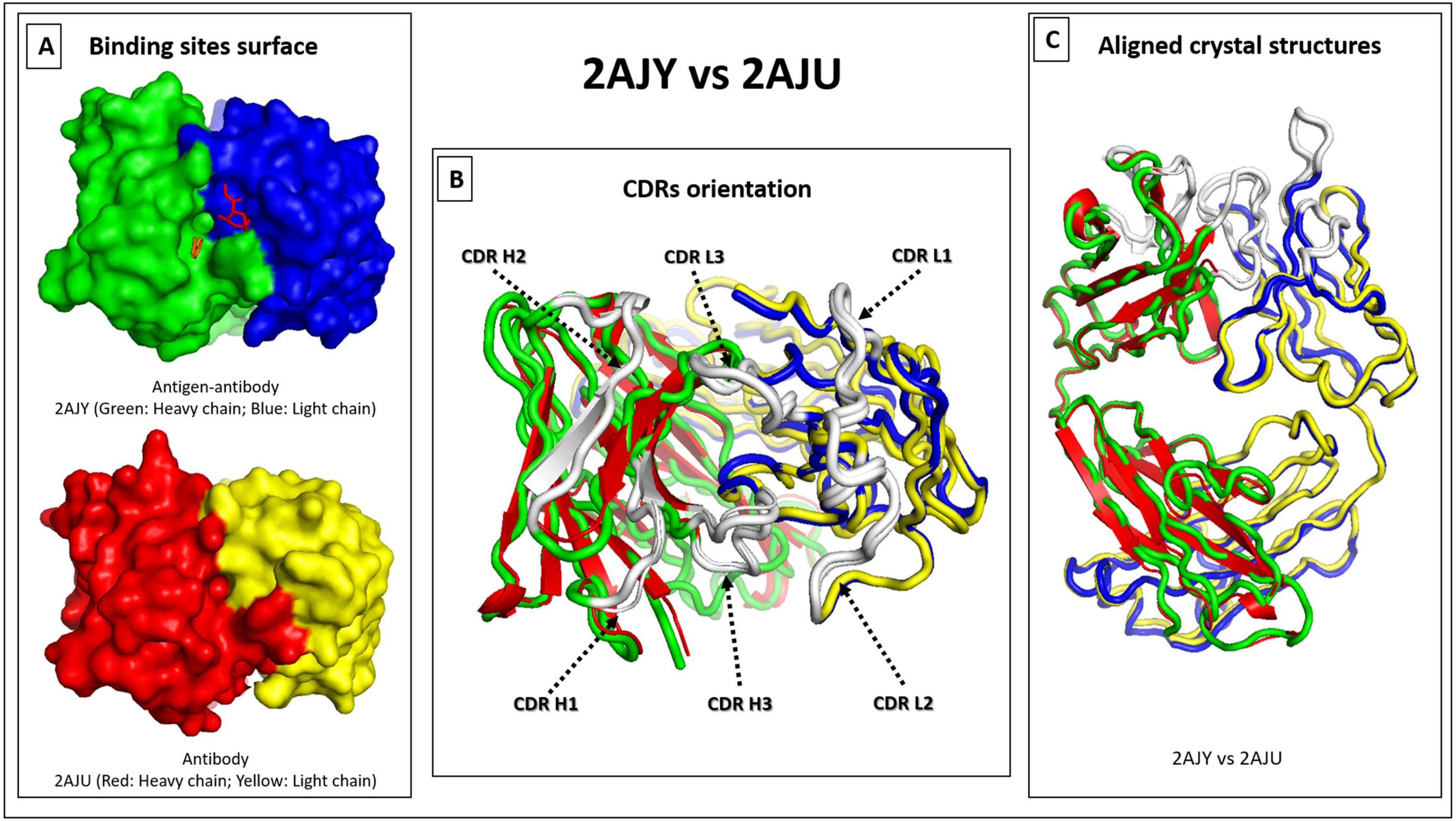

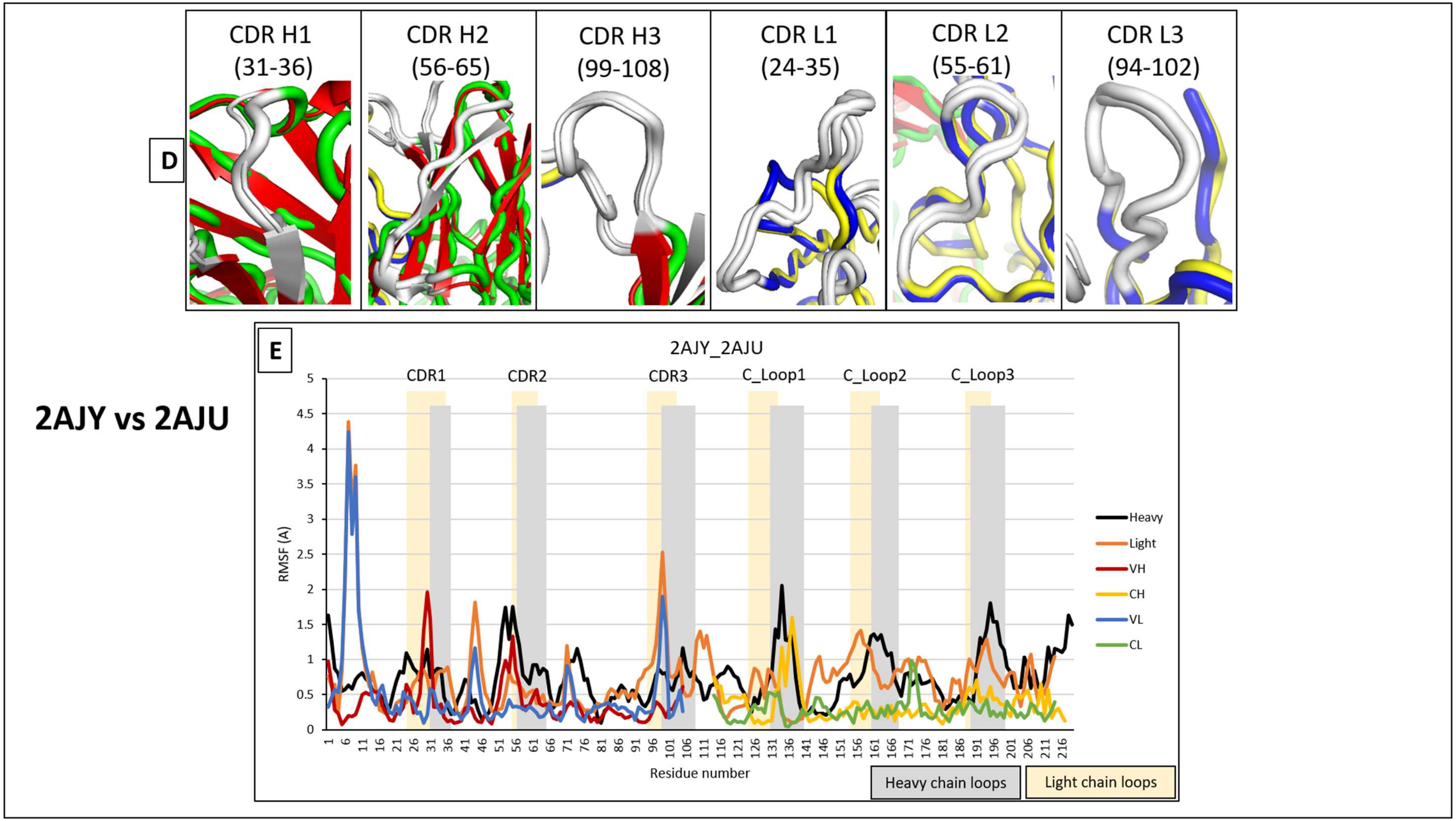
D
CDR H1 (31-36)
CDR H2 (56-65)
CDR H3 (99-108)
CDR L1 (24-35)
CDR L2 (55-61)
CDR L3 (94-102)
2AJY vs 2AJU
E
2AJY_2AJU
CDR1
CDR2
CDR3
C_Loop1
C_Loop2
C_Loop3
RMSF (A)
Residue number
Heavy
Light
VH
CH
VL
CL
Heavy chain loops
Light chain loops

S.2.15 (2AJZ vs 2AJU), mouse:

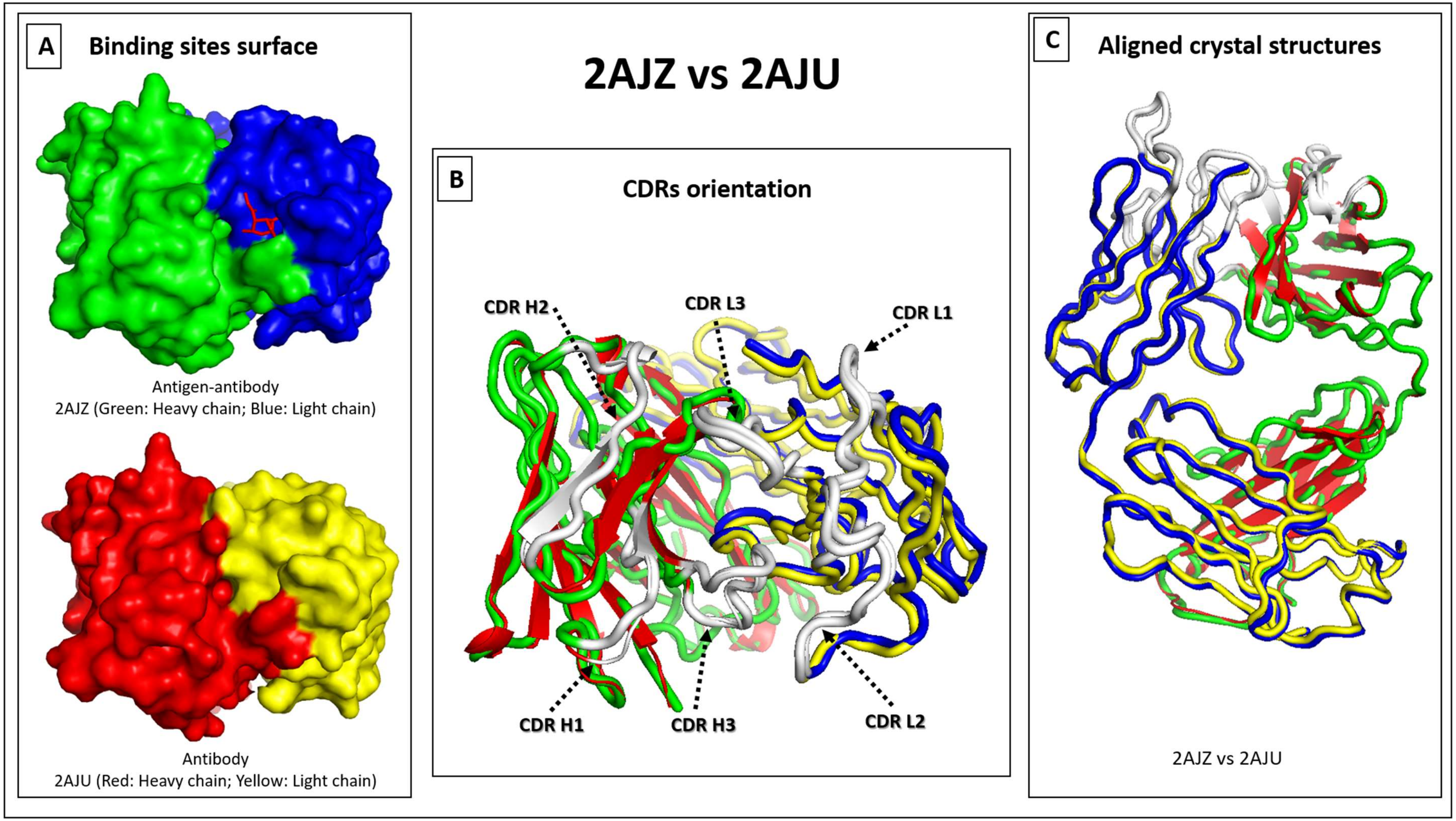

2AJZ vs 2AJU

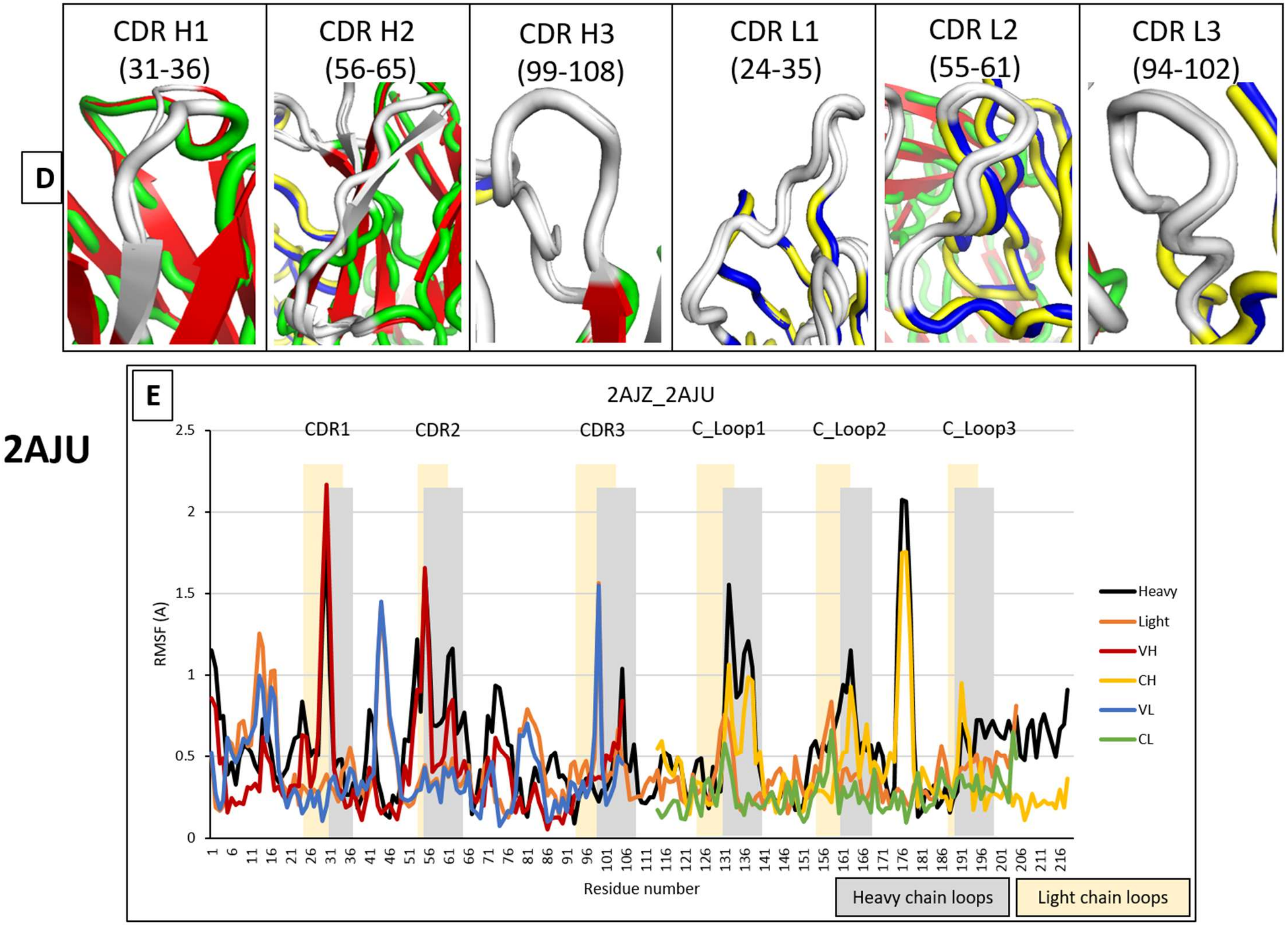

D
CDR H1 (31-36)
CDR H2 (56-65)
CDR H3 (99-108)
CDR L1 (24-35)
CDR L2 (55-61)
CDR L3 (94-102)
E
2AJZ_2AJU
CDR1
CDR2
CDR3
C_Loop1
C_Loop2
C_Loop3
RMSF (A)
Residue number
Heavy
Light
VH
CH
VL
CL
Heavy chain loops
Light chain loops

S.2.16 (2AK1 vs 2AJU), mouse:

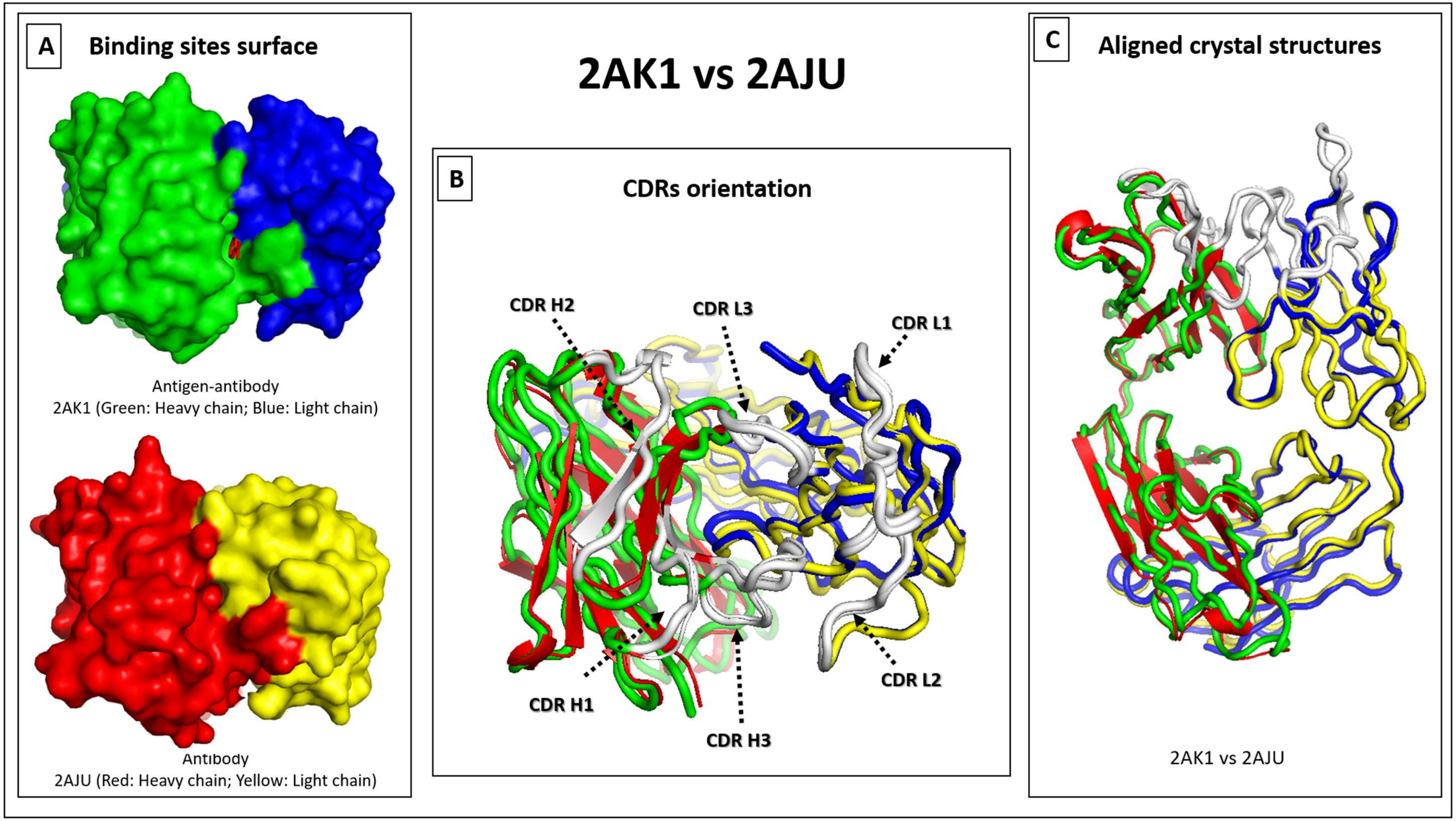

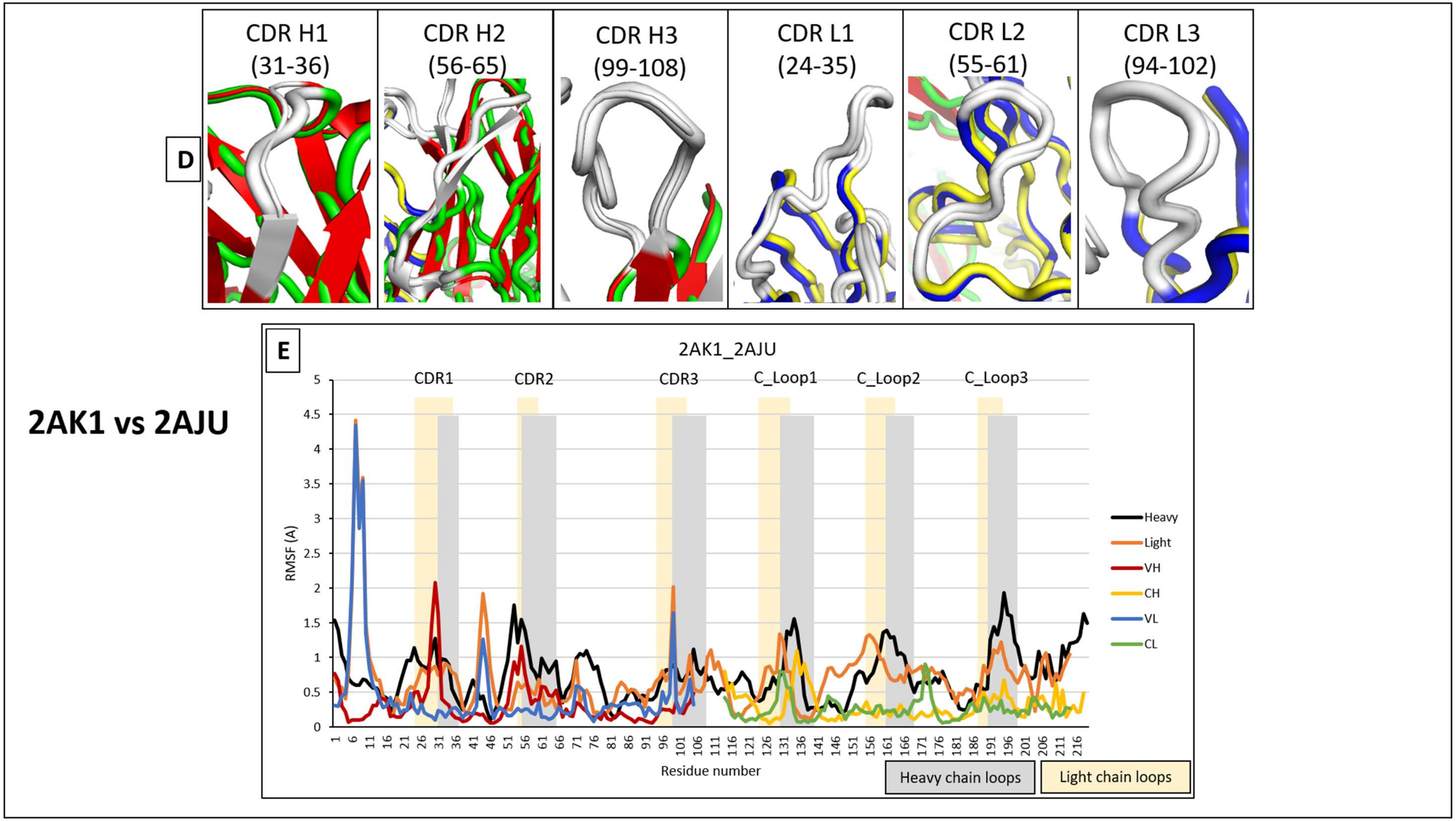
D
CDR H1 (31-36)
CDR H2 (56-65)
CDR H3 (99-108)
CDR L1 (24-35)
CDR L2 (55-61)
CDR L3 (94-102)
2AK1 vs 2AJU
E
2AK1_2AJU
CDR1
CDR2
CDR3
C_Loop1
C_Loop2
C_Loop3
RMSF (A)
Residue number
Heavy
Light
VH
CH
VL
CL
Heavy chain loops
Light chain loops

S.2.17 (4OCX vs 4OCY), mouse:

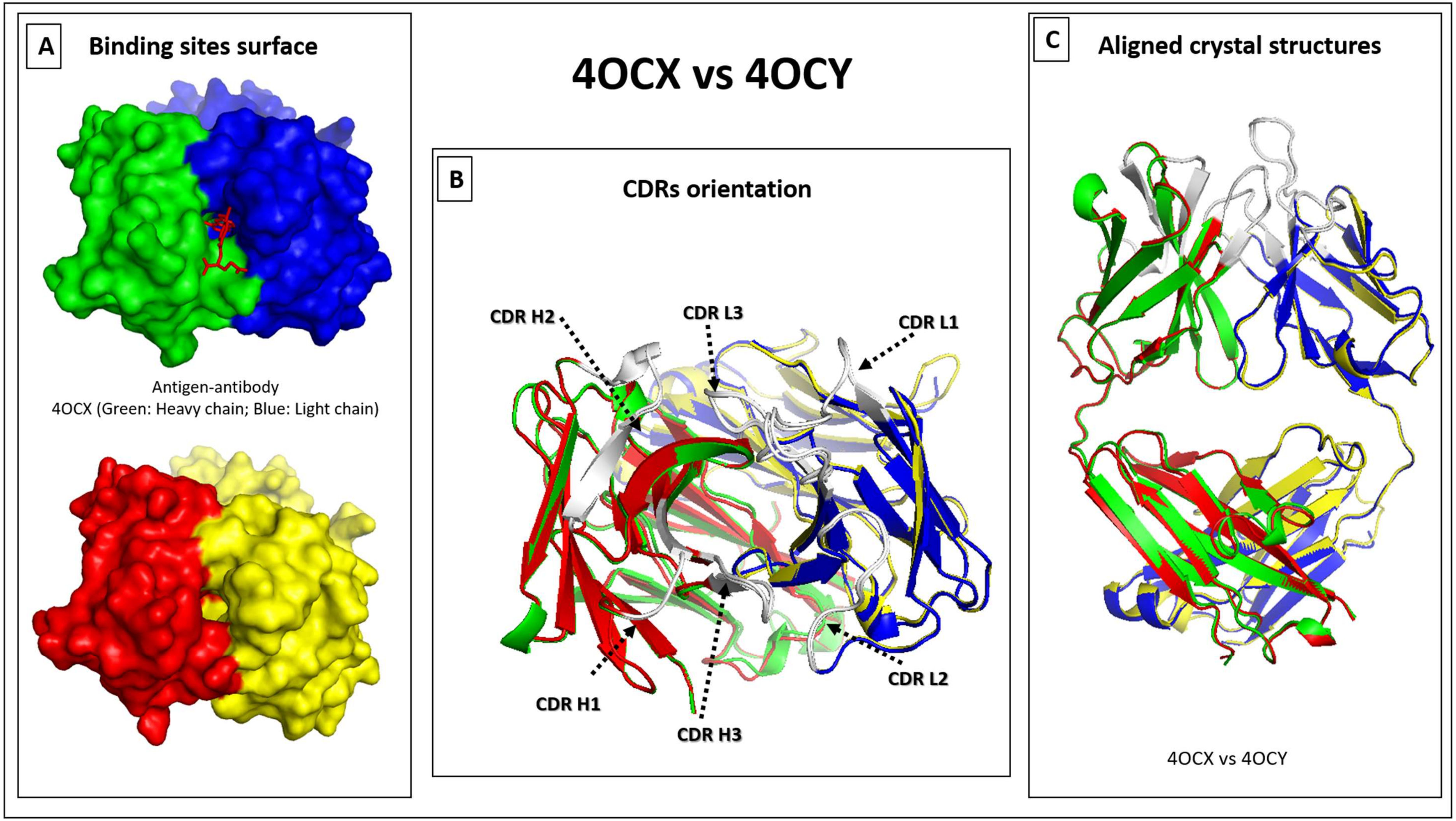

4OCX vs 4OCY

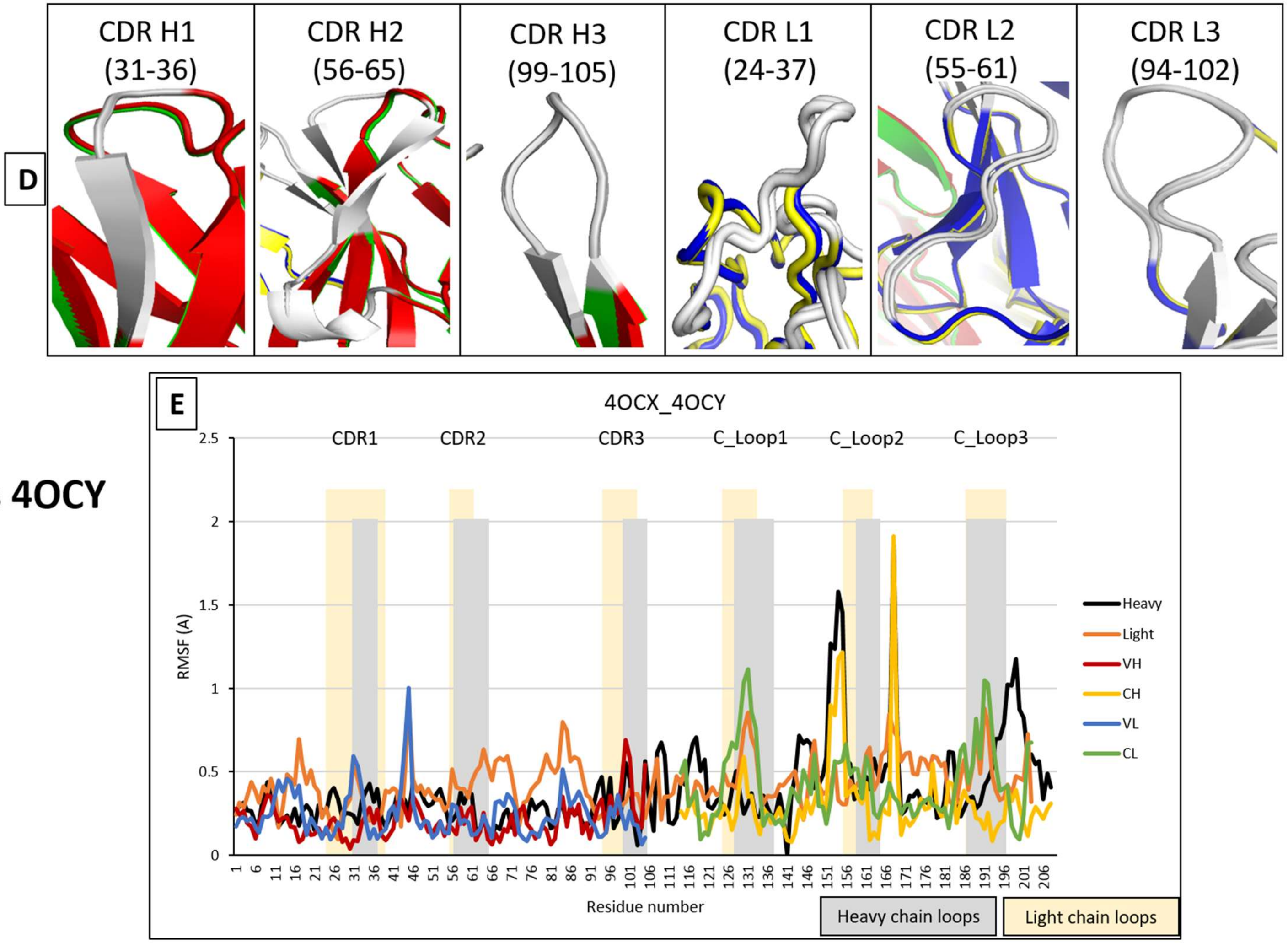
D
CDR H1 (31-36)
CDR H2 (56-65)
CDR H3 (99-105)
CDR L1 (24-37)
CDR L2 (55-61)
CDR L3 (94-102)
E
4OCX_4OCY
CDR1
CDR2
CDR3
C_Loop1
C_Loop2
C_Loop3
RMSF (A)
Residue number
Heavy
Light
VH
CH
VL
CL
Heavy chain loops
Light chain loops

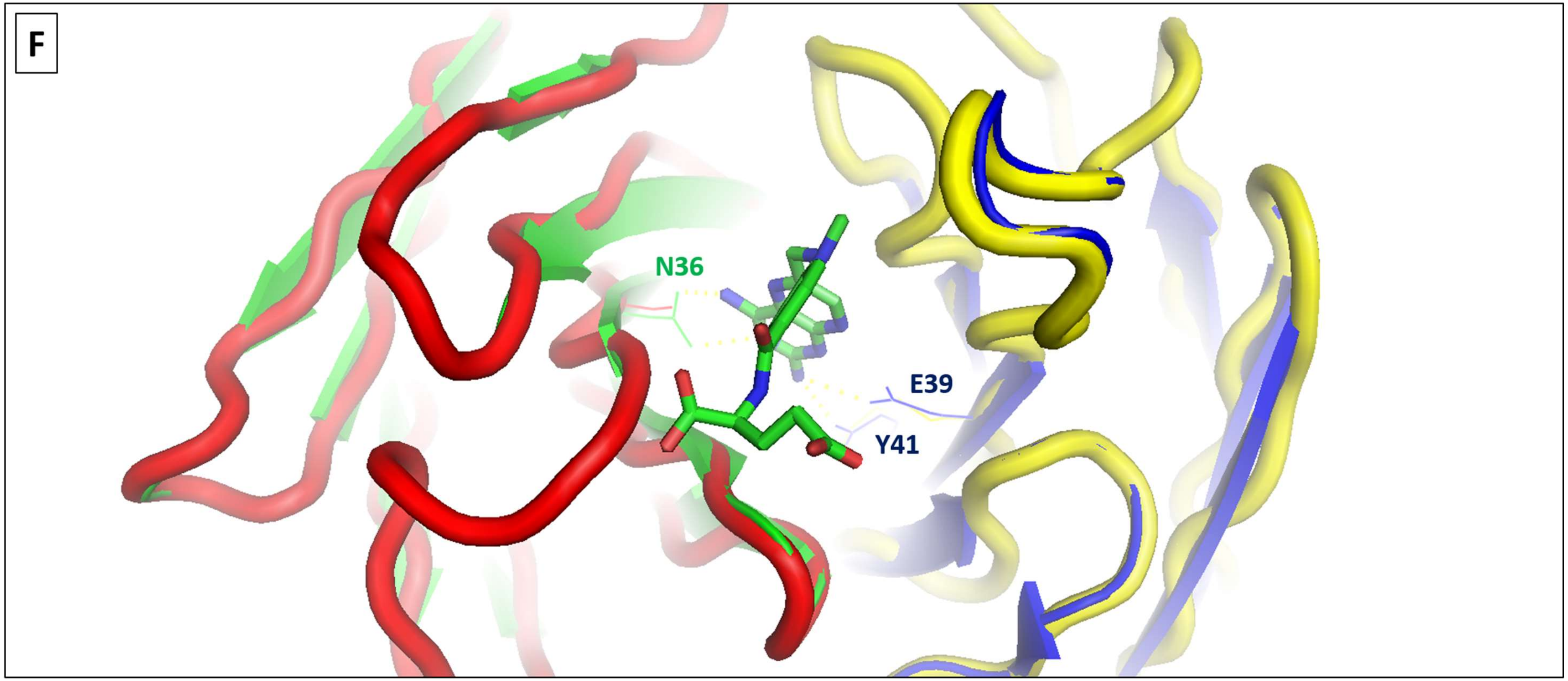
F
N36
E39
Y41

S.2.18 (1I9J vs 1I9I), mouse:

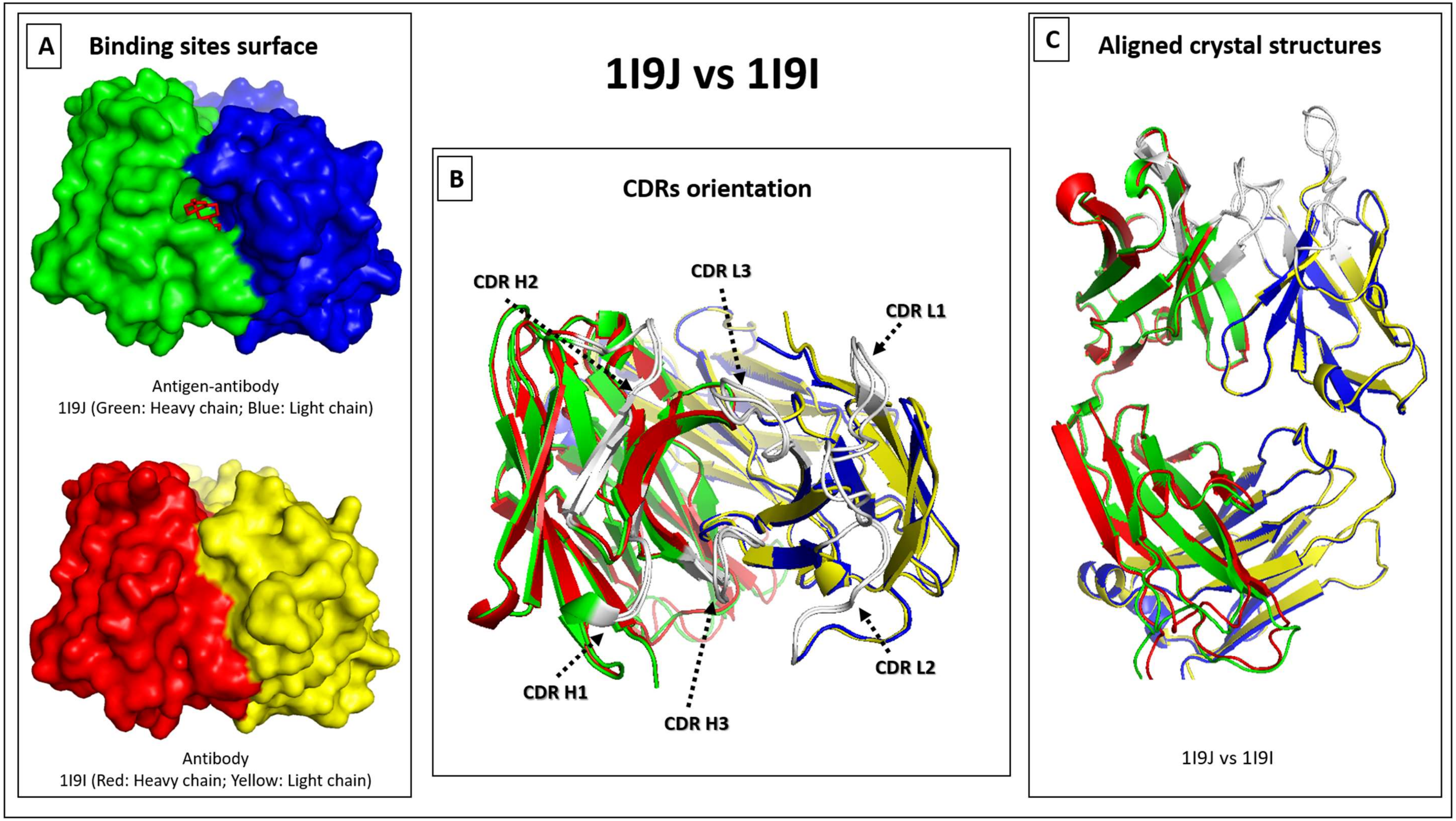

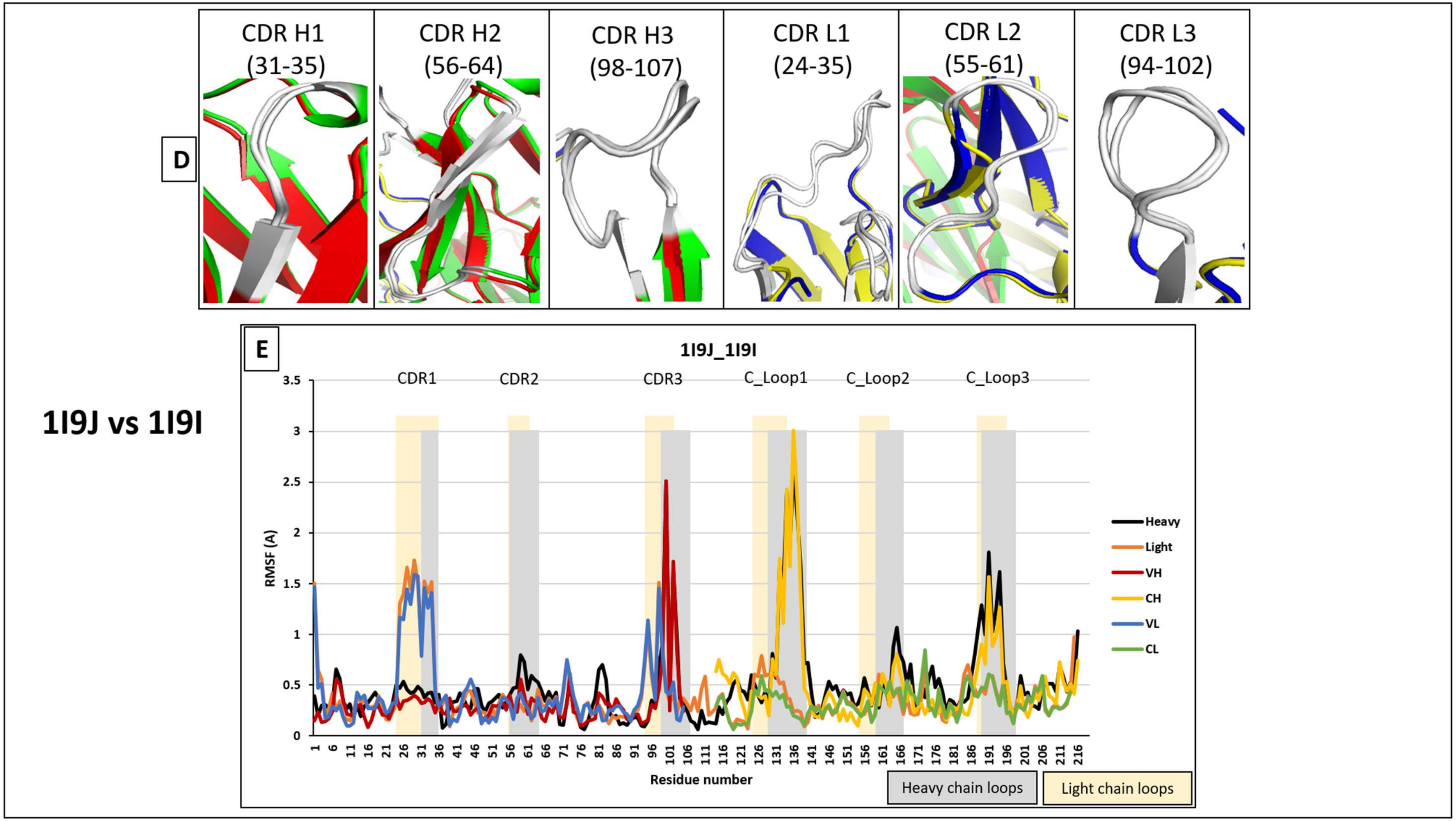
1I9J vs 1I9I
D
CDR H1 (31-35)
CDR H2 (56-64)
CDR H3 (98-107)
CDR L1 (24-35)
CDR L2 (55-61)
CDR L3 (94-102)
E
1I9J_1I9I
CDR1
CDR2
CDR3
C_Loop1
C_Loop2
C_Loop3
RMSF (A)
Residue number
Heavy
Light
VH
CH
VL
CL
Heavy chain loops
Light chain loops

S.2.19 (3LS4 vs 3LS5), mouse:

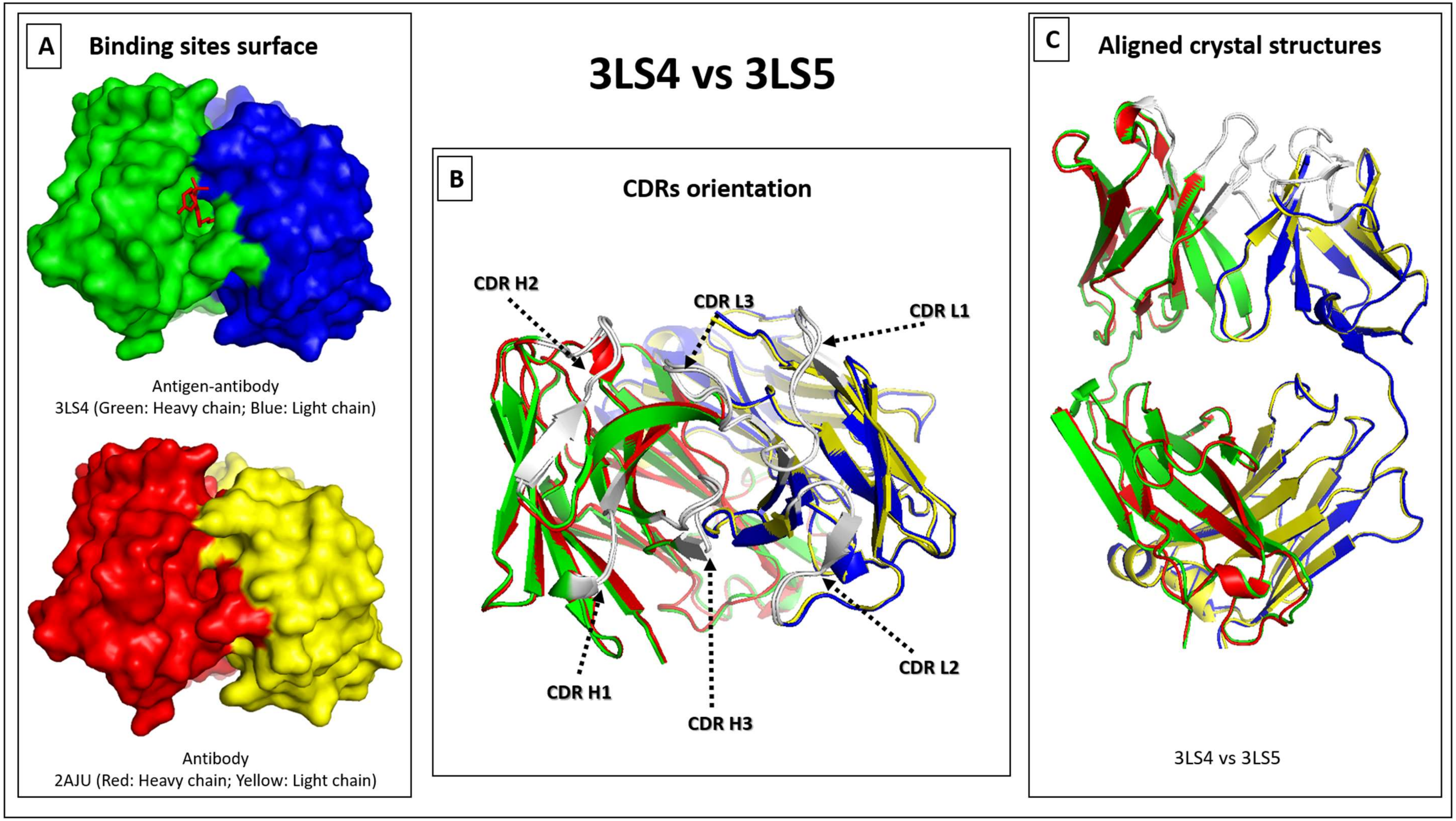

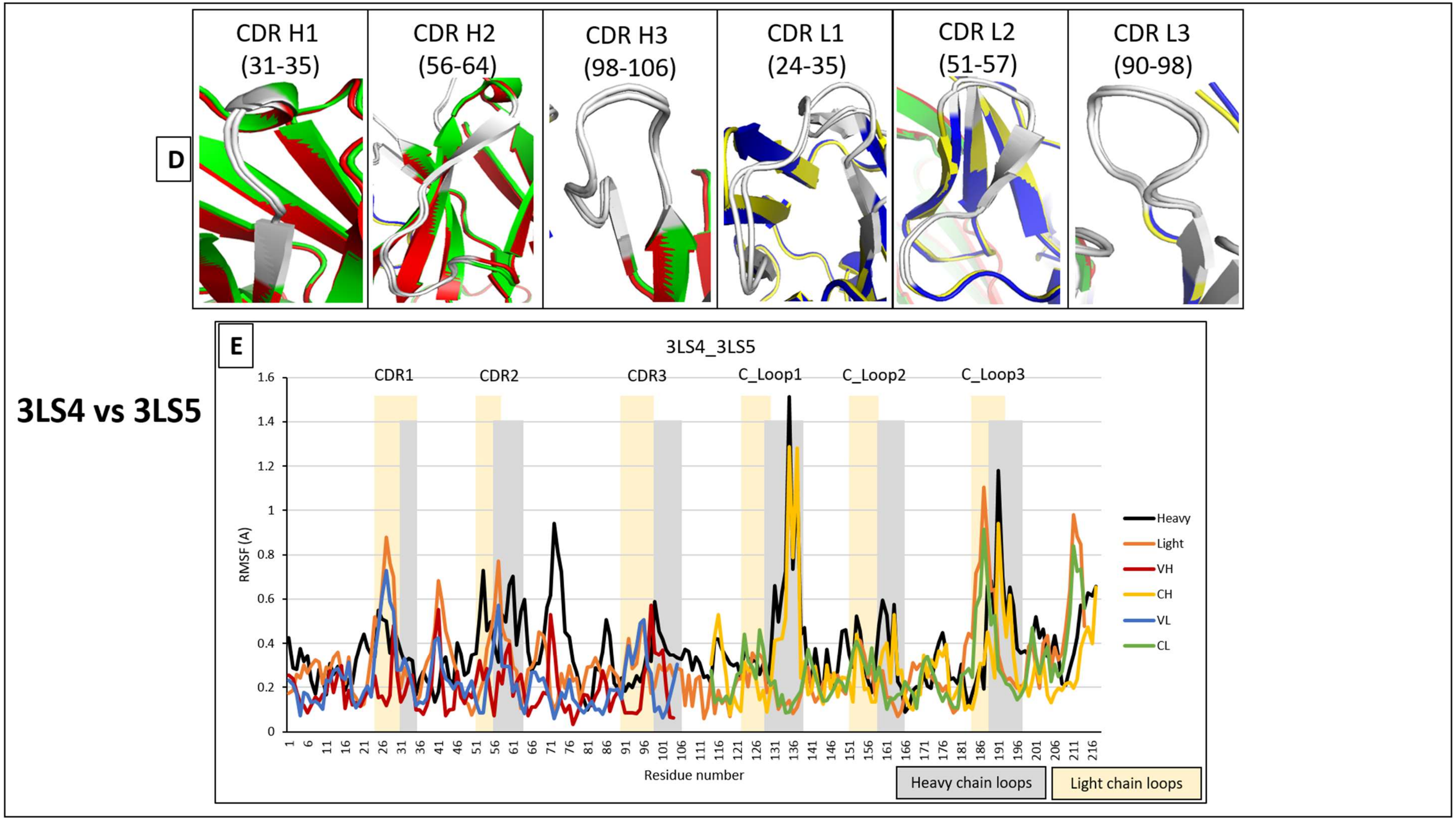
3LS4 vs 3LS5
D
CDR H1 (31-35)
CDR H2 (56-64)
CDR H3 (98-106)
CDR L1 (24-35)
CDR L2 (51-57)
CDR L3 (90-98)
E
3LS4_3LS5
CDR1
CDR2
CDR3
C_Loop1
C_Loop2
C_Loop3
RMSF (A)
Residue number
Heavy
Light
VH
CH
VL
CL
Heavy chain loops
Light chain loops

S.2.20 (1FL6 vs 1FL5), human:

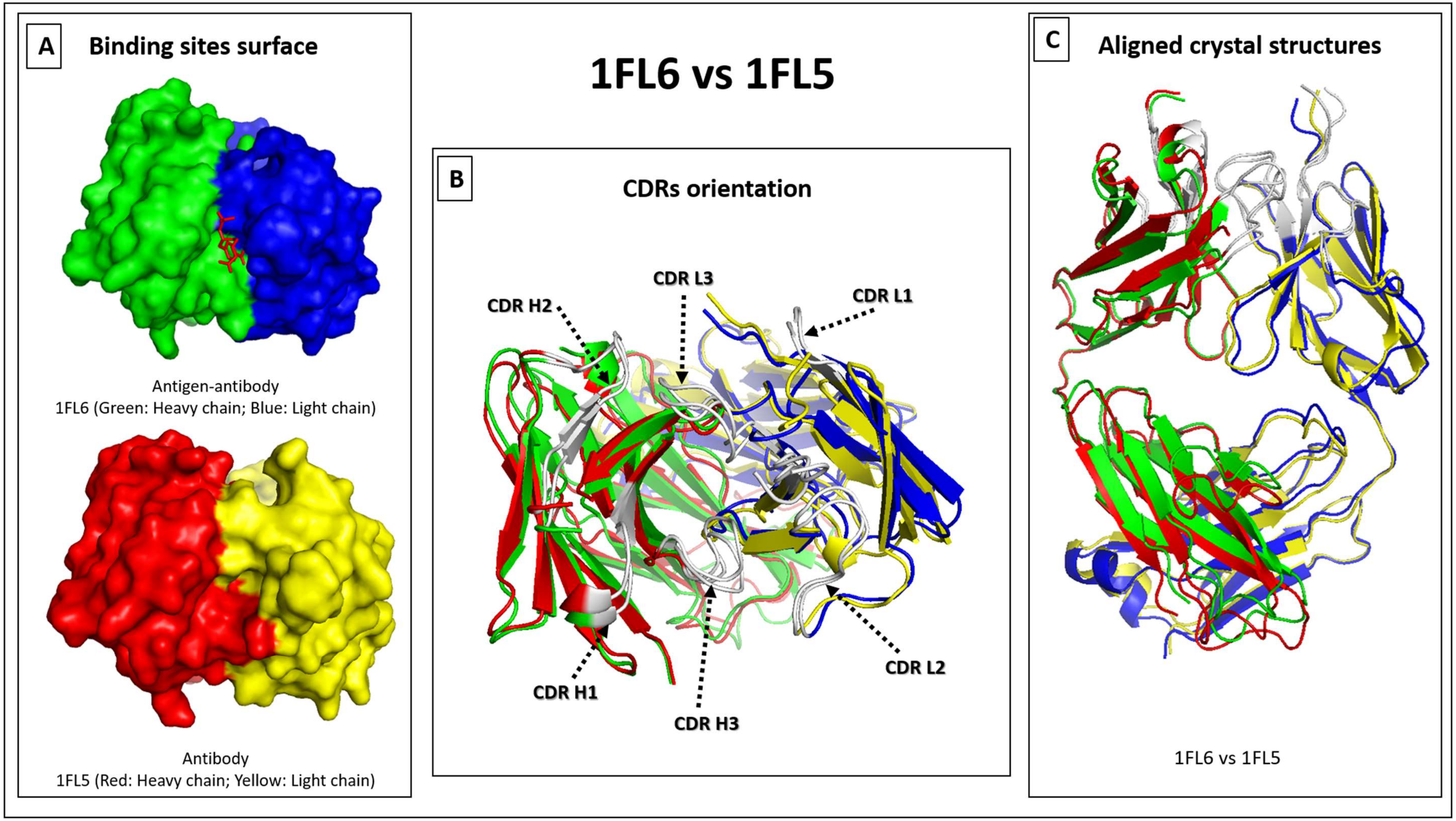

1FL6 vs 1FL5

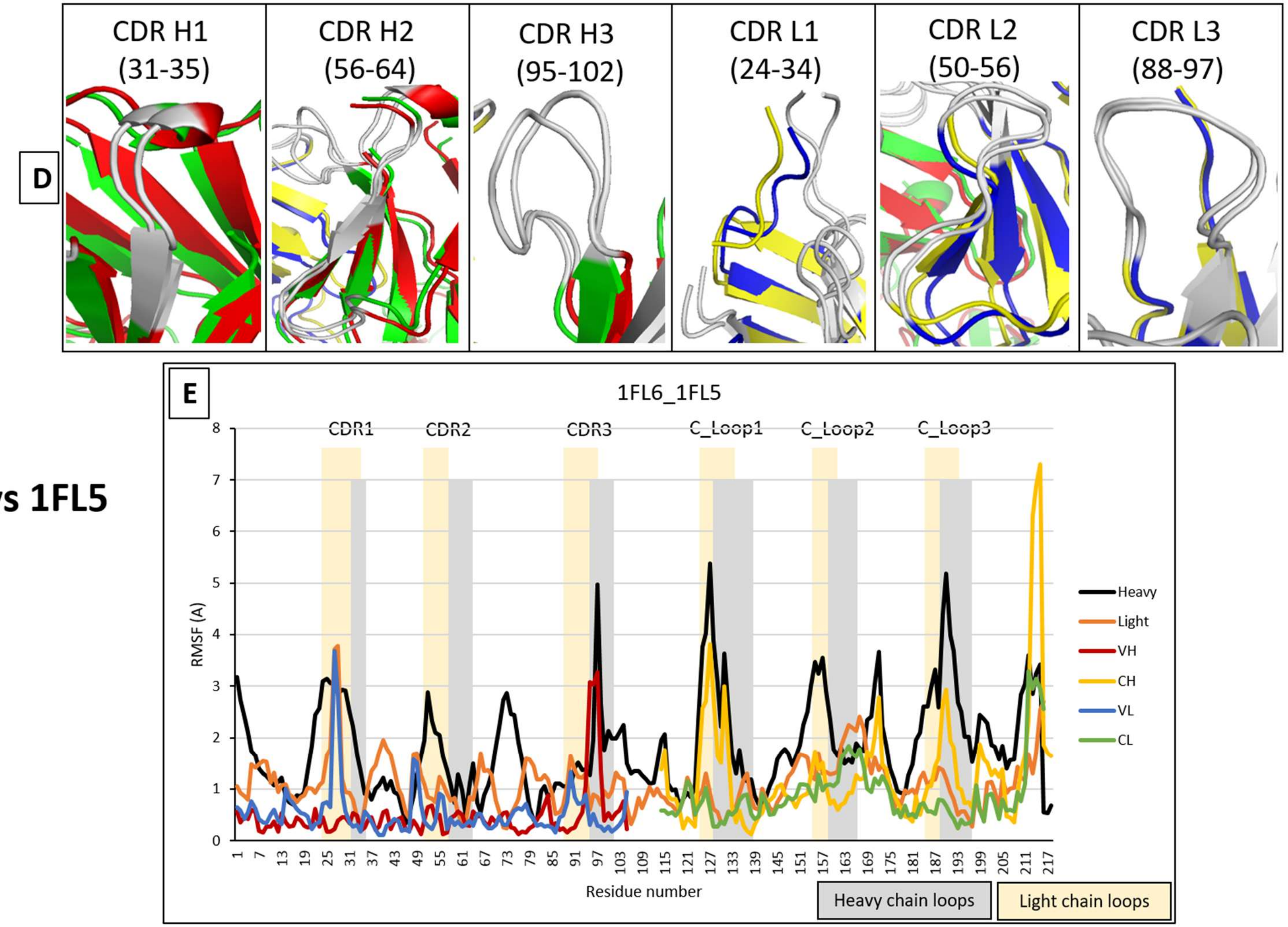

D
CDR H1 (31-35)
CDR H2 (56-64)
CDR H3 (95-102)
CDR L1 (24-34)
CDR L2 (50-56)
CDR L3 (88-97)
E
1FL6_1FL5
CDR1
CDR2
CDR3
C_Loop1
C_Loop2
C_Loop3
RMSF (A)
Residue number
Heavy
Light
VH
CH
VL
CL
Heavy chain loops
Light chain loops

S.2.21 (1C5C vs 1C5B), human:

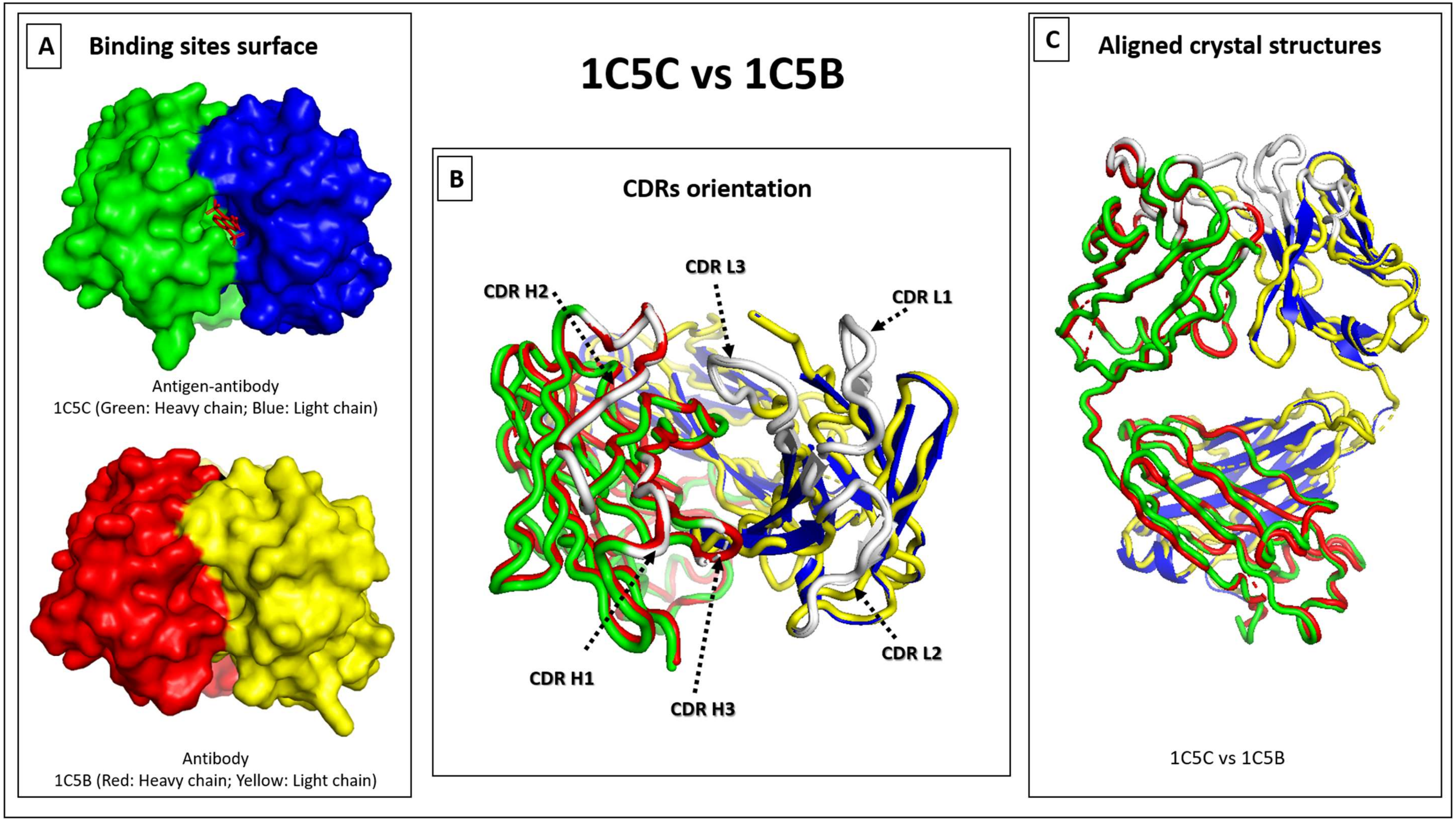

1C5C vs 1C5B

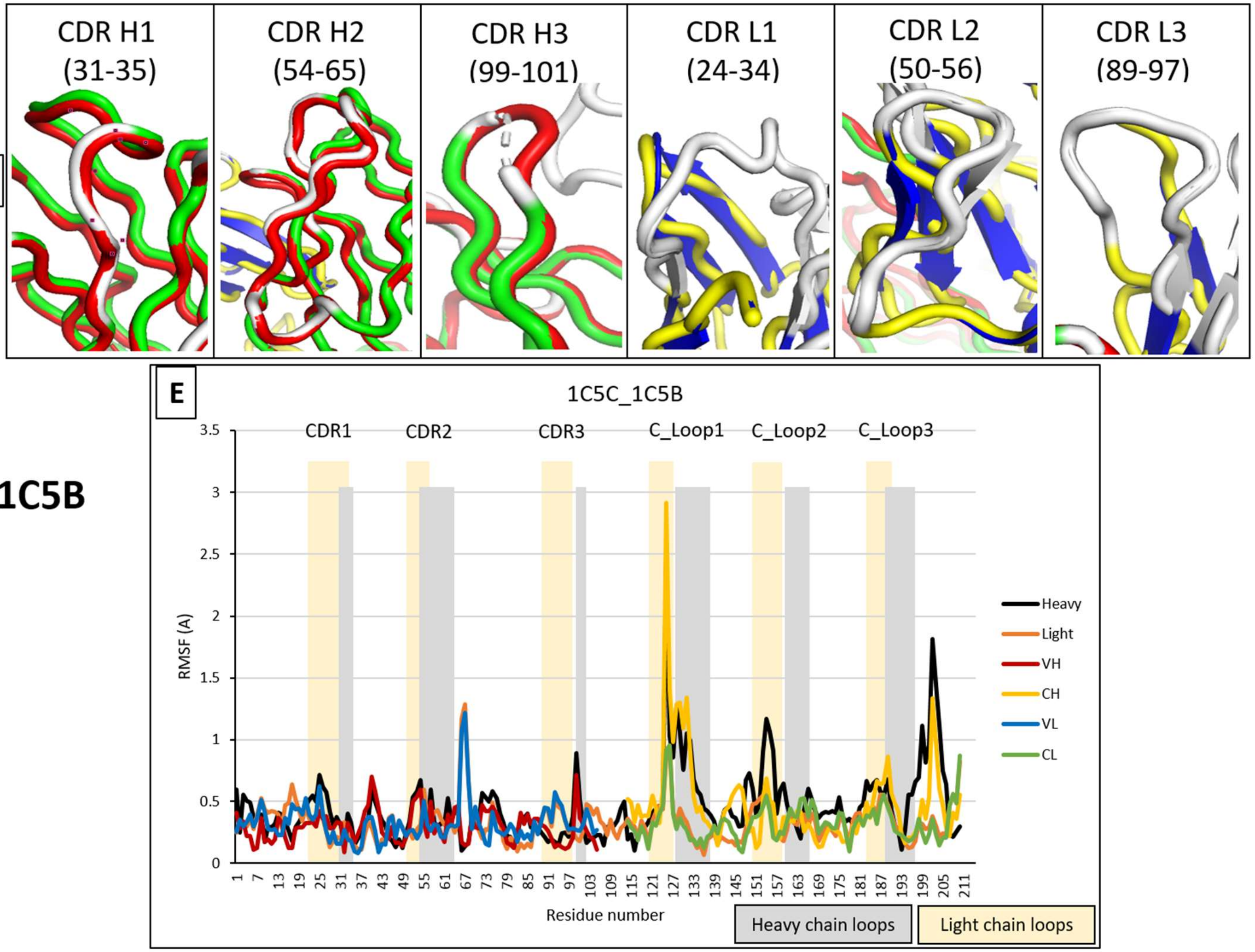

D
CDR H1 (31-35)
CDR H2 (54-65)
CDR H3 (99-101)
CDR L1 (24-34)
CDR L2 (50-56)
CDR L3 (89-97)
E
1C5C_1C5B
CDR1
CDR2
CDR3
C_Loop1
C_Loop2
C_Loop3
RMSF (A)
Residue number
Heavy
Light
VH
CH
VL
CL
Heavy chain loops
Light chain loops

S.2.22 (1D6V vs 1D5B), human:

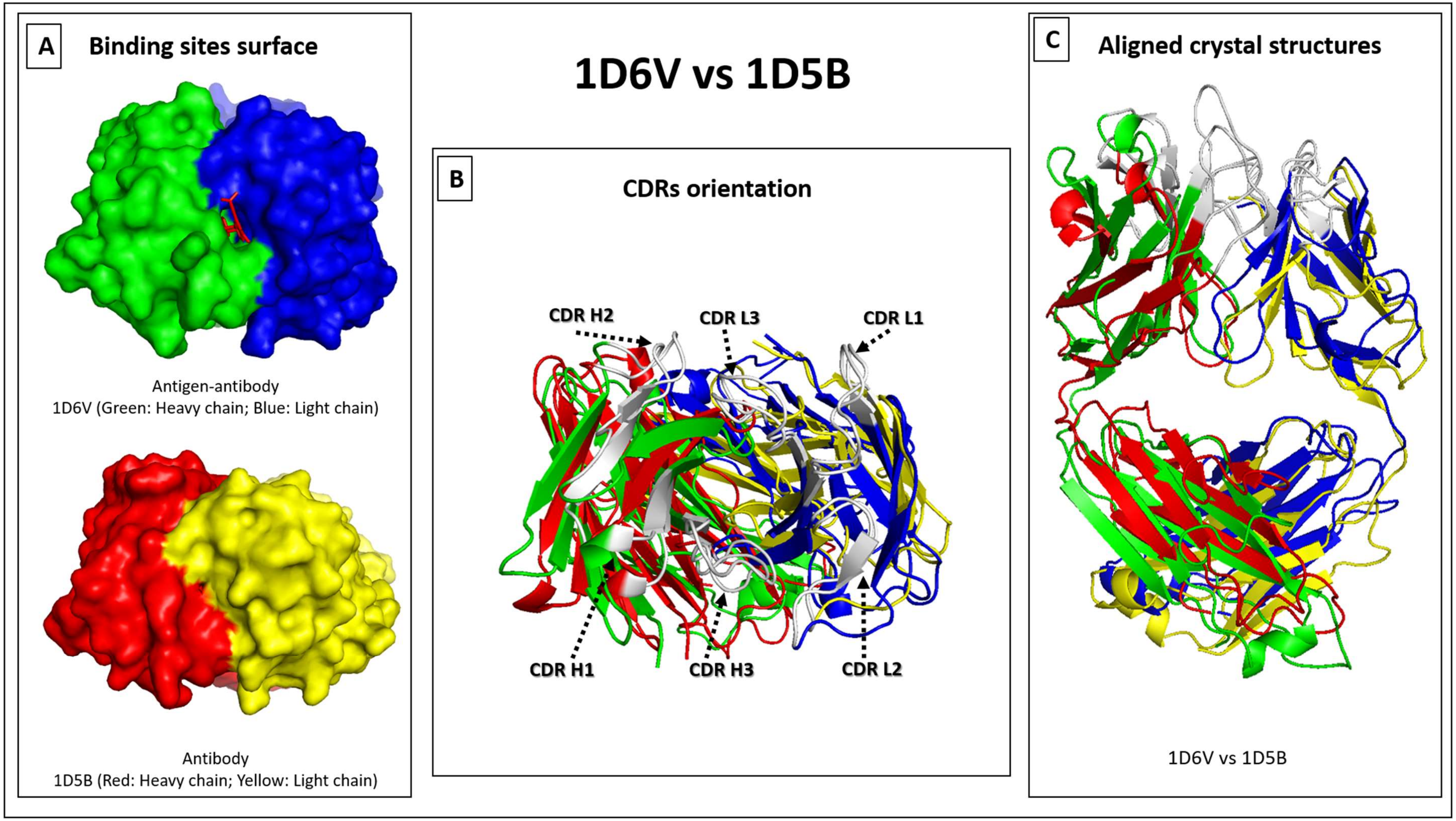

1D6V vs 1D5B

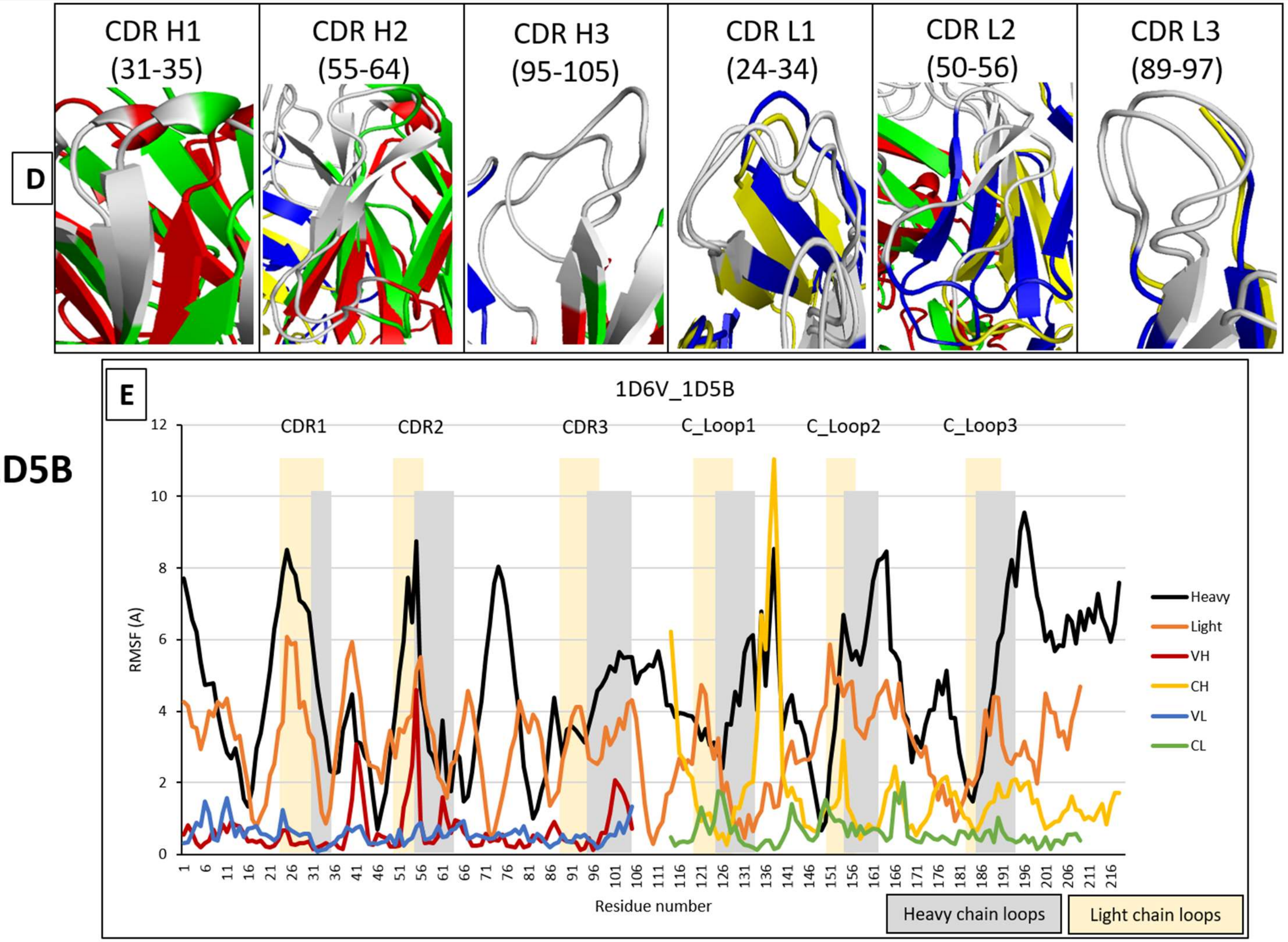
D
CDR H1 (31-35)
CDR H2 (55-64)
CDR H3 (95-105)
CDR L1 (24-34)
CDR L2 (50-56)
CDR L3 (89-97)
E
1D6V_1D5B
CDR1
CDR2
CDR3
C_Loop1
C_Loop2
C_Loop3
RMSF (A)
Residue number
Heavy
Light
VH
CH
VL
CL
Heavy chain loops
Light chain loops

S.2.23 (1AJ7 vs 2RCS), human:

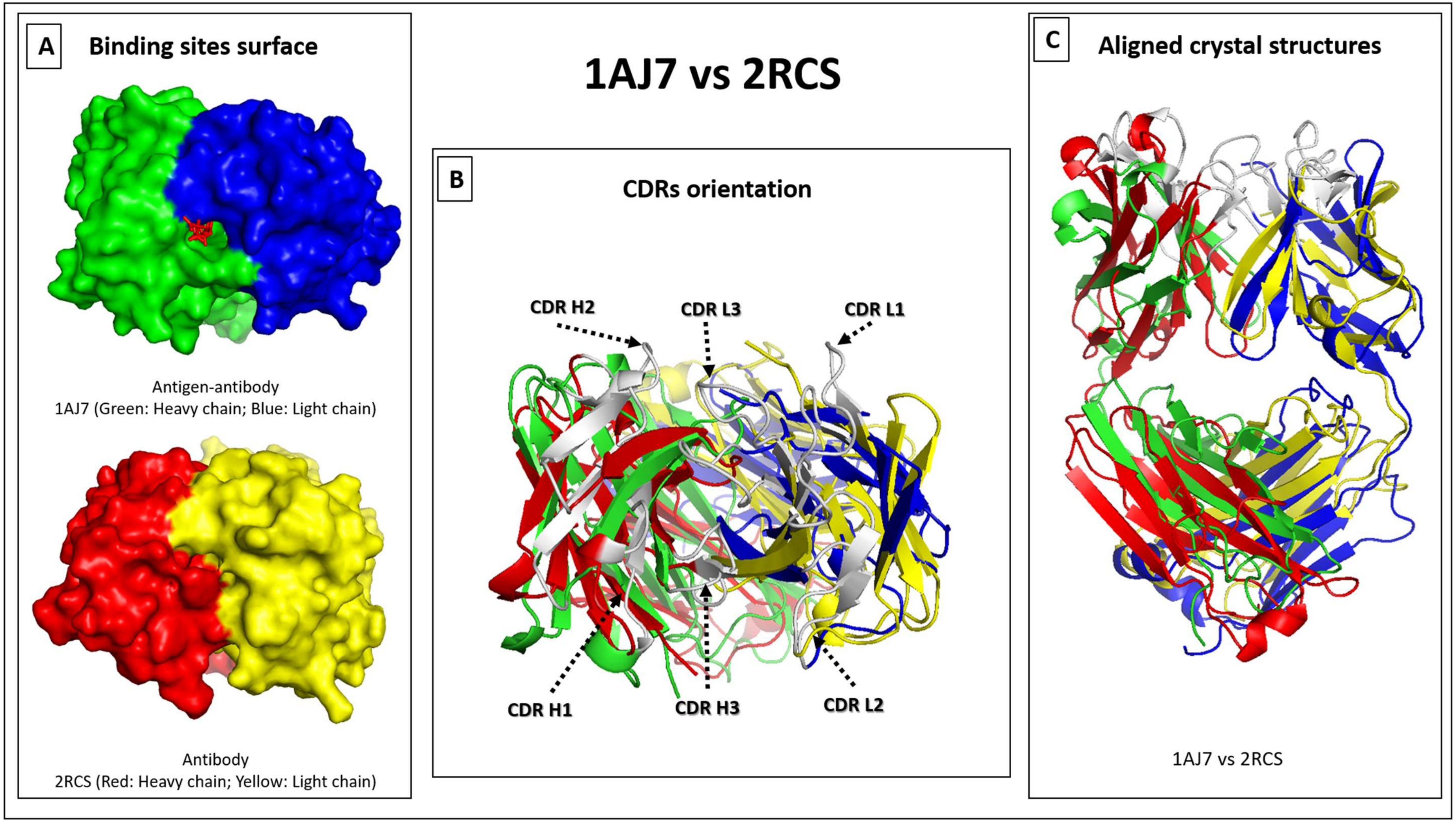

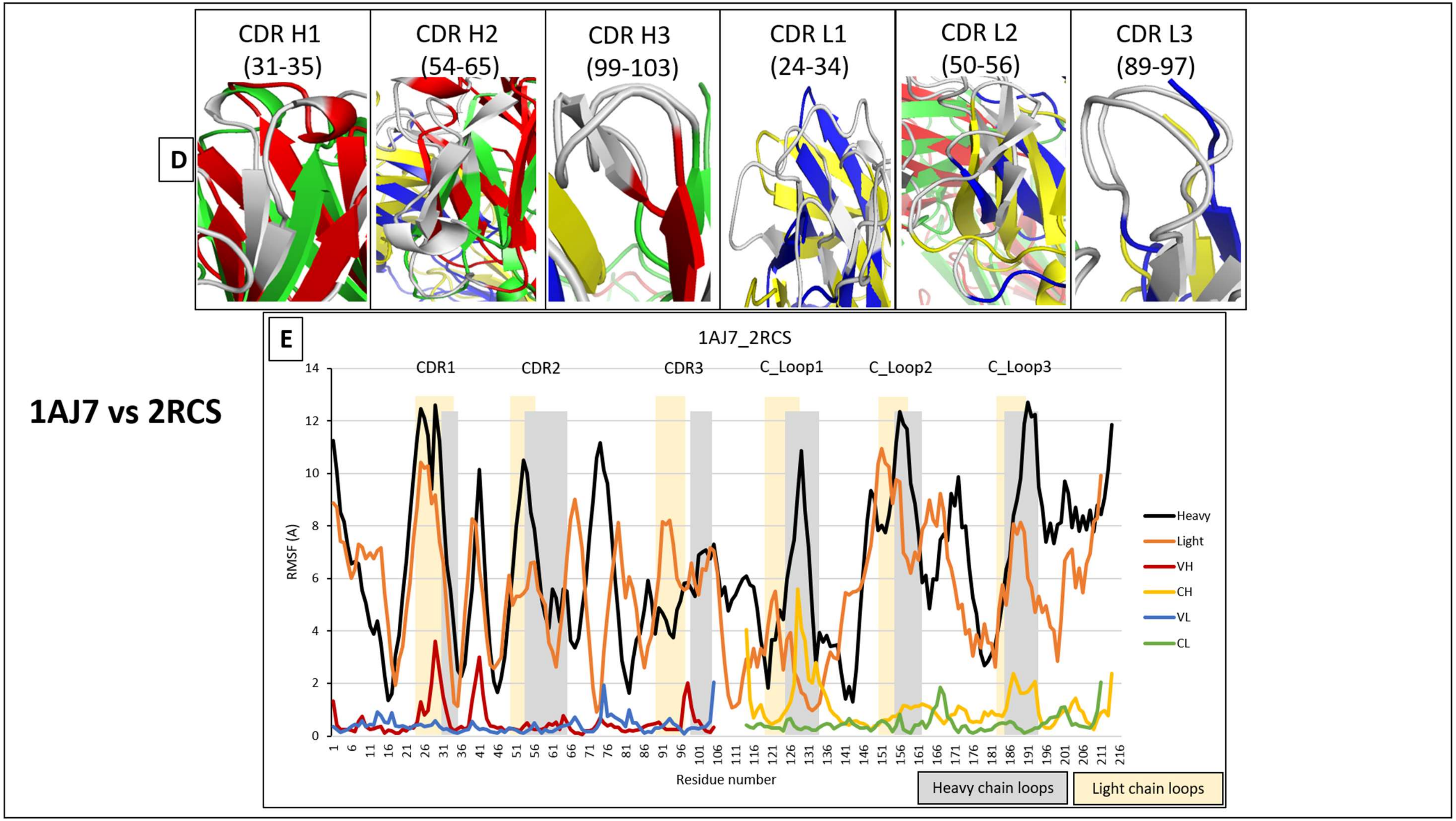

D
CDR H1 (31-35)
CDR H2 (54-65)
CDR H3 (99-103)
CDR L1 (24-34)
CDR L2 (50-56)
CDR L3 (89-97)
1AJ7 vs 2RCS
E
1AJ7_2RCS
CDR1
CDR2
CDR3
C_Loop1
C_Loop2
C_Loop3
RMSF (A)
Residue number
Heavy
Light
VH
CH
VL
CL
Heavy chain loops
Light chain loops

Supplementary S3: Sequences Alignment

The antibodies' sequences were acquired from the Protein Data Bank (PDB), and were analysed using BioEdit Sequence Alignment Editor, version 7.2.5*.A total of 11 human antibodies against protein antigens (4 mouse antibodies against protein antigens). Each sequence was split to heavy and light (lambda or kappa) chains, and then aligned. ClustalW Multiple alignment was used to align sequences of the same formats. Conserved positions of the aligned sequences to the first sequence were viewed by plotting identities a dot.

* Hall T. BioEdit: a user-friendly biological sequence alignment editor and analysis program for Windows 95/98/NT. Nucl Acids Symp Ser.1999;95–98.

Table S3.1 Hapten_Human_Heavy chain_8 sequences

This group represent four human (1AJ7, 2RCS, 1D6V, and 1D5B) and four chimeric mouse/human (1FL6, 1FL5, 1C5C, and 1C5B) antibodies. All constant domains (CH1) are of human IgG1 type.

```
                                   10        20        30        40        50        60        70        80        90       100
                           ....|....|....|....|....|....|....|....|....|....|....|....|....|....|....|....|....|....|....|....|
1AJ7:H|PDBID|CHAIN|SEQUENCE QVQLQQSGAELVKPGASVKLSCTASGFNIKDTYMHWVKQRPEQGLEWIGRID-PANG-NTKYDPKFQGKATITADTSSNTAYLQLSSLTSEDTAVYYCAS
2RCS:H|PDBID|CHAIN|SEQUENCE ....................................................-....-..........................................
1C5B:H|PDBID|CHAIN|SEQUENCE ....LEP.T.............R...YSFTSYW........G.......L..-.S..-R.NFND..KSR..L.V....S...M.........S.....VR
1C5C:H|PDBID|CHAIN|SEQUENCE ....LEP.T.............R...YSFTSYW........G.......L..-.S..-R.NFND..KSR..L.V....S...M.........S.....VR
1D5B:H|PDBID|CHAIN|SEQUENCE ...........M.......I..K.T.YTFSSFWIE......GH......E.L-.GS.-G.H.NE..K....F...K......M.........S......R
1D6V:H|PDBID|CHAIN|SEQUENCE ...........M.......I..K.T.YTFSSYWIE......GH......E.L-.GS.-S.N.NE..K....F..........M.........S......R
1FL5:H|PDBID|CHAIN|SEQUENCE ....VE..GG..Q..G.LR...AT...TFT.Y..S..R.P.GKA...L.F.RNK...YT.E.SASVK.RF..SR.N.QSIL...MNT.RA..S.T....R
1FL6:H|PDBID|CHAIN|SEQUENCE ....VE..GG..Q..G.LR...AT...TFT.Y..S..R.P.GKA...L.F.RNK...YT.E.SASVK.RF..SR.N.QSIL...MNT.RA..S.T....R

                                  110       120       130       140       150       160       170       180       190       200
                           ....|....|....|....|....|....|....|....|....|....|....|....|....|....|....|....|....|....|....|....|
1AJ7:H|PDBID|CHAIN|SEQUENCE ------YYGIYWGQGTTLTVSSASTKGPSVFPLAPSSKSTSGGTAALGCLVKDYFPEPVTVSWNSGALTSGVHTFPAVLQSSGLYSLSSVVTVPSSSLGT
2RCS:H|PDBID|CHAIN|SEQUENCE ------..............................................................................................
1C5B:H|PDBID|CHAIN|SEQUENCE --------IA......LV..................................................................................
1C5C:H|PDBID|CHAIN|SEQUENCE --------IA......LV..................................................................................
1D5B:H|PDBID|CHAIN|SEQUENCE GHSYYF.D.D......SV..................................................................................
1D6V:H|PDBID|CHAIN|SEQUENCE GHSYYF.D.D......SV..................................................................................
1FL5:H|PDBID|CHAIN|SEQUENCE DGS---.AMD......SV..................................................................................
1FL6:H|PDBID|CHAIN|SEQUENCE DGS---.AMD......SV..................................................................................

                                  210       220
                           ....|....|....|....|....|
1AJ7:H|PDBID|CHAIN|SEQUENCE QTYICNVNHKPSNTKVDKKVEPKSC
2RCS:H|PDBID|CHAIN|SEQUENCE .........................
1C5B:H|PDBID|CHAIN|SEQUENCE .........................
1C5C:H|PDBID|CHAIN|SEQUENCE .........................
1D5B:H|PDBID|CHAIN|SEQUENCE .......................--
1D6V:H|PDBID|CHAIN|SEQUENCE .......................--
1FL5:H|PDBID|CHAIN|SEQUENCE ......................---
1FL6:H|PDBID|CHAIN|SEQUENCE ......................---
```

Table S3.2 Hapten_Human_Light chain_Kappa_8 sequences

This group represent four human (1AJ7, 2RCS, 1D6V, and 1D5B) and four chimeric mouse/human (1FL6, 1FL5, 1C5C, and 1C5B) antibodies. All constant domains (Ck) are of human kappa type.

```
                                    10        20        30        40        50        60        70        80        90       100
                             ....|....|....|....|....|....|....|....|....|....|....|....|....|....|....|....|....|....|....|....|
1AJ7:L|PDBID|CHAIN|SEQUENCE  DIQMTQSPSSLSASLGERVSLTCRASQEISG-----YLSWLQQKPDGTIKRLIYAASTLDSGVPKRFSGSRSGSDYSLTISSLESEDFADYYCLQYASYP
2RCS:L|PDBID|CHAIN|SEQUENCE  ...............................-----................................................................
1C5B:L|PDBID|CHAIN|SEQUENCE  E..L....................T......-----..................D.TK....A...................................F.
1C5C:L|PDBID|CHAIN|SEQUENCE  E..L....................T......-----..................D.TK....A...................................F.
1D5B:L|PDBID|CHAIN|SEQUENCE  ..K.......MY.......TI..K...D.NS-----..N.F....GKSP.T...RTNR.VD...S.....G..Q..........Y..MGI......DEF.
1D6V:L|PDBID|CHAIN|SEQUENCE  ..K.......MY.......TI..K...D.NS-----....F....GKSP.T...R.NR.VD...S.....G..Q..........Y..MGI......DEF.
1FL5:L|PDBID|CHAIN|SEQUENCE  ELV...T.L..PV...DQA.IS..S..S.VHSNGNT..E.YL...GQSP.L...KV.NRF....D.....G..T.FT.K..RV.A..LGV...F.GSHV.
1FL6:L|PDBID|CHAIN|SEQUENCE  ELV...T.L..PV...DQA.IS..S..S.VHSNGNT..E.YL...GQSP.L...KV.NRF....D.....G..T.FT.K..RV.A..LGV...F.GSHV.

                                   110       120       130       140       150       160       170       180       190       200
                             ....|....|....|....|....|....|....|....|....|....|....|....|....|....|....|....|....|....|....|....|
1AJ7:L|PDBID|CHAIN|SEQUENCE  RTFGGGTKVEIKRTVAAPSVFIFPPSDEQLKSGTASVVCLLNNFYPREAKVQWKVDNALQSGNSQESVTEQDSKDSTYSLSSTLTLSKADYEKHKVYACE
2RCS:L|PDBID|CHAIN|SEQUENCE  ....................................................................................................
1C5B:L|PDBID|CHAIN|SEQUENCE  ........L...........................................................................................
1C5C:L|PDBID|CHAIN|SEQUENCE  ........L...........................................................................................
1D5B:L|PDBID|CHAIN|SEQUENCE  Y...S...L...........................................................................................
1D6V:L|PDBID|CHAIN|SEQUENCE  Y...S...L...........................................................................................
1FL5:L|PDBID|CHAIN|SEQUENCE  ........L...........................................................................................
1FL6:L|PDBID|CHAIN|SEQUENCE  ........L...........................................................................................

                                   210
                             ....|....|....|....
1AJ7:L|PDBID|CHAIN|SEQUENCE  VTHQGLSSPVTKSFNRGEC
2RCS:L|PDBID|CHAIN|SEQUENCE  ...................
1C5B:L|PDBID|CHAIN|SEQUENCE  ...................
1C5C:L|PDBID|CHAIN|SEQUENCE  ...................
1D5B:L|PDBID|CHAIN|SEQUENCE  ................---
1D6V:L|PDBID|CHAIN|SEQUENCE  ................---
1FL5:L|PDBID|CHAIN|SEQUENCE  .................--
1FL6:L|PDBID|CHAIN|SEQUENCE  .................--
```

Table S3.3 Hapten_Mouse_Heavy chain_32 sequences

The 32 antibodies can be classified as 11 IgG1 (1Q72, 1QYG, 1RFD, 1JNN, 1JNL, 1KEL, 1KEM, 1NGP, 1NGQ, 3LS4, and 3LS5), 9 IgG2a (2AJS, 2AJV, 2AJX, 2AJY, 2AJZ, 2AK1, 2AJU, 4OCX, and 4OCY), 10 IgG2b (3CFB, 3CFC, 3CFD, 3CFE, 1UB5, 1UB6, 2CGR, 1CGS, 1I9J, and 1I9I), and 2 IgG (1Q0Y and 1Q0X).

```
                                       10        20        30        40        50        60        70        80        90       100
                               ....|....|....|....|....|....|....|....|....|....|....|....|....|....|....|....|....|....|....|....|
1CGS:H|PDBID|CHAIN|SEQUENCE    RVQLLESGAELMKPGASVQISCKATGYTFS-EYWIEWVKERPGHGLEWIGEILPGSGR--TNYREKFKGKATFTADTSSNTAYMQLSSLTSEDSAVYYCT
2CGR:H|PDBID|CHAIN|SEQUENCE    ..............................-...........................--........................................
1I9I:H|PDBID|CHAIN|SEQUENCE    E.K.V...GG.V...G.LKL..A.S.F...-T.ALS..RQTADKR...VAS.VS.GN---.Y.SGSV..RF.ISR.IAR.IL.L.M...R...T.M...A
1I9J:H|PDBID|CHAIN|SEQUENCE    E.K.V...GG.V...G.LKL..A.S.F...-T.ALS..RQTADKR...VAS.VS.GN---.Y.SGSV..RF.ISR.IAR.IL.L.M...R...T.M...A
1JNL:H|PDBID|CHAIN|SEQUENCE    E...QQ.....V......RL..S.S.FNIK-DTYMF...Q..EQ..D...R.N.AN.I--SK.DPR.Q....L..........L..DN.....T.....A
1JNN:H|PDBID|CHAIN|SEQUENCE    E...QQ.....V......RL..S.S.FNIK-DTYMF...Q..EQ..D...R.N.AN.I--SK.DPR.Q....L..........L..DN.....T.....A
1KEL:H|PDBID|CHAIN|SEQUENCE    E.K.V...GG.GQ..G.LRL..ATS.F..T-D.YFN.ARQP..KA...L.F.RNKAKGYT.E.SASV..RF.ISR.N.QGIL.L.MNT.RA....T...A
1KEM:H|PDBID|CHAIN|SEQUENCE    E.K.V...GG.GQ..G.LRL..ATS.F..T-D.YFN.ARQP..KA...L.F.RNKAKGYT.E.SASV..RF.ISR.N.QGIL.L.MNT.RA....T...A
1NGP:H|PDBID|CHAIN|SEQUENCE    Q...QQP....V......KL....S....T-S..MH...Q...R......R.D.N..G--.K.N....S...L.V.KP.S...................A
1NGQ:H|PDBID|CHAIN|SEQUENCE    Q...QQP....V......KL....S....T-S..MH...Q...R......R.D.N..G--.K.N....S...L.V.KP.S...................A
1Q0X:H|PDBID|CHAIN|SEQUENCE    E...QQ............K...........-S.......Q.................D--.IFN...................................A
1Q0Y:H|PDBID|CHAIN|SEQUENCE    E...QQ............K...........-S.......Q.................D--.IFN...................................A
1Q72:H|PDBID|CHAIN|SEQUENCE    E.T.Q...GG.VQ..G.MKL..A.S.F...-DA.VD..RQS..K....VA..RNKANNHA.K.T.SV..RF.ISR.D.KSSV.L.MN..RA..TGI....
1QYG:H|PDBID|CHAIN|SEQUENCE    E.T.Q...GG.VQ..G.MKL..A.S.F...-DA.VD..RQS..K....VA..RNKANNHA.K.T.SV..RF.ISR.D.KSSV.L.MN..RA..TGI....
1RFD:H|PDBID|CHAIN|SEQUENCE    E.T.Q...GG.VQ..G.MKL..A.S.F...-DA.VD..RQS..K....VA..RNKANNHA.K.T.SV..RF.ISR.D.KSSV.L.MN..RA..TGI....
1UB5:A|PDBID|CHAIN|SEQUENCE    E.K.....GG.V...G.LKL..T.S.I...-R.IMS..RQI.EKR...VAS.SS.GI---.Y.PDSVA.RF.ISR.NVR.IL.L.M...R...T.L...A
1UB6:H|PDBID|CHAIN|SEQUENCE    -AA.....GG.V...G.LKL..T.S.I...-R.IMS..RQI.EKR...VAS.SS.GI---.Y.PDSVA.RF.ISR.NVR.IL.L.M...R...T.L...A
2AJS:H|PDBID|CHAIN|SEQUENCE    E.K.S...PG.V..SQ.LSLT.TV...SITTN.AWT.IRQF..NK...M.Y.RSSVI---.R.NPSL.SRISI.Q...K.QFFL..N.V.T..T.T...A
2AJU:H|PDBID|CHAIN|SEQUENCE    E.K.S...PG.V..SQ.LSLT.TV...SITTN.AWT.IRQF..NK...M.Y.RSSVI---.R.NPSL.SRISI.Q...K.QFFL..N.V.T..T.T...A
2AJV:H|PDBID|CHAIN|SEQUENCE    E.K.S...PG.V..SQ.LSLT.TV...SITTN.AWT.IRQF..NK...M.Y.RSSVI---.R.NPSL.SRISI.Q...K.QFFL..N.V.T..T.T...A
2AJX:H|PDBID|CHAIN|SEQUENCE    E.K.S...PG.V..SQ.LSLT.TV...SITTN.AWT.IRQF..NK...M.Y.RSSVI---.R.NPSL.SRISI.Q...K.QFFL..N.V.T..T.T...A
2AJY:H|PDBID|CHAIN|SEQUENCE    E.K.S...PG.V..SQ.LSLT.TV...SITTN.AWT.IRQF..NK...M.Y.RSSVI---.R.NPSL.SRISI.Q...K.QFFL..N.V.T..T.T...A
2AJZ:H|PDBID|CHAIN|SEQUENCE    E.K.S...PG.V..SQ.LSLT.TV...SITTN.AWT.IRQF..NK...M.Y.RSSVI---.R.NPSL.SRISI.Q...K.QFFL..N.V.T..T.T...A
2AK1:H|PDBID|CHAIN|SEQUENCE    E.K.S...PG.V..SQ.LSLT.TV...SITTN.AWT.IRQF..NK...M.Y.RSSVI---.R.NPSL.SRISI.Q...K.QFFL..N.V.T..T.T...A
3CFB:H|PDBID|CHAIN|SEQUENCE    E.K.V...GG.V...G.LKL..T.S.I...-R.IMS..RQI.EKR...VAS.SS.GI---.Y.PDSV..RF.ISR.NVR.IL.L.M...R...T.L...A
3CFC:H|PDBID|CHAIN|SEQUENCE    E.K.V...GG.V...G.LKL..T.S.I...-R.IMS..RQI.EKR...VAS.SS.GI---.Y.PDSV..RF.ISR.NVR.IL.L.M...R...T.L...A
3CFD:H|PDBID|CHAIN|SEQUENCE    E...Q...PG.V..SQ.LSLT.TV...SITSD.AWN.LRQL..NK...M.Y.SYSGR---IR.NPSL.RRISI.R...K.QFFL..N.V.T..T.T...A
3CFE:H|PDBID|CHAIN|SEQUENCE    E...Q...PG.V..SQ.LSLT.TV...SITSD.AWN.LRQL..NK...M.Y.SYSGR---IR.NPSL.RRISI.R...K.QFFL..N.V.T..T.T...A
3LS4:H|PDBID|CHAIN|SEQUENCE    E.K.V...GG.V...G.LKL..A.S.F..N-N.VMV.LRQT.EKR...VAS.SR.GS---.Y.PDSV..RF.ISR.NAR.IL.L.M...R...T.M...V
3LS5:H|PDBID|CHAIN|SEQUENCE    E.K.V...GG.V...G.LKL..A.S.F..N-N.VMV.LRQT.EKR...VAS.SR.GS---.Y.PDSV..RF.ISR.NAR.IL.L.M...R...T.M...V
4OCX:H|PDBID|CHAIN|SEQUENCE    D...Q...PG.V..SQ.LSLT.TV..FSITSP.AWN.IRQF..NT...M.Y.SYRGS---.T.HPSL.SRISI.R...K.QFFL..N.V.T..T.T.F.S
4OCY:H|PDBID|CHAIN|SEQUENCE    D...Q...PG.V..SQ.LSLT.TV..FSITSP.AWN.IRQF..NT...M.Y.SYRGS---.T.HPSL.SRISI.R...K.QFFL..N.V.T..T.T.F.S

                                      110       120       130       140       150       160       170       180       190       200
                               ....|....|....|....|....|....|....|....|....|....|....|....|....|....|....|....|....|....|....|....|
1CGS:H|PDBID|CHAIN|SEQUENCE    R---GYSS---MDYWGQGTSVTVSAAKTTPPSVYPLAPG--CGDTTGSSVTLGCLVKGYFPESVTVTWNSGSLSSSVHTFPALLQSGLYTMSSSVTVPSS
2CGR:H|PDBID|CHAIN|SEQUENCE    .---....---............................--...........................................................
1I9I:H|PDBID|CHAIN|SEQUENCE    .---E.YGYVGLA......L...................SA--AQ.N.M.............P............G......V...D...L.........
1I9J:H|PDBID|CHAIN|SEQUENCE    .---E.YGYVGLA......L...................SA--AQ.N.M.............P............G......V...D...L.........
1JNL:H|PDBID|CHAIN|SEQUENCE    I--EKDLP------.....L....V..............--SAAQ.N.M.............P............G......V...D...L.........
1JNN:H|PDBID|CHAIN|SEQUENCE    I--EKDLP------.....L....V..............--SAAQ.N.M.............P............G......V...D...L.........
1KEL:H|PDBID|CHAIN|SEQUENCE    .---WG.--YA.............S..............SA--AQ.N.M.............P............G......V...D...L.........
1KEM:H|PDBID|CHAIN|SEQUENCE    .---WG.--YA.............S..............SA--AQ.N.M.............P............G......V...D...L.........
1NGP:H|PDBID|CHAIN|SEQUENCE    .--YD.YGSSYF.......TL...S..............--SAAQ.N.M.............P............G......V...D...L.........
1NGQ:H|PDBID|CHAIN|SEQUENCE    .--YD.YGSSYF.......TL...S..............--SAAQ.N.M.............P............G......V...D...L.........
```

```
1Q0X:H|PDBID|CHAIN|SEQUENCE  .WVLD.YG---.........L...S.S............GHHHHHHSAM.............P...V..K....TGT.....V.AAD...L......SA.
1Q0Y:H|PDBID|CHAIN|SEQUENCE  .WVLD.YG---.........L...S.S............GXXXXXXSAM.............P...V..K....TGT.....V.AAD...L......SA.
1Q72:H|PDBID|CHAIN|SEQUENCE  S---VPQLGRGFA......L......S............SGGAS.S..M.............P........A...G......V.NGD...L.........
1QYG:H|PDBID|CHAIN|SEQUENCE  S---VPQLGRGFA......L......S............SGGAS.S..M.............P........A...G......V...D...L.........
1RFD:H|PDBID|CHAIN|SEQUENCE  S---VPQLGRGFA......L......S............SGGAS.S..M.............P........A...G......V...D...L.........
1UB5:A|PDBID|CHAIN|SEQUENCE  .---.QG----RP......L....S..........A...CG--...................P........--G..........................
1UB6:H|PDBID|CHAIN|SEQUENCE  .---.QG----RP......L...............A...CG--............................--G..........................
2AJS:H|PDBID|CHAIN|SEQUENCE  .Y-DY.GNTG--............S..............TA--ALKS.M.............P............G......V...D...LT........
2AJU:H|PDBID|CHAIN|SEQUENCE  .Y-DY.GNTG--............S..............TA--ALKS.M.............P............G......V...D...LT........
2AJV:H|PDBID|CHAIN|SEQUENCE  .Y-DY.GNTG--............S..............TA--ALKS.M.............P............G......V...D...LT........
2AJX:H|PDBID|CHAIN|SEQUENCE  .Y-DY.GNTG--............S..............TA--ALKS.M.............P............G......V...D...LT........
2AJY:H|PDBID|CHAIN|SEQUENCE  .Y-DY.GNTG--............S..............TA--ALKS.M.............P............G......V...D...LT........
2AJZ:H|PDBID|CHAIN|SEQUENCE  .Y-DY.GNTG--............S..............TA--ALKS.M.............P............G......V...D...LT........
2AK1:H|PDBID|CHAIN|SEQUENCE  .Y-DY.GNTG--............S..............TA--ALKS.M.............P............G......V...D...LT........
3CFB:H|PDBID|CHAIN|SEQUENCE  .---.QG----RP......L....S..............CG--.........................................................
3CFC:H|PDBID|CHAIN|SEQUENCE  .---.QG----RP......L....S..............CG--.........................................................
3CFD:H|PDBID|CHAIN|SEQUENCE  .--SD.GNYGRG............S..............CG--........S................................................
3CFE:H|PDBID|CHAIN|SEQUENCE  .--SD.GNYGRG............S..............CG--........S................................................
3LS4:H|PDBID|CHAIN|SEQUENCE  .---.TT-IVAG.V..A..T....S..............SA--AQ.N.M.............P............G......V...D...L.........
3LS5:H|PDBID|CHAIN|SEQUENCE  .---.TT-IVAG.V..A..T....S..............SA--AQ.N.M.............P............G......V...D...L.........
4OCX:H|PDBID|CHAIN|SEQUENCE  ----S.GNYG--A.S....L...................SA--AQ.N.M.............P............G......V...D...L.........
4OCY:H|PDBID|CHAIN|SEQUENCE  ----S.GNYG--A.S....L...................SA--AQ.N.M.............P............G......V...D...L.........

                                  210       220
                             ....|....|....|....|....|....
1CGS:H|PDBID|CHAIN|SEQUENCE  TWPSQTVTCSVAHPASSTTVDKKLE----
2CGR:H|PDBID|CHAIN|SEQUENCE  .........................----
1I9I:H|PDBID|CHAIN|SEQUENCE  ....E....N........K....IVPRDC
1I9J:H|PDBID|CHAIN|SEQUENCE  ....E....N........K....IVPRDC
1JNL:H|PDBID|CHAIN|SEQUENCE  ....E....N........K....IVPRDC
1JNN:H|PDBID|CHAIN|SEQUENCE  ....E....N........K....IVPR--
1KEL:H|PDBID|CHAIN|SEQUENCE  PR..E....N........K....IVP---
1KEM:H|PDBID|CHAIN|SEQUENCE  PR..E....N........K....IVP---
1NGP:H|PDBID|CHAIN|SEQUENCE  P...E....N........K....IVPRDC
1NGQ:H|PDBID|CHAIN|SEQUENCE  P...E....N........K....IVPRDC
1Q0X:H|PDBID|CHAIN|SEQUENCE  S..G.S...N........K....IAPS--
1Q0Y:H|PDBID|CHAIN|SEQUENCE  S..G.S...N........K....IAPS--
1Q72:H|PDBID|CHAIN|SEQUENCE  .........N........Q....IVPK--
1QYG:H|PDBID|CHAIN|SEQUENCE  .........N........Q....IVPK--
1RFD:H|PDBID|CHAIN|SEQUENCE  .........N........Q....IVPK--
1UB5:A|PDBID|CHAIN|SEQUENCE  ....-....................----
1UB6:H|PDBID|CHAIN|SEQUENCE  ....-...........G........----
2AJS:H|PDBID|CHAIN|SEQUENCE  .........N........K....IVPR--
2AJU:H|PDBID|CHAIN|SEQUENCE  .........N........K....IVPR--
2AJV:H|PDBID|CHAIN|SEQUENCE  .........N........K....IVPR--
2AJX:H|PDBID|CHAIN|SEQUENCE  .........N........K....IVPR--
2AJY:H|PDBID|CHAIN|SEQUENCE  .........N........K....IVPR--
2AJZ:H|PDBID|CHAIN|SEQUENCE  .........N........K....IVPR--
2AK1:H|PDBID|CHAIN|SEQUENCE  .........N........K....IVPR--
3CFB:H|PDBID|CHAIN|SEQUENCE  ....E....................P---
3CFC:H|PDBID|CHAIN|SEQUENCE  .........................P---
3CFD:H|PDBID|CHAIN|SEQUENCE  ....E....................PS--
3CFE:H|PDBID|CHAIN|SEQUENCE  ....E....................PS--
3LS4:H|PDBID|CHAIN|SEQUENCE  ....E....N........K....IVPRDC
3LS5:H|PDBID|CHAIN|SEQUENCE  ....E....N........K....IVPRDC
4OCX:H|PDBID|CHAIN|SEQUENCE  ....E....N........K....IVPR--
```

```
4OCY:H|PDBID|CHAIN|SEQUENCE ....E....N........K....IVPR--
```

Table S3.4 Hapten_Mouse_Light chain_Kappa_28 sequences

All constant domains (Ck) are of mouse kappa type

```
                                    10        20        30        40        50        60        70        80        90       100
                            ....|....|....|....|....|....|....|....|....|....|....|....|....|....|....|....|....|....|....|....|
1CGS:L|PDBID|CHAIN|SEQUENCE ELVMTQSPLSLPVSLGDQASISCRPSQSLVHSNGNTYLHWYLQKPGQSPKLLIYRVSNRFSGVPDRFSGSGSGTAFTLKISRVEAEDLGVYFCSQGTHVP
2CGR:L|PDBID|CHAIN|SEQUENCE ....................................................................................................
1I9I:L|PDBID|CHAIN|SEQUENCE DV.V..T.................S...I......S..E...............K...................D................Y.F..S...
1I9J:L|PDBID|CHAIN|SEQUENCE DV.V..T.................S...I......S..E...............K...................D................Y.F..S...
1JNL:L|PDBID|CHAIN|SEQUENCE QI....T.A..SA.V.ETVT.T..A.GNIY-----N..A..Q..Q.K..Q..V.NAKTLVD...L.........QYS...NSLQP..F.N.Y.HHFWNT.
1JNN:L|PDBID|CHAIN|SEQUENCE QI....T.A..SA.V.ETVT.T..A.GNIY-----N..A..Q..Q.K..Q..V.NAKTLVD...L.........QYS...NSLQP..F.N.Y.HHFWNT.
1KEL:L|PDBID|CHAIN|SEQUENCE DVL...T.................F...I.........E.....S.........K...................D................Y.F..S...
1KEM:L|PDBID|CHAIN|SEQUENCE DVL...T.................F...I.........E.....S.........K...................D................Y.F..S...
1Q72:L|PDBID|CHAIN|SEQUENCE DI....AAP.V..TP.ESV.....S.K..L....Y.....F..R.....Q........LA..................RF.......V...Y.M.HLEY.
1QYG:L|PDBID|CHAIN|SEQUENCE DI....AAP.V..TP.ESV.....S.K..L....Y.....F..R.....Q........LA..................RF.......V...Y.M.HLEY.
1RFD:L|PDBID|CHAIN|SEQUENCE DI....AAP.V..TP.ESV.....S.K..L....Y.....F..R.....Q........LA..................RF.......V...Y.M.HLEY.
1UB5:B|PDBID|CHAIN|SEQUENCE DI....AAF.N..T..TS......STK..L....I...Y..........Q....QM..LA....N...S.....D...R........V...Y.A.NLEL.
1UB6:L|PDBID|CHAIN|SEQUENCE -AAL....V.N..T..TS......STK..L....I...Y..........Q....QM..LA....N...S.....D...R.NT.....V...Y.A.NLEL.
2AJS:L|PDBID|CHAIN|SEQUENCE DI.I..DE..N..TS.ESV.....S.R..LYKD.R...N.F..R.....Q....LM.T.A...S..........D...E....K...V...Y.Q.FVEY.
2AJU:L|PDBID|CHAIN|SEQUENCE DI.I..DE..N..TS.ESV.....S.R..LYKD.R...N.F..R.....Q....LM.T.A...S..........D...E....K...V...Y.Q.FVEY.
2AJV:L|PDBID|CHAIN|SEQUENCE DI.I..DE..N..TS.ESV.....S.R..LYKD.R...N.F..R.....Q....LM.T.A...S..........D...E....K...V...Y.Q.FVEY.
2AJX:L|PDBID|CHAIN|SEQUENCE DI.I..DE..N..TS.ESV.....S.R..LYKD.R...N.F..R.....Q....LM.T.A...S..........D...E....K...V...Y.Q.FVEY.
2AJY:L|PDBID|CHAIN|SEQUENCE DI.I..DE..N..TS.ESV.....S.R..LYKD.R...N.F..R.....Q....LM.T.A...S..........D...E....K...V...Y.Q.FVEY.
2AJZ:A|PDBID|CHAIN|SEQUENCE DI.I..DE..N..TS.ESV.....S.R..LYKD.R...N.F..R.....Q....LM.T.A...S..........D...E....K...V...Y.Q.FVEY.
2AK1:L|PDBID|CHAIN|SEQUENCE DI.I..DE..N..TS.ESV.....S.R..LYKD.R...N.F..R.....Q....LM.T.A...S..........D...E....K...V...Y.Q.FVEY.
3CFB:L|PDBID|CHAIN|SEQUENCE DI....AAF.N..T..TS......STK..L....I...Y..........Q....QM..LA........S.....D...R........V...Y.A.NLEL.
3CFC:L|PDBID|CHAIN|SEQUENCE DI....AAF.N..T..TS......STK..L....I...Y..........Q....QM..LA........S.....D...R........V...Y.A.NLEL.
3CFD:L|PDBID|CHAIN|SEQUENCE DIQ...TTS..SA....RVT....A..DIS-----N..N..Q...DGTV.....YT.RLH....S.........DYS.T..NLDQD.IAT...Q...TL.
3CFE:L|PDBID|CHAIN|SEQUENCE DIQ...TTS..SA....RVT....A..DIS-----N..N..Q...DGTV.....YT.RLH....S.........DYS.T..NLDQD.IAT...Q...TL.
3LS4:L|PDBID|CHAIN|SEQUENCE DI.L....TTMAA.P.EKIT.T.SA.S.ISS----N.....Q....F........T..LA....A.........SYS.T.GTM....VAT.Y.Q..SSI.
3LS5:L|PDBID|CHAIN|SEQUENCE DI.L....TTMAA.P.EKIT.T.SA.S.ISS----N.....Q....F........T..LA....A.........SYS.T.GTM....VAT.Y.Q..SSI.
4OCX:L|PDBID|CHAIN|SEQUENCE DVLL..I.................S...I.........E...............K..T................D................Y.F..S...
4OCY:L|PDBID|CHAIN|SEQUENCE DVLL..I.................S...I.........E...............K..T................D................Y.F..S...

                                   110       120       130       140       150       160       170       180       190       200
                            ....|....|....|....|....|....|....|....|....|....|....|....|....|....|....|....|....|....|....|....|
1CGS:L|PDBID|CHAIN|SEQUENCE YTFGGGTKLELKRADAAPTVSIFPPSSEQLTSGGASVVCFLNNFYPKDINVKWKIDGSERQNGVLNSWTDQDSKDSTYSMSSTLTLTKDEYERHNSYTCE
2CGR:L|PDBID|CHAIN|SEQUENCE ....................................................................................................
1I9I:L|PDBID|CHAIN|SEQUENCE P.........I.........................................................................................
1I9J:L|PDBID|CHAIN|SEQUENCE P.........I.........................................................................................
1JNL:L|PDBID|CHAIN|SEQUENCE ..........I.........................................................................................
1JNN:L|PDBID|CHAIN|SEQUENCE ..........I.........................................................................................
1KEL:L|PDBID|CHAIN|SEQUENCE R.........I.........................................................................................
1KEM:L|PDBID|CHAIN|SEQUENCE R.........I.........................................................................................
1Q72:L|PDBID|CHAIN|SEQUENCE F...S.....I...................................Q..T.S.....A..SS...........S....................S.....
1QYG:L|PDBID|CHAIN|SEQUENCE F...S.....I...................................Q..T.S.....A..SS...........S....................S.....
1RFD:L|PDBID|CHAIN|SEQUENCE F...S.....I...................................Q..T.S.....A..SS...........S....................S.....
```

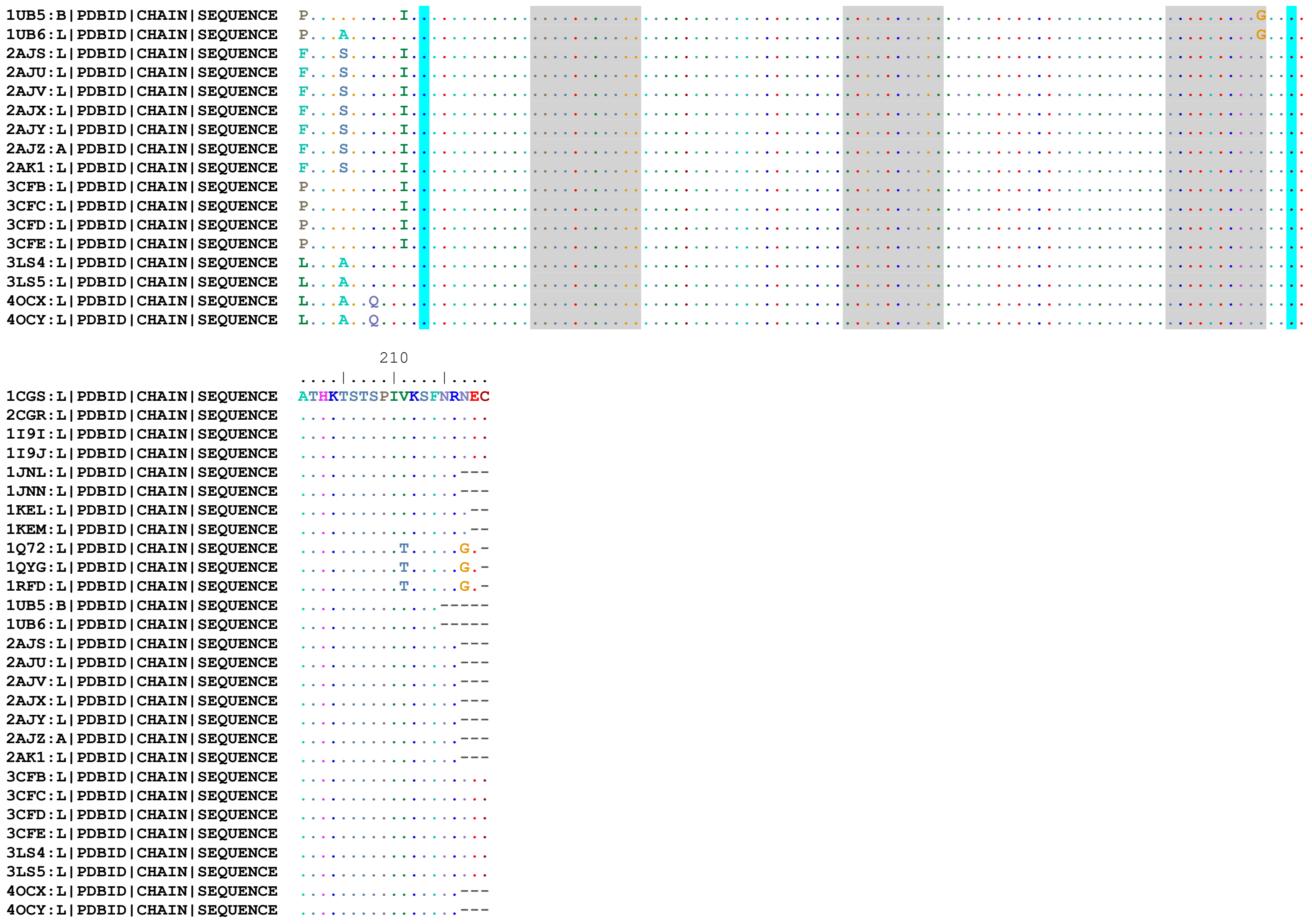
1UB5:B|PDBID|CHAIN|SEQUENCE
1UB6:L|PDBID|CHAIN|SEQUENCE
2AJS:L|PDBID|CHAIN|SEQUENCE
2AJU:L|PDBID|CHAIN|SEQUENCE
2AJV:L|PDBID|CHAIN|SEQUENCE
2AJX:L|PDBID|CHAIN|SEQUENCE
2AJY:L|PDBID|CHAIN|SEQUENCE
2AJZ:A|PDBID|CHAIN|SEQUENCE
2AK1:L|PDBID|CHAIN|SEQUENCE
3CFB:L|PDBID|CHAIN|SEQUENCE
3CFC:L|PDBID|CHAIN|SEQUENCE
3CFD:L|PDBID|CHAIN|SEQUENCE
3CFE:L|PDBID|CHAIN|SEQUENCE
3LS4:L|PDBID|CHAIN|SEQUENCE
3LS5:L|PDBID|CHAIN|SEQUENCE
4OCX:L|PDBID|CHAIN|SEQUENCE
4OCY:L|PDBID|CHAIN|SEQUENCE
210
....|....|....|....
1CGS:L|PDBID|CHAIN|SEQUENCE ATHKTSTSPIVKSFNRNEC
2CGR:L|PDBID|CHAIN|SEQUENCE ....
1I9I:L|PDBID|CHAIN|SEQUENCE ....
1I9J:L|PDBID|CHAIN|SEQUENCE ....
1JNL:L|PDBID|CHAIN|SEQUENCE ....---
1JNN:L|PDBID|CHAIN|SEQUENCE ....---
1KEL:L|PDBID|CHAIN|SEQUENCE ....--
1KEM:L|PDBID|CHAIN|SEQUENCE ....--
1Q72:L|PDBID|CHAIN|SEQUENCE ..........T.....G.-
1QYG:L|PDBID|CHAIN|SEQUENCE ..........T.....G.-
1RFD:L|PDBID|CHAIN|SEQUENCE ..........T.....G.-
1UB5:B|PDBID|CHAIN|SEQUENCE ....-----
1UB6:L|PDBID|CHAIN|SEQUENCE ....-----
2AJS:L|PDBID|CHAIN|SEQUENCE ....---
2AJU:L|PDBID|CHAIN|SEQUENCE ....---
2AJV:L|PDBID|CHAIN|SEQUENCE ....---
2AJX:L|PDBID|CHAIN|SEQUENCE ....---
2AJY:L|PDBID|CHAIN|SEQUENCE ....---
2AJZ:A|PDBID|CHAIN|SEQUENCE ....---
2AK1:L|PDBID|CHAIN|SEQUENCE ....---
3CFB:L|PDBID|CHAIN|SEQUENCE ....
3CFC:L|PDBID|CHAIN|SEQUENCE ....
3CFD:L|PDBID|CHAIN|SEQUENCE ....
3CFE:L|PDBID|CHAIN|SEQUENCE ....
3LS4:L|PDBID|CHAIN|SEQUENCE ....
3LS5:L|PDBID|CHAIN|SEQUENCE ....
4OCX:L|PDBID|CHAIN|SEQUENCE ....---
4OCY:L|PDBID|CHAIN|SEQUENCE ....---

Table S3.5 Hapten_Mouse_Light chain_Lambda_4 sequences

All constant domains (Cλ) are of mouse lambda type

```
                                   10        20        30        40        50        60        70        80        90       100
                           ....|....|....|....|....|....|....|....|....|....|....|....|....|....|....|....|....|....|....|....|
1NGP:L|PDBID|CHAIN|SEQUENCE QAVVTQESALTTSPGETVTLTCRSSTGAVTTSNYANWVQEKPDHLFTGLIGGTNNRAPGVPARFSGSLIGDKAALTITGAQTEDEAIYFCALWYSNHWVF
1NGQ:L|PDBID|CHAIN|SEQUENCE ....................................................................................................
1Q0X:L|PDBID|CHAIN|SEQUENCE D............................................................................................SN.KL..
1Q0Y:L|PDBID|CHAIN|SEQUENCE D............................................................................................SN.KL..

                                  110       120       130       140       150       160       170       180       190       200
                           ....|....|....|....|....|....|....|....|....|....|....|....|....|....|....|....|....|....|....|....|
1NGP:L|PDBID|CHAIN|SEQUENCE GGGTKLTVLGQPKSSPSVTLFPPSSEELETNKATLVCTITDFYPGVVTVDWKVDGTPVTQGMETTQPSKQSNNKYMASSYLTLTARAWERHSSYSCQVTH
1NGQ:L|PDBID|CHAIN|SEQUENCE ....................................................................................................
1Q0X:L|PDBID|CHAIN|SEQUENCE ................T...........S.A............................A........................................
1Q0Y:L|PDBID|CHAIN|SEQUENCE ................T...........S.A............................A........................................

                                  210
                           ....|....|....|
1NGP:L|PDBID|CHAIN|SEQUENCE EGHTVEKSLSRADCS
1NGQ:L|PDBID|CHAIN|SEQUENCE ...............
1Q0X:L|PDBID|CHAIN|SEQUENCE ...SSN.T....---
1Q0Y:L|PDBID|CHAIN|SEQUENCE ...SSN.T....---
```

Supplementary S4: Angles and distances

Angles and domains packing were measured as described in the Methods section. Distances were measured in angstrom (Å).

PDB number	PDB ID	Crystal form	Linker to linker distance	c-c (light)	c-c (heavy)	Light angel	Heavy angel	Magnitude of average angle change
1	3CFB	Antigen-antibody	42.6	41.4	35.4	106	84.4	9.25
2	3CFC	Antibody	42.9	43.3	31.8	112.9	72.8	
3	3CFD	Antigen-antibody	40.7	42.9	32.9	109.1	76.8	0.55
4	3CFE	Antibody	40.6	42.4	33	108.3	77.1	
5	1UB5	Antigen-antibody	43	42.9	33.4	110.8	76.7	0.95
6	1UB6	Antibody	42.2	42.6	33.1	112	77.4	
7	1Q72	Antigen-antibody	41.3	42.2	31.5	109	73.7	0.75
8	1QYG	Antigen-antibody	41.3	42.1	31.5	108.2	73.9	0.45

9	1RFD	Antibody	41.6	42	31.4	107.5	73.7	
10	1JNN	Antigen-antibody	40.4	43.4	32.4	113.8	76.7	0.75
11	1JNL	Antibody	41.3	44.1	33.4	114.4	77.6	
12	1KEL	Antigen-antibody	40.3	38.4	40.9	95	104.8	1
13	1KEM	Antibody	39.7	38.4	41.4	95.4	106.4	
14	1NGP	Antigen-antibody	41.1	33	40.6	90	105.4	8.55
15	1NGQ	Antibody	41.1	32.8	40.8	73.1	105.2	
16	2CGR	Antigen-antibody	40.9	42.8	34.2	109.9	80.3	19.75
17	1CGS	Antibody	39.9	37.5	41.4	95.3	105.2	
18	1Q0Y	Antigen-antibody	40	38.6	40.6	89.5	97.1	0.65
19	1Q0X	Antibody	39.9	38.5	41	89	97.9	
20	2AJS	Antigen-antibody	41.5	42.7	30.2	110.6	70	2.35

21	2AJV	Antigen-antibody	41	42.4	31.2	109.8	73.8	0.95
22	2AJX	Antigen-antibody	41.1	43.4	29.2	113.2	67.9	4.7
23	2AJY	Antigen-antibody	41.5	43.2	29.7	112.6	68.3	4.2
24	2AJZ	Antigen-antibody	41.8	43	30	108.8	69.2	1.85
25	2AK1	Antigen-antibody	41.2	43.3	29.4	112.5	68.3	4.15
26	2AJU	Antibody	41.3	42.4	31	108.8	72.9	
27	4OCX	Antigen-antibody	40.8	41.1	36.8	103.1	90.5	1.25
28	4OCY	Antibody	40.8	40.4	37.2	102.2	92.1	
29	1I9J	Antigen-antibody	41.3	42.5	30.2	107.8	69.6	0.5
30	1I9I	Antibody	41.3	42.5	30.5	108.1	70.3	
31	3LS4	Antigen-antibody	41.2	41.1	34.2	101.4	81.7	1.25
32	3LS5	Antibody	41.1	40.9	33.5	101.8	79.6	

33	1FL6	Antigen-antibody	41.4	44.3	29.7	113.9	69.2	5.9
34	1FL5	Antibody	41.3	43.5	33.5	110.6	77.7	
35	1C5C	Antigen-antibody	39.8	39.4	38.8	98.5	97.3	0.5
36	1C5B	Antibody	39	39.6	38.7	98.8	98	
37	1D6V	Antigen-antibody	39.9	39.6	39.4	97.7	97.7	22.4
38	1D5B	Antibody	42.5	43.4	29	111.4	66.6	
39	1AJ7	Antigen-antibody	40.9	46.1	26.9	120.7	71.7	30.65
40	2RCS	Antibody	39.4	37.3	40	91.6	103.9	
